# Supplementary material for: Isolation and Structure Elucidation of New Cytotoxic Macrolides Halosmysins B and C from the Fungus Halosphaeriaceae sp. Associated with a Marine Alga
Source: Mar Drugs. 2022 Mar 25;20(4):226. doi: 10.3390/md20040226 (PMC9030429; doi:10.3390/md20040226)

Supplementary Materials

Isolation and structure elucidation of new cytotoxic macrolides halosmysins B and C from the fungus *Halosphaeriaceae* sp. associated with a marine alga

Takeshi Yamada *, Kanoko Yoshida, Takashi Kikuchi and Tomoya Hirano [1]

The Table of Contents

**Table S1**	**Spectral data including 2D NMR data for 2**	**1**
**Table S2**	**Spectral data including 2D NMR data for 3**	**2**
**Figure S1**	**14-membered macrodiolides associated to halosmysins**	**3**
**Figure S2**	**^{1}H NMR spectrum of 2 in $CDCl_3$**	**4**
**Figure S3**	**^{13}C NMR spectrum of 2 in $CDCl_3$**	**10**
**Figure S4**	**^{1}H-^{1}H COSY of 2**	**13**
**Figure S5**	**NOESY of 2**	**16**
**Figure S6**	**HMQC of 2**	**19**
**Figure S7**	**HMBC of 2**	**21**
**Figure S8**	**FABMS of 2**	**26**
**Figure S9**	**IR spectrum of 2**	**29**
**Figure S10**	**^{1}H NMR spectrum of 3 in MeOH-d_4**	**30**
**Figure S11**	**^{13}C NMR spectrum of 3 in MeOH-d_4**	**35**
**Figure S12**	**^{1}H-^{1}H COSY of 3**	**39**
**Figure S13**	**NOESY of 3**	**42**
**Figure S14**	**HMQC of 3**	**45**
**Figure S15**	**HMBC of 3**	**48**
**Figure S16**	**FABMS of 3**	**52**
**Figure S17**	**IR spectrum of 3**	**55**
**Figure S18**	**HPLC chromatograms of halosmycins A, B, and C**	**56**
**Figure S19**	**^{1}H NMR spectrum of 5-hydroxy-(2*E*)-hexenoic acid in MeOH-d_4**	**57**
**Figure S20**	**^{1}H NMR spectrum of 4,5,7-trihydroxy-(2E)-octenoic acid in MeOH-d_4**	**58**

Table S1 NMR spectral data of **2** in $CDCl_3$

Position	δ_H[a]		J/Hz	^{1}H-^{1}H COSY	NOESY[b]	δ_C		HMBC (C)[c]
2						166.2	(s)	
3	5.66	d	16.4 (4)	4	5β, 9	124.6	(d)	2, 5
4	6.83	ddd	16.4 (3), 10.8 (5β), 6.0 (5α)	3, 5α, 5β	6	145.2	(d)	2
5α	2.53	ddd	13.2 (5β), 6.0 (4), 1.8 (6)	4, 5β	15	40.0	(t)	3
5β	2.25	ddd	13.2 (5α), 12.6 (6), 10.8 (4)	4, 5α, 6	3, 15			3, 4, 6
6	5.31	dqd	12.6 (5β), 6.6 (15), 1.8 (5α)	5β, 15	4	69.9	(d)	
8						169.0	(s)	
9	3.59	d	3.6 (10)	10	3, 1', 7'A	52.6	(d)	8, 11, 5', 6'
10	4.81	d	3.6 (9)	9	12, S-CH_3, 4'	52.4	(d)	8, 11, 2', 3'
11						208.0	(s)	
12	4.56	dd	9.0 (12-OH), 8.4 (13α)	13α, 12-OH	10	75.1	(d)	11, 14
13α	1.93	ddd	15.0 (13β), 8.4 (12), 2.4 (14)	12, 13β	16	37.3	(t)	11
13β	2.60	ddd	15.0 (13α), 11.4 (14), 1.2 (12)	13α, 14	16			14
14	5.25	dqd	11.4 (13β), 6.6 (16), 2.4 (13α)	13β, 16		65.8	(d)	
15	1.40	d	6.6 (6)	6	5α, 5β	20.5	(q)	5, 6
16	1.27	d	6.6 (14)	14	13α, 13β	20.3	(q)	13, 14
12-OH	3.31	br d	9.0 (12)	12				
1'(NH)	5.84	s			9, 9'			3', 5', 6', 7'
2'						165.4	(s)	
3'						68.6	(s)	
4'(NH)	6.26	s			10, S-CH_3,			6'
5'						169.5	(s)	
6'						62.2	(s)	
7'A	2.99	d	14.4 (7'B)	7'B	9, 9'	34.2	(t)	9, 5', 6', 8', 9'
7'B	3.56	d	14.4 (7'A)	7'A	9'			9, 5', 6', 8', 9'
8'						125.0	(s)	
9'	6.90	d	8.4 (10')	10'	1', 7'A, 7'B	131.6	(d)	7', 11'
10'	7.30	d	8.4 (9')	9'	12'	115.6	(d)	8', 11'
11'						158.7	(s)	
12'	4.51	d	6.0 (13')	13'	10', 16'	64.8	(t)	11', 13', 14'
13'	5.50	br t	6.0 (12')	12'	15'	119.5	(d)	16'
14'						138.5	(s)	
15'	1.81	s			13'	25.8	(q)	13', 14', 16'
16'	1.76	s			12'	18.2	(q)	13', 14', 15'
S-CH_3	2.34	s			10, 4'	12.4	(q)	3'

a ^{1}H chemical shift values (δ ppm from $SiMe_4$) followed by multiplicity and then the coupling constants (J /Hz). Figures in parentheses indicate the proton coupling with that position. b The correlations with geminal and vicinal protones are removed. c Long range ^{1}H-^{13}C correlations from H to C observed in the HMBC experiment.

Table S2 NMR spectral data of **3** in MeOH-d_4

Position	δ_H[a]		J/Hz	^{1}H-^{1}H COSY	NOESY[b]	δ_C		HMBC (C)[c]
2						166.9	(s)	
3	5.78	d	16.2 (4)	4	5α	126.8	(d)	2, 5
4	6.68	ddd	16.4 (3), 12.0 (5α), 4.2 (5β)	3, 5α, 5β	6	146.0	(d)	2, 5
5α	2.27	ddd	13.2 (5β), 12.0 (4), 12.0 (6)	4, 5β, 6	3, 15	42.0	(t)	3, 4, 6, 15
5β	2.58	ddd	13.2 (5α), 4.2 (6), 4.2 (4)	4, 5α	15			
6	5.29	dqd	12.0 (5α), 6.0 (15), 4.2 (5β)	5α, 15	4	70.1	(d)	
8						167.8	(s)	
9	6.08	dd	15.6 (10), 0.6 (11)	10	11, 5'	125.2	(d)	8, 11
10	6.77	dd	15.6 (9), 6.0 (11)	9, 11	12, 13α	147.2	(d)	8, 9, 11
11	3.96	ddd	9.0 (12), 6.0 (11), 0.6 (9)	10, 12	9, 13α, 1'	83.8	(d)	9, 10, 12, 1'
12	3.76	ddd	9.0 (11), 6.6 (13β), 1.2 (13α)	13β	10, 16	72.6	(d)	10, 11, 13, 14
13α	1.92	dd	16.2 (13β), 4.2 (14)	14, 13β	10, 11	37.1	(t)	11, 14, 16
13β	1.48	ddd	16.2 (13α), 6.6 (12), 2.4 (14)	12, 13α	16			12
14	5.13	qdd	6.6 (16), 4.2 (13α), 2.4 (13β)	13α, 16		69.7	(d)	12
15	1.36	d	6.0 (6)	6	5α, 5β	20.6	(q)	5, 6
16	1.33	d	6.6 (14)	14	12, 13β	18.4	(q)	13, 14
1'	5.00	d	3.6 (2')	2'	11	102.9	(d)	11, 2', 5'
2'	3.43	dd	9.6 (3'), 3.6 (1')	1', 3'	4'	73.9	(d)	3'
3'	3.67	dd	9.6 (2'), 9.6 (4')	2', 4'	5'	75.1	(d)	2', 4', 5'
4'	3.34	dd	10.2 (5'), 9.6 (3')	3', 5'	2', 6'	71.4	(d)	3', 5', 6'
5'	3.55	dt	10.2 (4'), 3.6 (6')	4', 6'	9, 3'	74.3	(d)	
6'	3.64	d	3.6 (5')	5'	4'	62.1	(t)	4', 5'

a ^{1}H chemical shift values (δ ppm from $SiMe_4$) followed by multiplicity and then the coupling constants (*J* /Hz). Figures in parentheses indicate the proton coupling with that position. b The correlations with geminal and vicinal protones are removed. c Long range ^{1}H-^{13}C correlations from H to C observed in the HMBC experiment.

Figure S1 14-membered macrodiolides associated to halosmysins

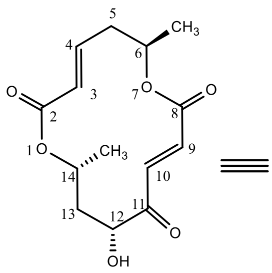

colletoketol

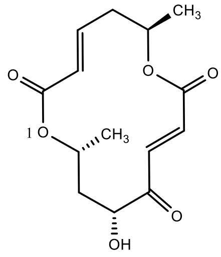

grahamimycin A

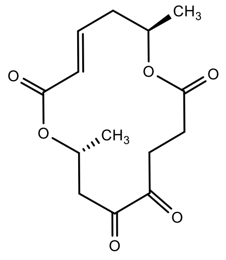

grahamimycin A_1

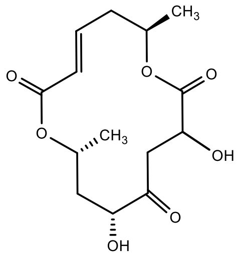

grahamimycin B

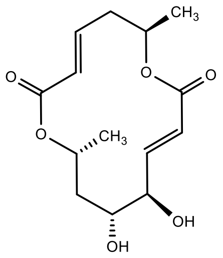

(6*R*,11*R*,12*R*,14*R*)-colletodiol

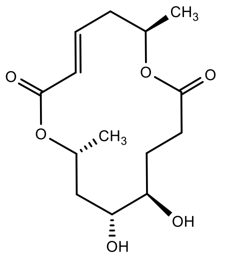

9,10-dihydro-(6*R*,11*R*,12*R*,14*R*)-colletodiol

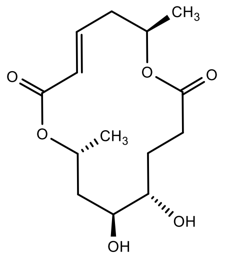

9,10-dihydro-(6*R*,11*S*,12*S*,14*R*)-colletodiol

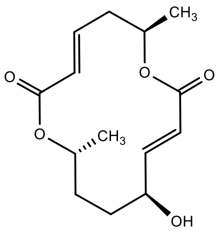

colletallol

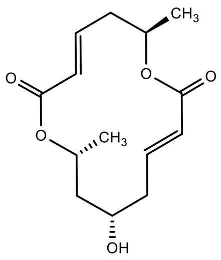

colletol

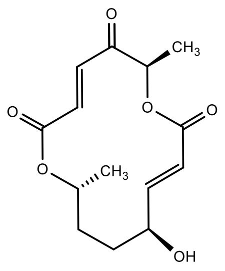

acremonol

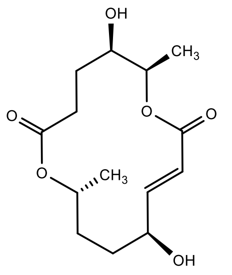

acremodiol

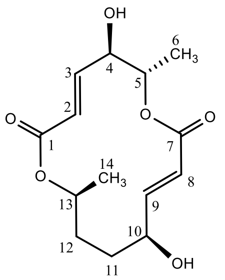

clonostachydiol

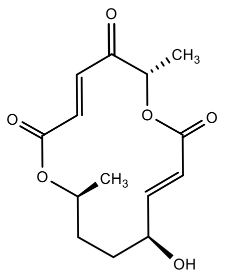

4-keto-clonostachydiol

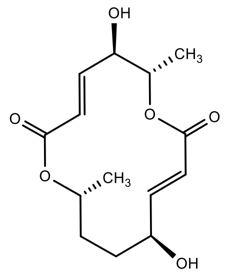

cordybislactone

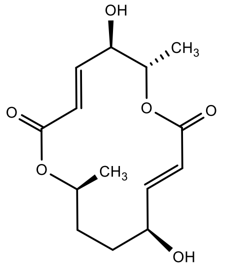

acaudiol A

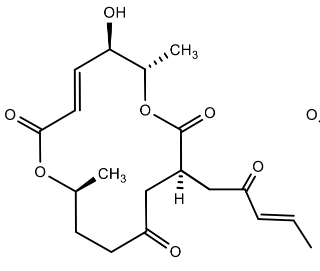

acaulone A

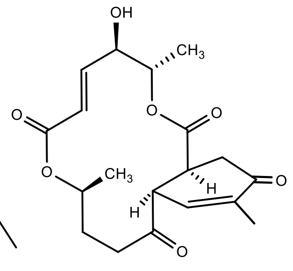

acaulone B

Figure S2 ^{1}H NMR spectrum of 2 in $CDCl_3$

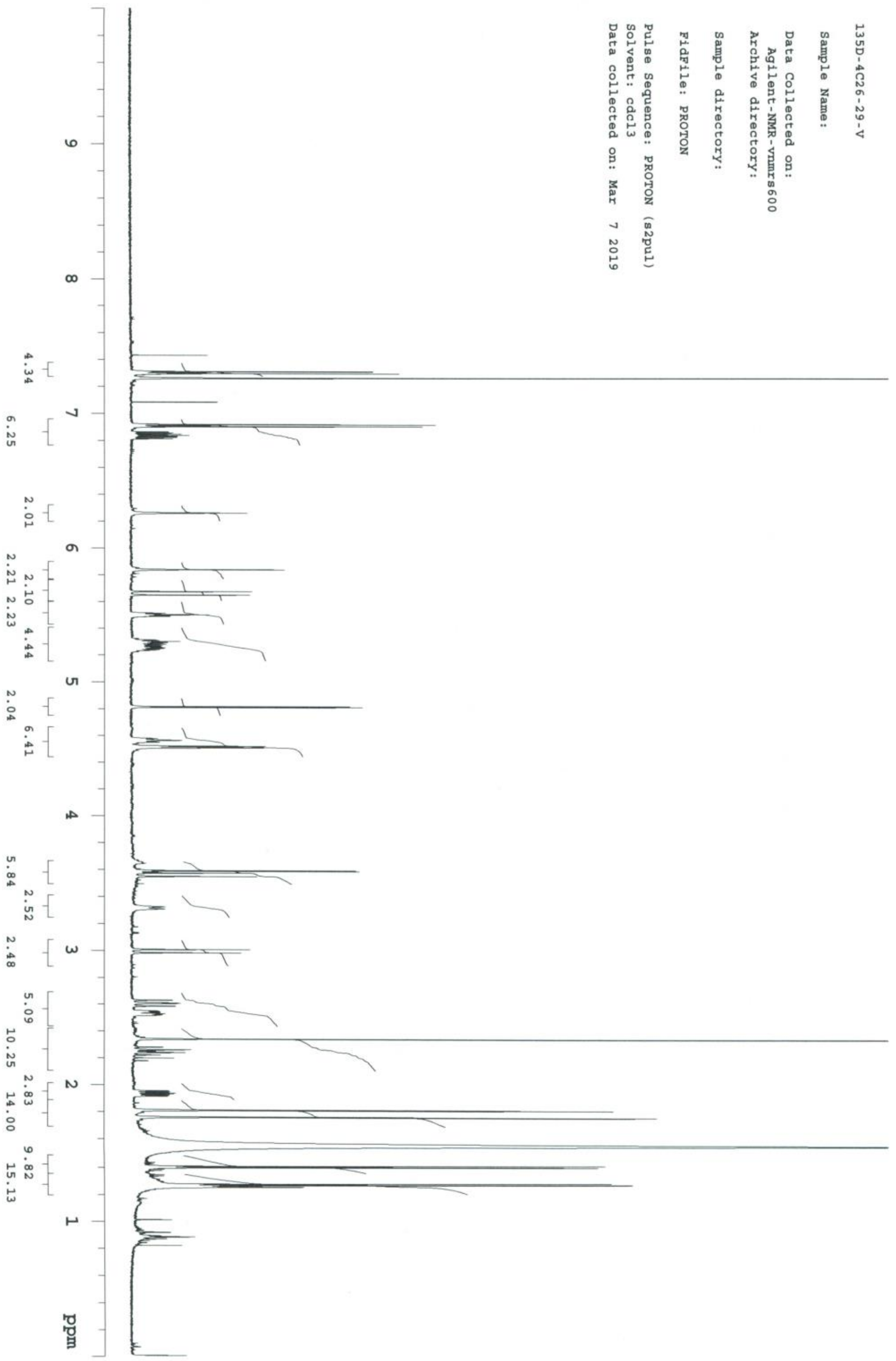

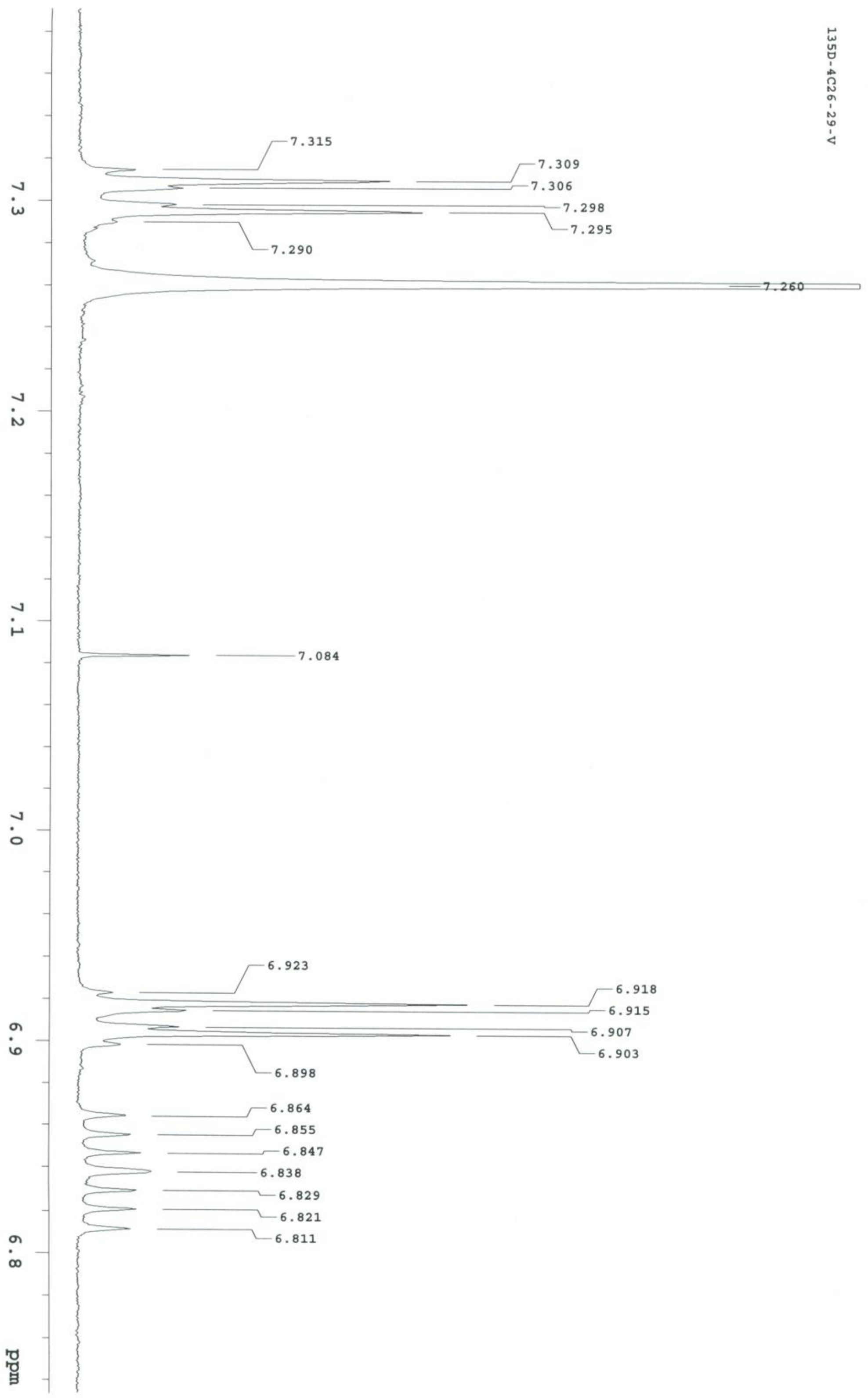
135D-4C26-29-V
7.315
7.309
7.306
7.298
7.295
7.290
7.260
7.084
6.923
6.918
6.915
6.907
6.903
6.898
6.864
6.855
6.847
6.838
6.829
6.821
6.811
7.3
7.2
7.1
7.0
6.9
6.8
ppm

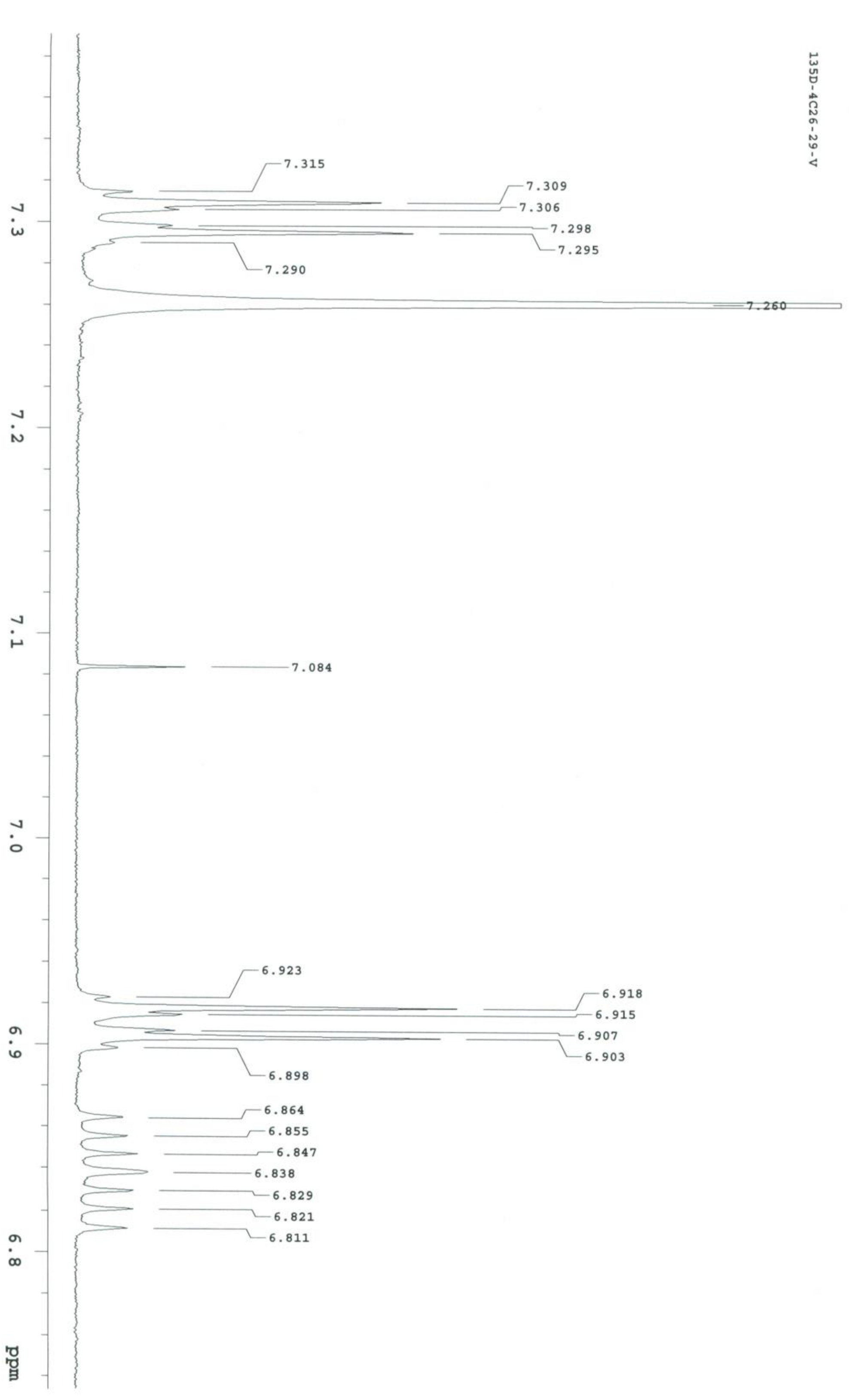
135D-4C26-29-V
7.315
7.309
7.306
7.298
7.295
7.290
7.260
7.084
6.923
6.918
6.915
6.907
6.903
6.898
6.864
6.855
6.847
6.838
6.829
6.821
6.811
7.3
7.2
7.1
7.0
6.9
6.8
ppm

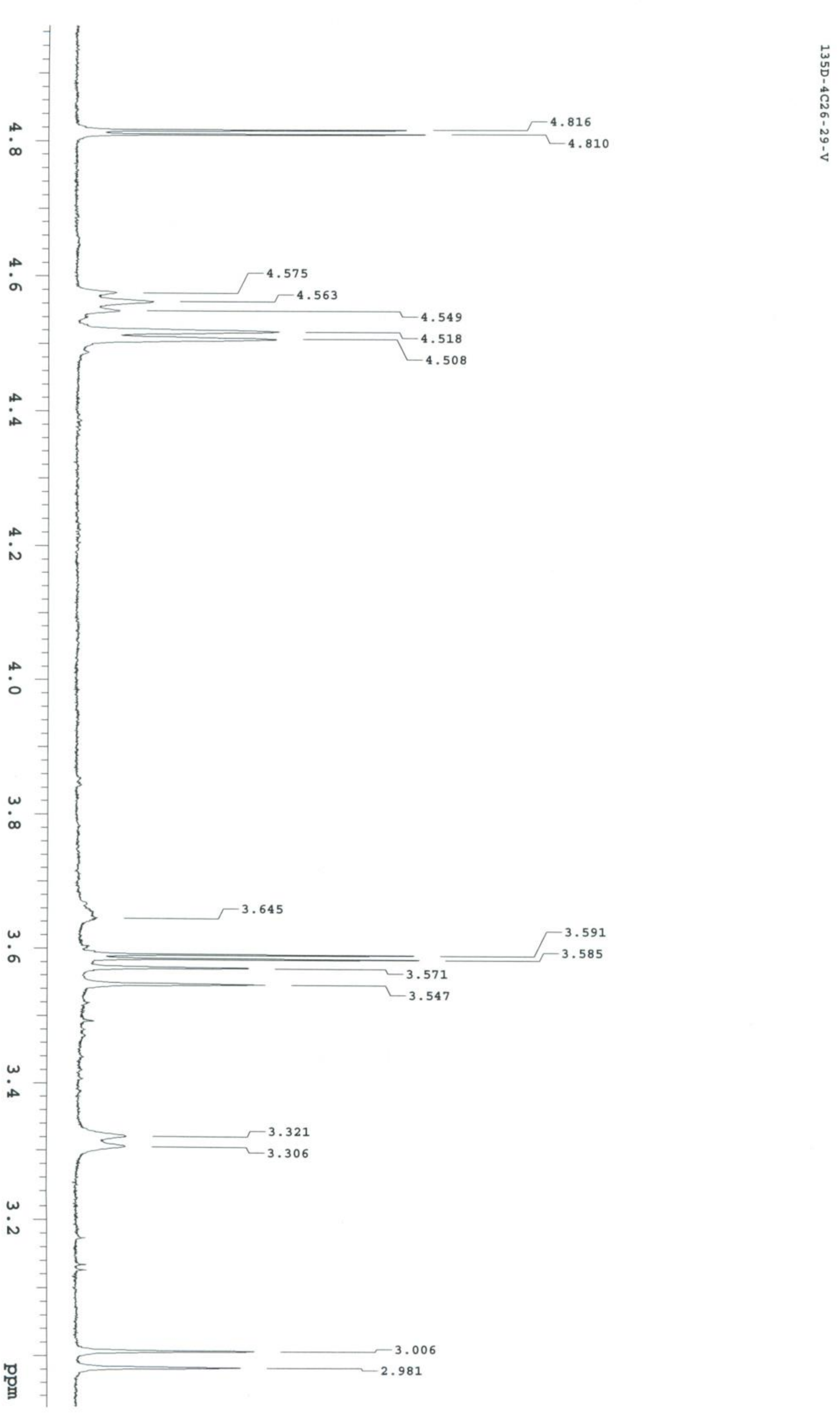
135D-4C26-29-V
4.816
4.810
4.575
4.563
4.549
4.518
4.508
3.645
3.591
3.585
3.571
3.547
3.321
3.306
3.006
2.981
4.8
4.6
4.4
4.2
4.0
3.8
3.6
3.4
3.2
ppm

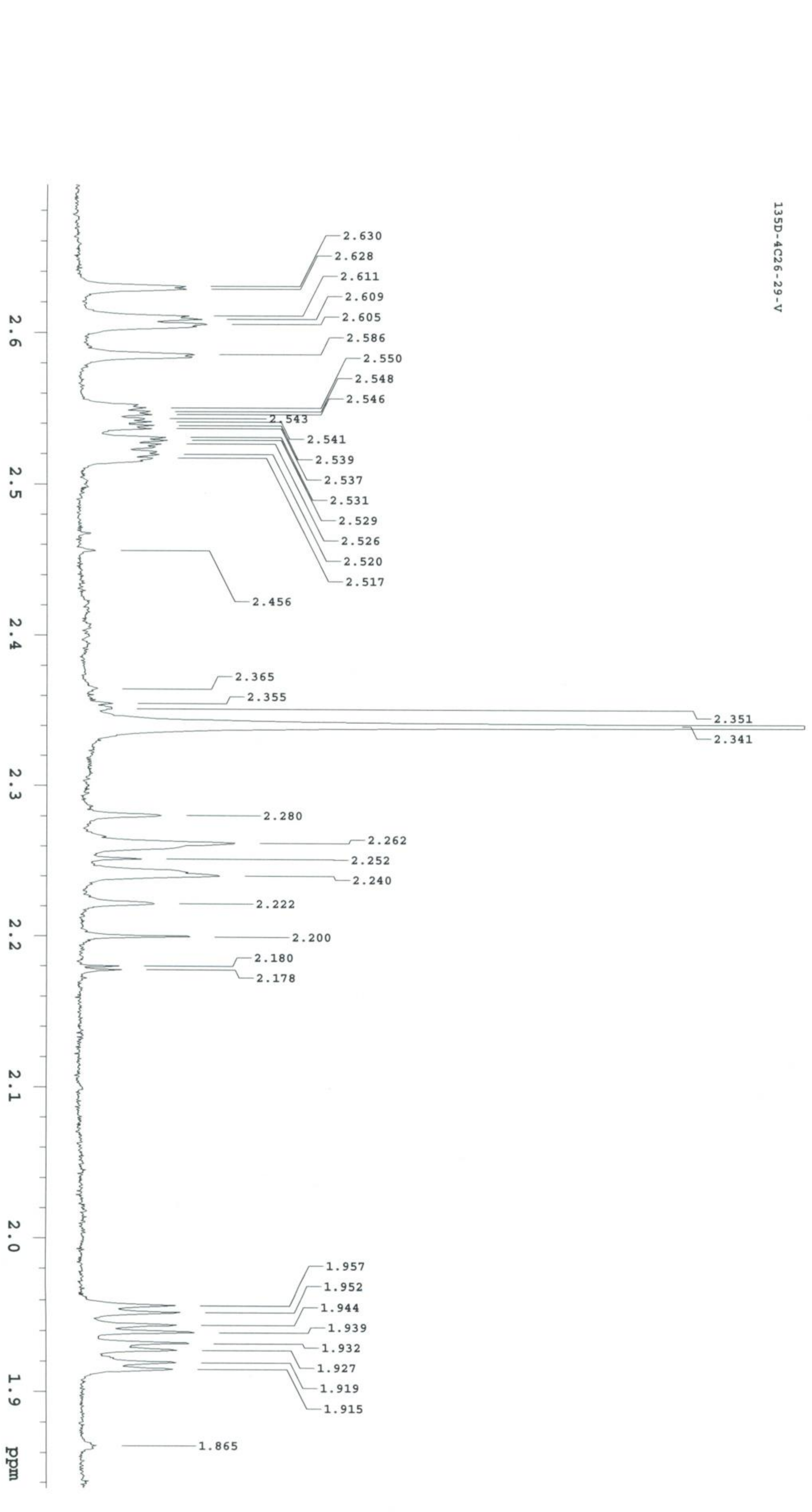
135D-4C26-29-V
2.630
2.628
2.611
2.609
2.605
2.586
2.550
2.548
2.546
2.543
2.541
2.539
2.537
2.531
2.529
2.526
2.520
2.517
2.456
2.365
2.355
2.351
2.341
2.280
2.262
2.252
2.240
2.222
2.200
2.180
2.178
1.957
1.952
1.944
1.939
1.932
1.927
1.919
1.915
1.865
2.6
2.5
2.4
2.3
2.2
2.1
2.0
1.9
ppm

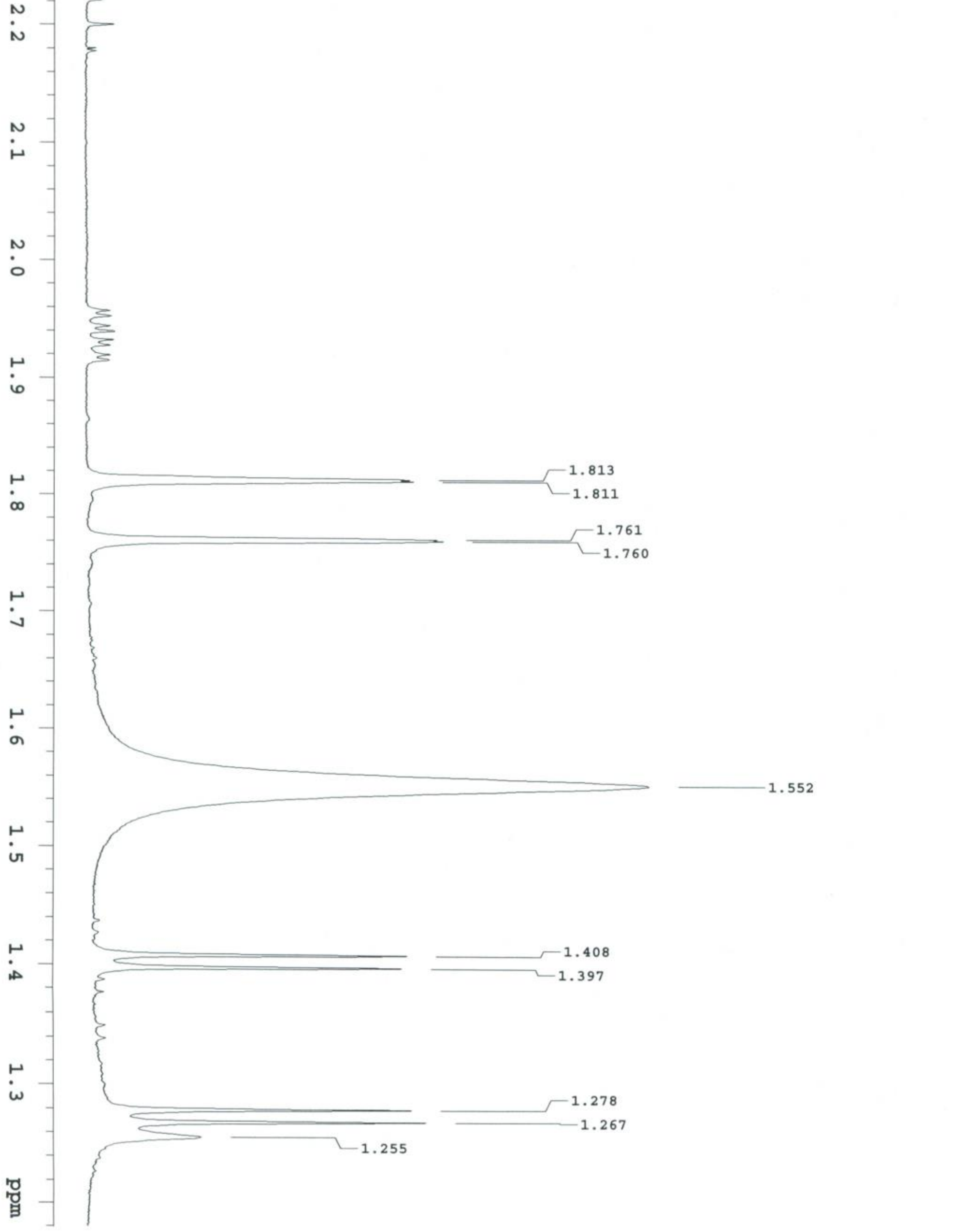
135D-4C26-29-V
2.341
1.813
1.811
1.761
1.760
1.552
1.408
1.397
1.278
1.267
1.255
2.4
2.3
2.2
2.1
2.0
1.9
1.8
1.7
1.6
1.5
1.4
1.3
ppm

Figure S3 ^{13}C NMR spectrum of 2 in $CDCl_3$

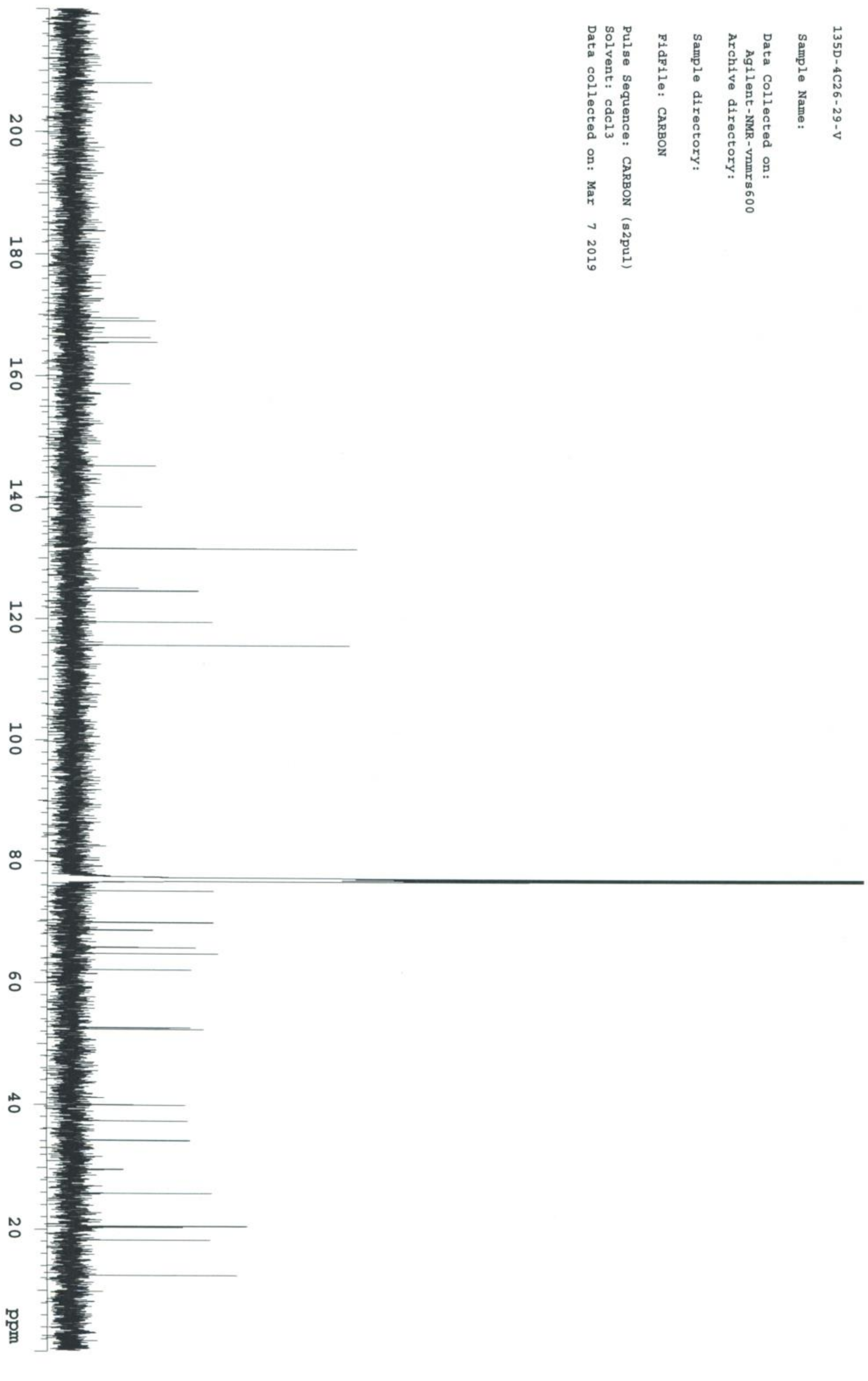

135D-4C26-29-V

INDEX	FREQUENCY	PPM	HEIGHT
1	31370.5	207.967	15.1
2	28865.5	191.360	-7.6
3	25561.7	169.458	12.5
4	25486.6	168.960	15.8
5	25069.3	166.194	14.9
6	24956.0	165.443	16.2
7	23937.6	158.691	10.9
8	23366.6	154.906	-6.9
9	21899.6	145.181	15.8
10	21126.3	140.054	-7.0
11	20891.6	138.498	13.2
12	19848.9	131.586	55.4
13	18862.9	125.049	12.5
14	18795.9	124.605	24.3
15	18021.4	119.470	27.0
16	17441.1	115.623	54.0
17	11646.2	77.207	3903.0
18	11615.0	77.000	4264.6
19	11582.6	76.785	4182.1
20	11324.8	75.077	27.4
21	10591.9	70.218	-6.6
22	10541.1	69.881	27.3
23	10351.5	68.624	15.5
24	9928.4	65.819	23.9
25	9777.0	64.815	28.3
26	9375.9	62.156	23.0
27	7939.0	52.631	22.9
28	7902.0	52.385	25.5
29	6028.2	39.963	21.9
30	5631.7	37.335	22.4
31	5154.3	34.170	22.9
32	4977.4	32.997	-6.4
33	4479.2	29.694	9.9
34	3896.6	25.832	27.1
35	3088.6	20.475	34.0
36	3059.7	20.284	21.4
37	2748.7	18.222	26.7
38	1871.3	12.406	32.0

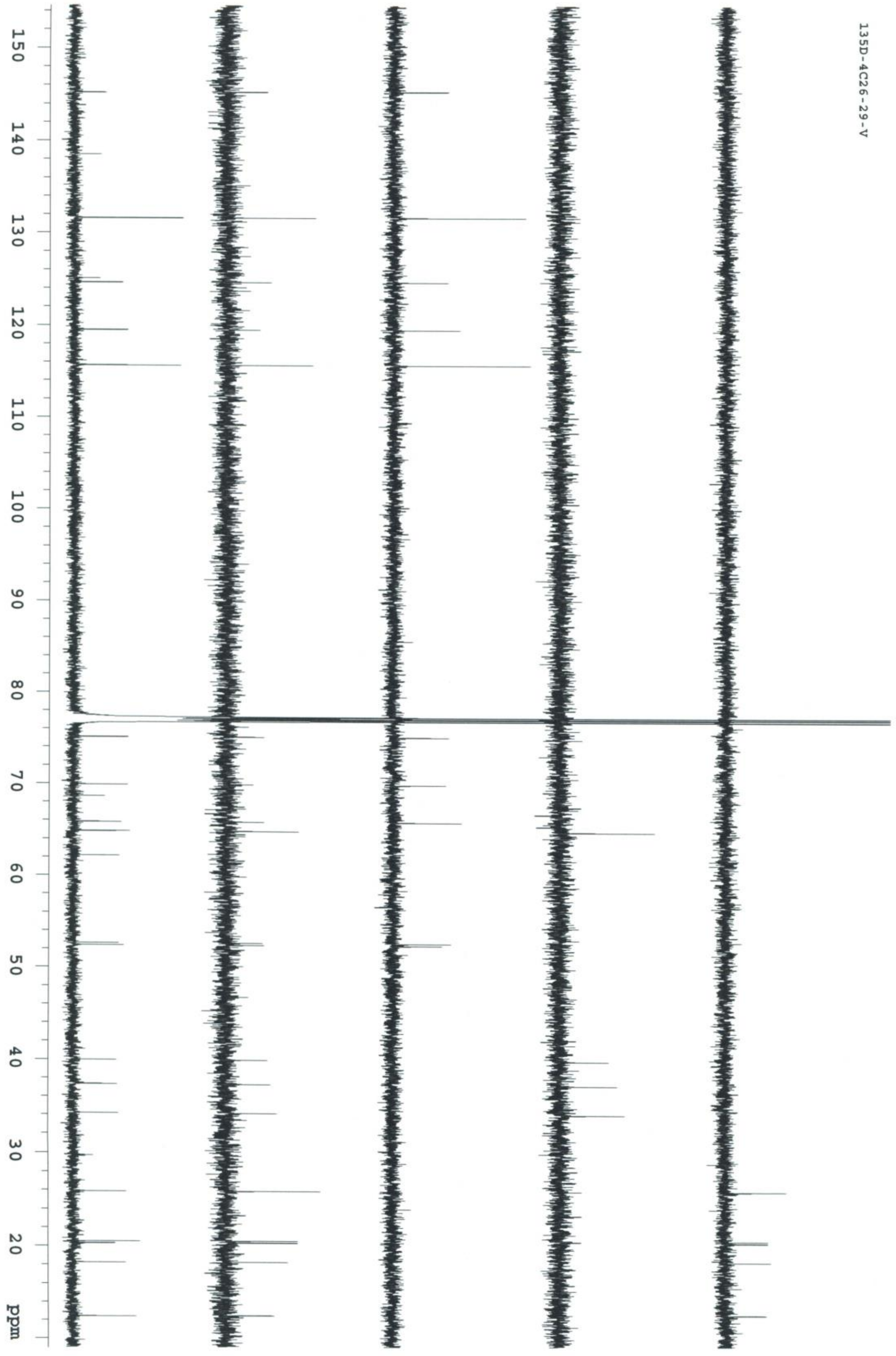
135D-4C26-29-V
150
140
130
120
110
100
90
80
70
60
50
40
30
20
ppm

Figure S4 ^{1}H-^{1}H COSY of 2

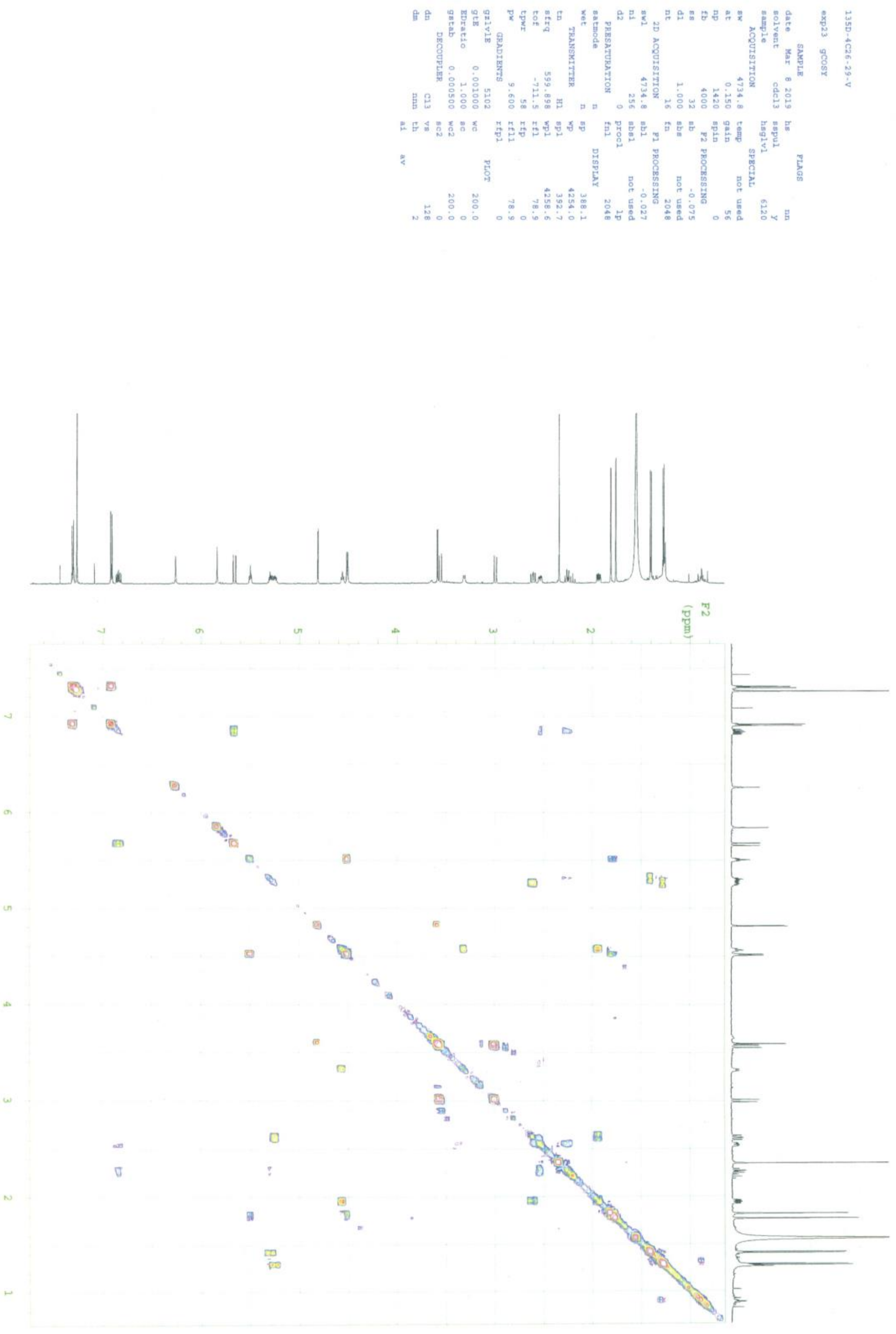

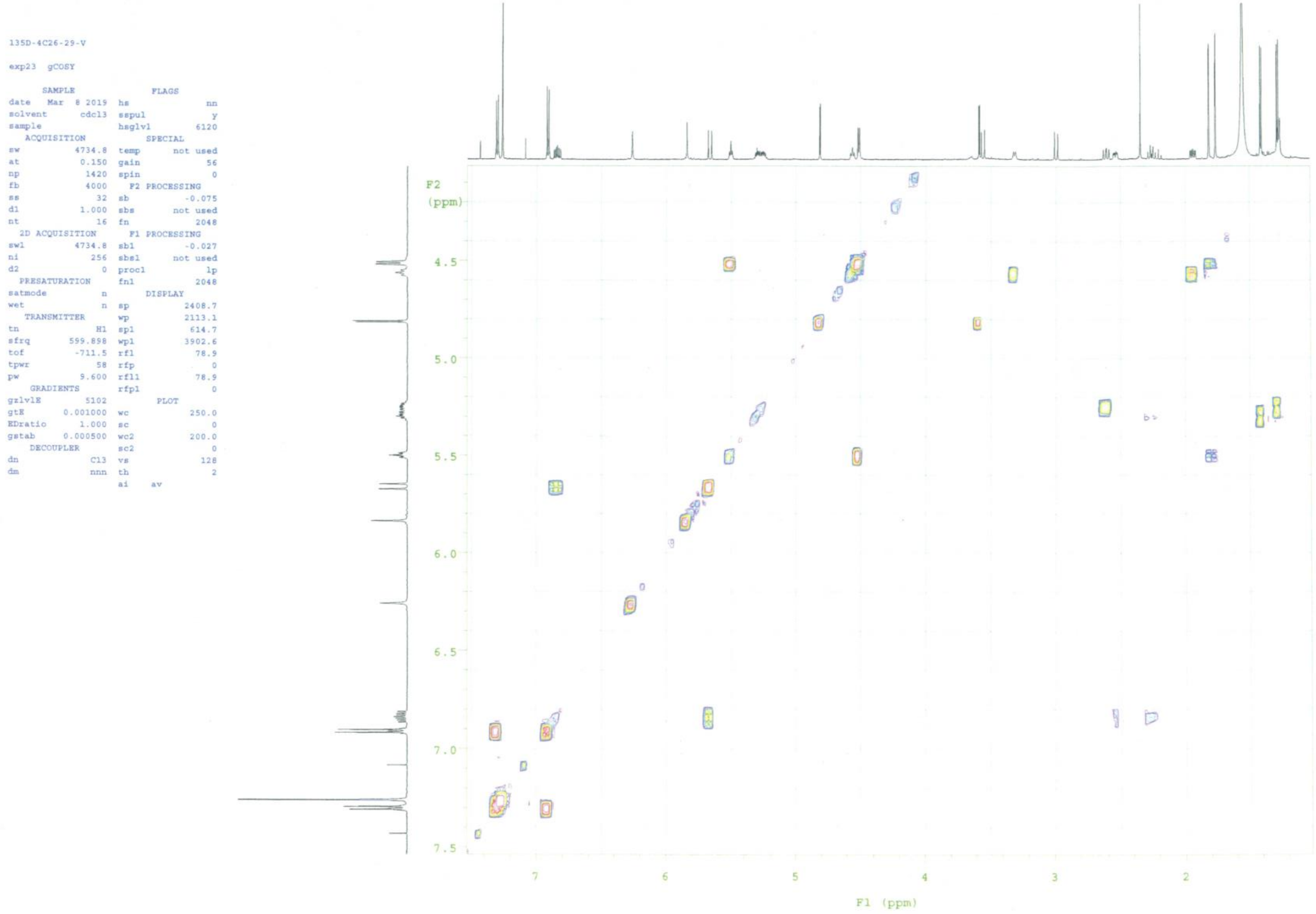
135D-4C26-29-V
exp23 gCOSY
SAMPLE
date Mar 8 2019
solvent cdcl3
sample
ACQUISITION
sw 4734.8
at 0.150
np 1420
fb 4000
ss 32
d1 1.000
nt 16
2D ACQUISITION
sw1 4734.8
ni 256
d2 0
PRESATURATION
satmode n
wet n
TRANSMITTER
tn H1
sfrq 599.898
tof -711.5
tpwr 58
pw 9.600
GRADIENTS
gzlvlE 5102
gtE 0.001000
EDratio 1.000
gstab 0.000500
DECOUPLER
dn C13
dm nnn
FLAGS
hs nn
sspul y
hsglvl 6120
SPECIAL
temp not used
gain 56
spin 0
F2 PROCESSING
sb -0.075
sbs not used
fn 2048
F1 PROCESSING
sb1 -0.027
sbs1 not used
proc1 lp
fn1 2048
DISPLAY
sp 2408.7
wp 2113.1
sp1 614.7
wp1 3902.6
rfl 78.9
rfp 0
rfl1 78.9
rfp1 0
PLOT
wc 250.0
sc 0
wc2 200.0
sc2 0
vs 128
th 2
ai av
F2 (ppm)
4.5
5.0
5.5
6.0
6.5
7.0
7.5
7
6
5
4
3
2
F1 (ppm)

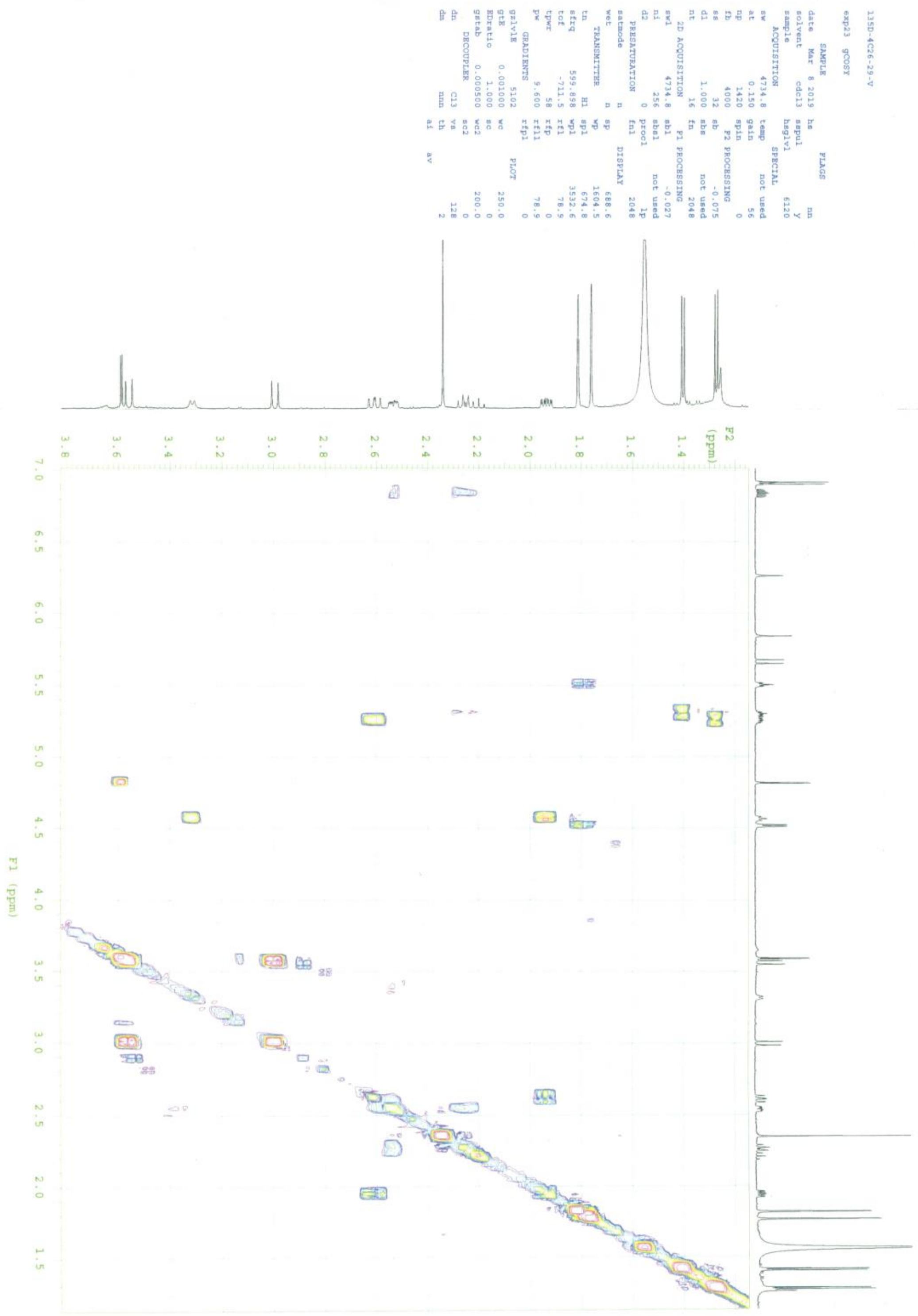
135D-4C26-29-V
exp23 gCOSY
F2 (ppm)
F1 (ppm)

Figure S5 NOESY of 2

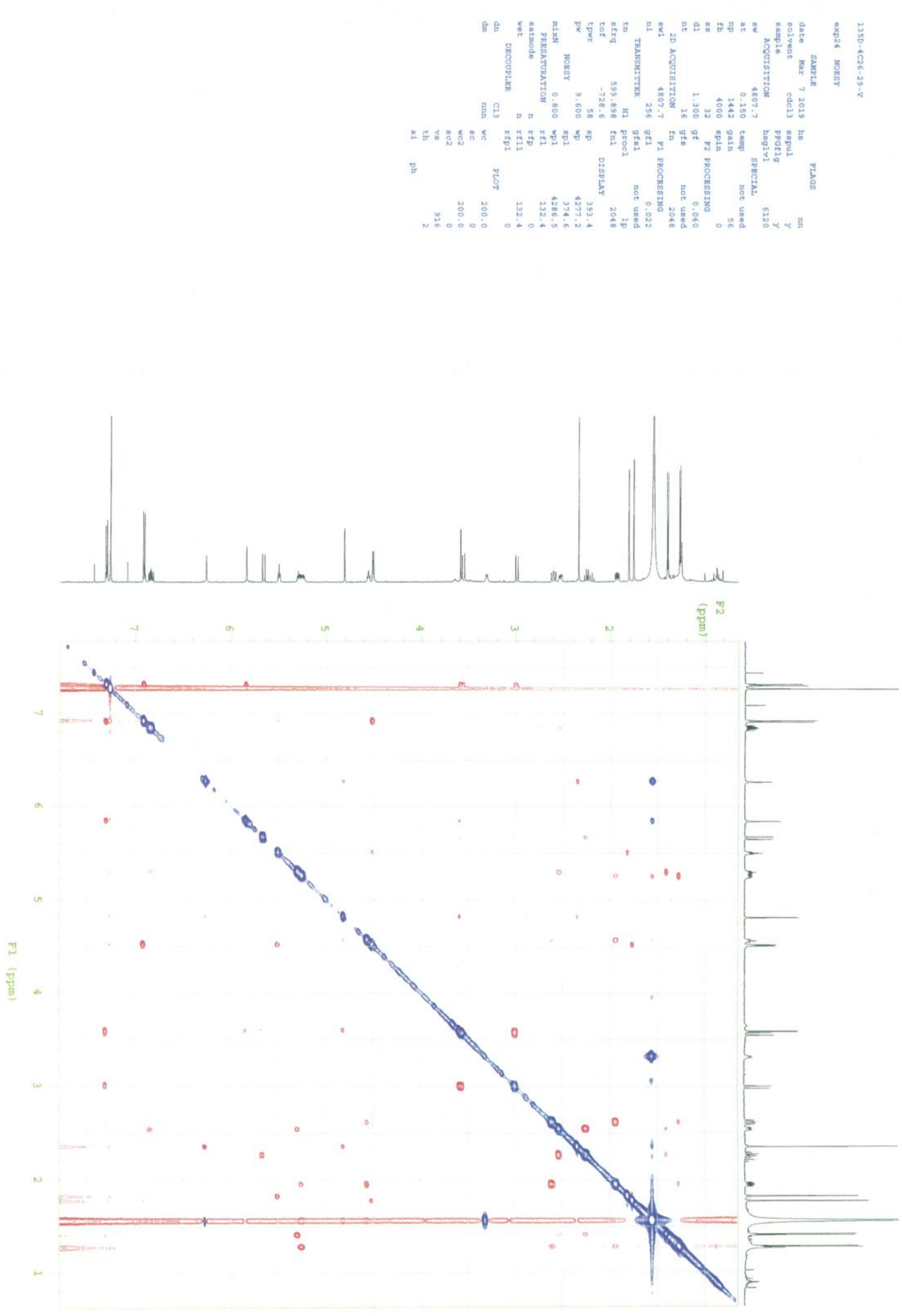

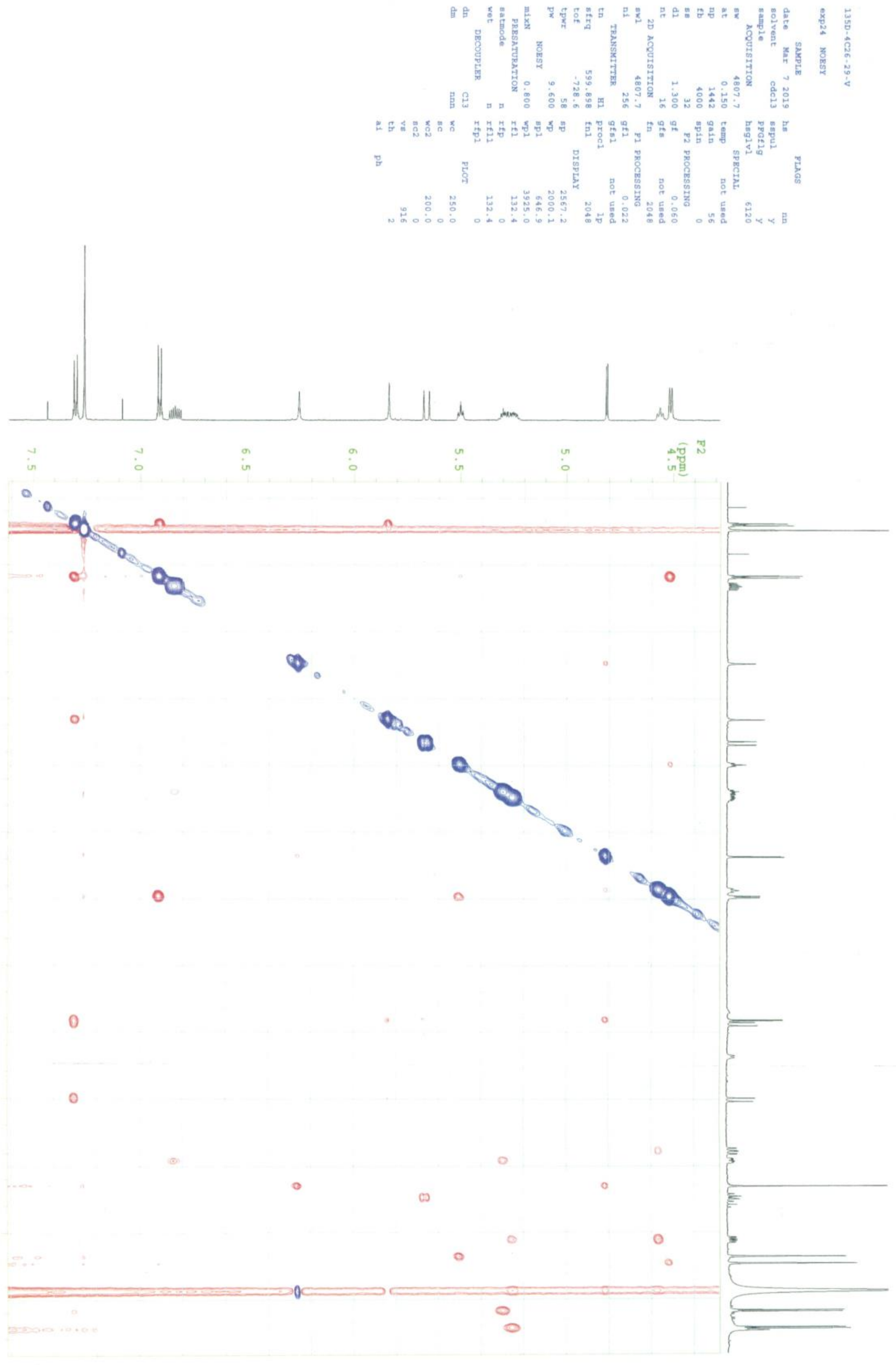
135D-4C26-29-V
exp24 NOESY
SAMPLE
date Mar 7 2019
solvent cdcl3
sample
ACQUISITION
sw 4807.7
at 0.150
np 1442
fb 4000
ss 32
d1 1.300
nt 16
2D ACQUISITION
sw1 4807.7
ni 256
TRANSMITTER
tn H1
sfrq 599.898
tof -728.6
tpwr 58
pw 9.600
NOESY
mixN 0.800
PRESATURATION
satmode n
wet n
DECOUPLER
dn C13
dm nnn
FLAGS
hs nn
sspul y
PFGflg y
hsglvl 6120
SPECIAL
temp not used
gain 56
spin 0
F2 PROCESSING
gf 0.060
gfs not used
fn 2048
F1 PROCESSING
gf1 0.022
gfs1 not used
proc1 lp
fn1 2048
DISPLAY
sp 2567.2
wp 2000.1
sp1 646.9
wp1 3925.0
rfl 132.4
rfp 0
rfl1 132.4
rfp1 0
PLOT
wc 250.0
sc 0
wc2 200.0
sc2 0
vs 916
th 2
ai ph
F2 (ppm)
F1 (ppm)

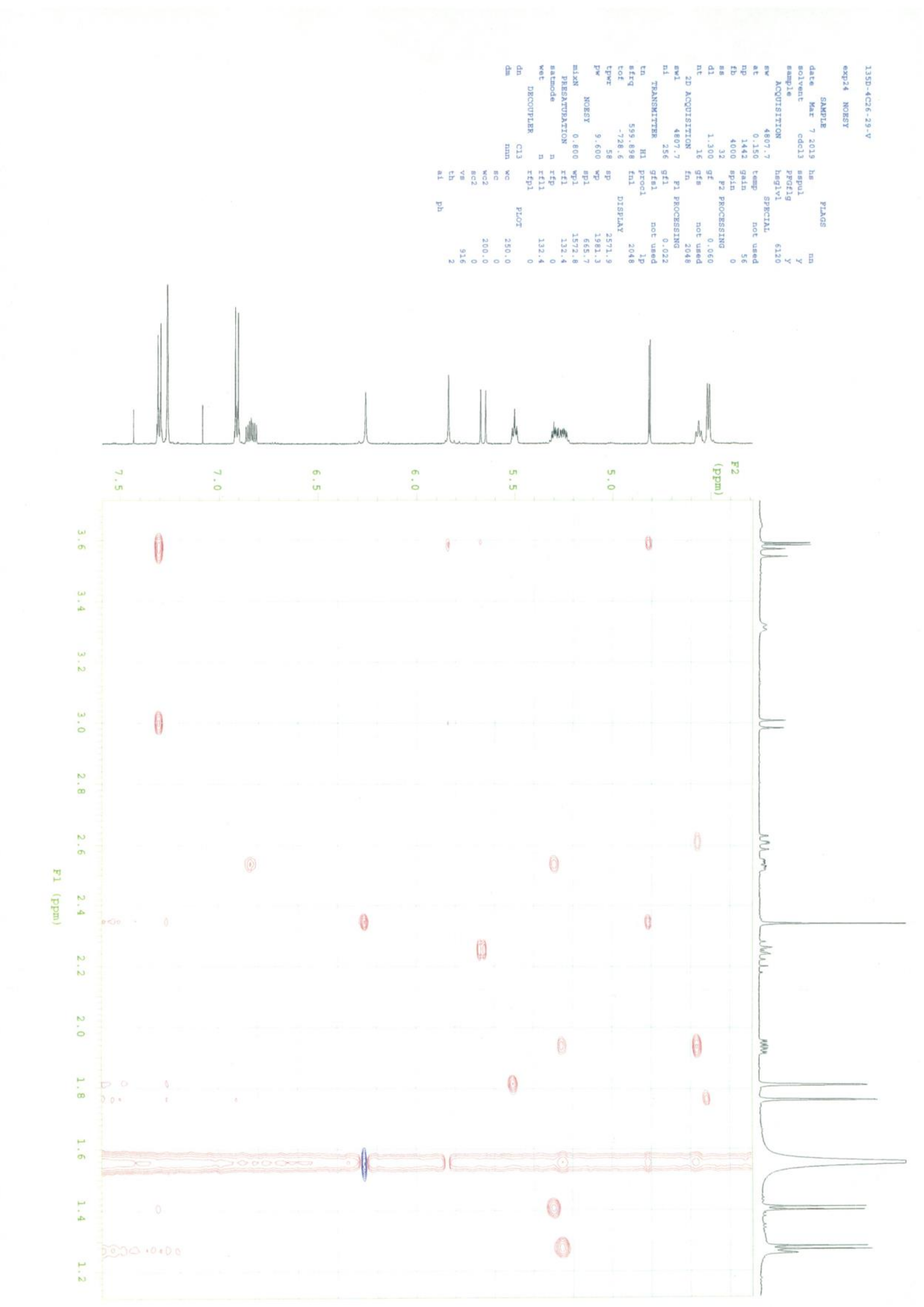
135D-4C26-29-V
exp24 NOESY
F2 (ppm)
F1 (ppm)

Figure S6 HMQC of 2

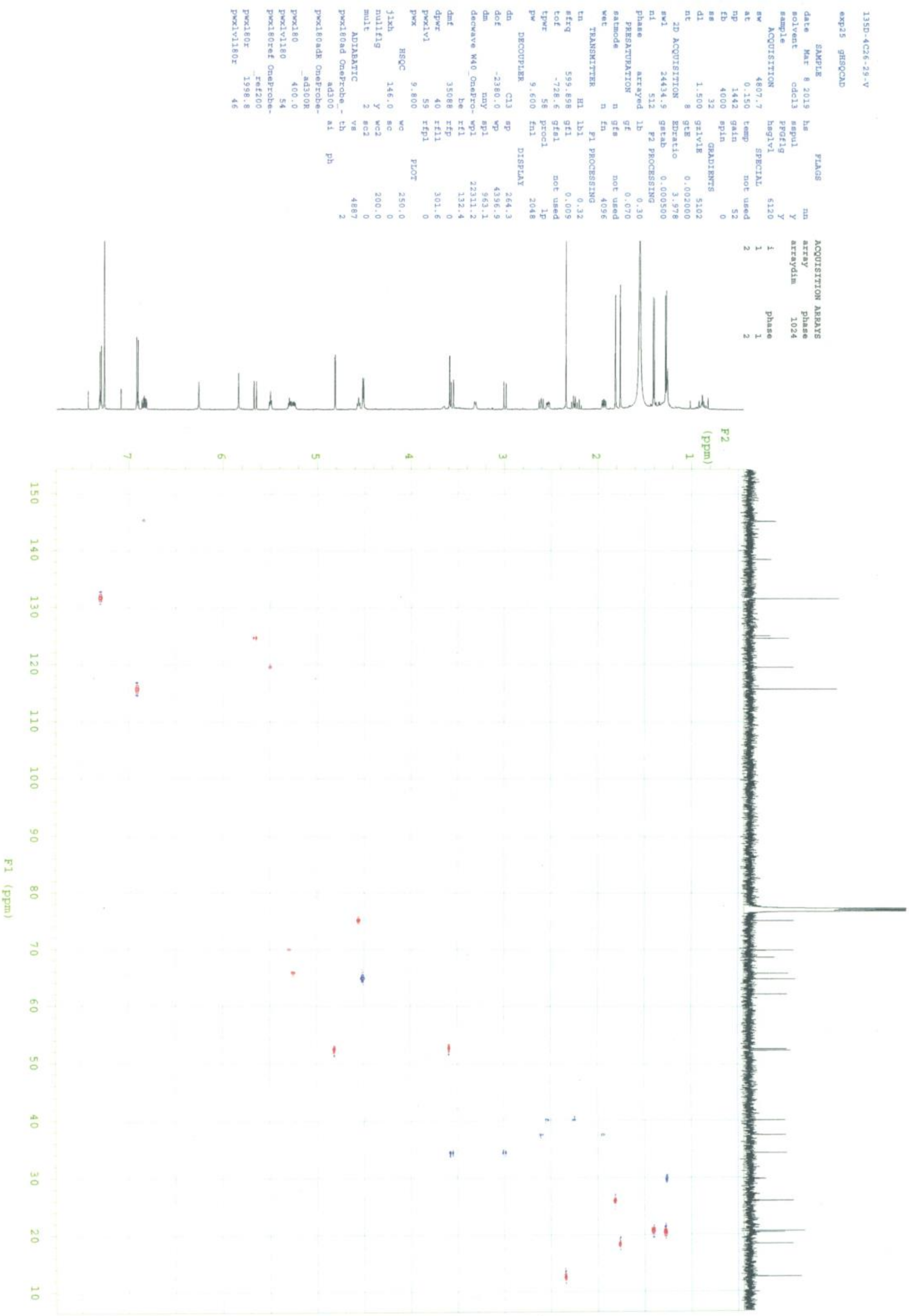

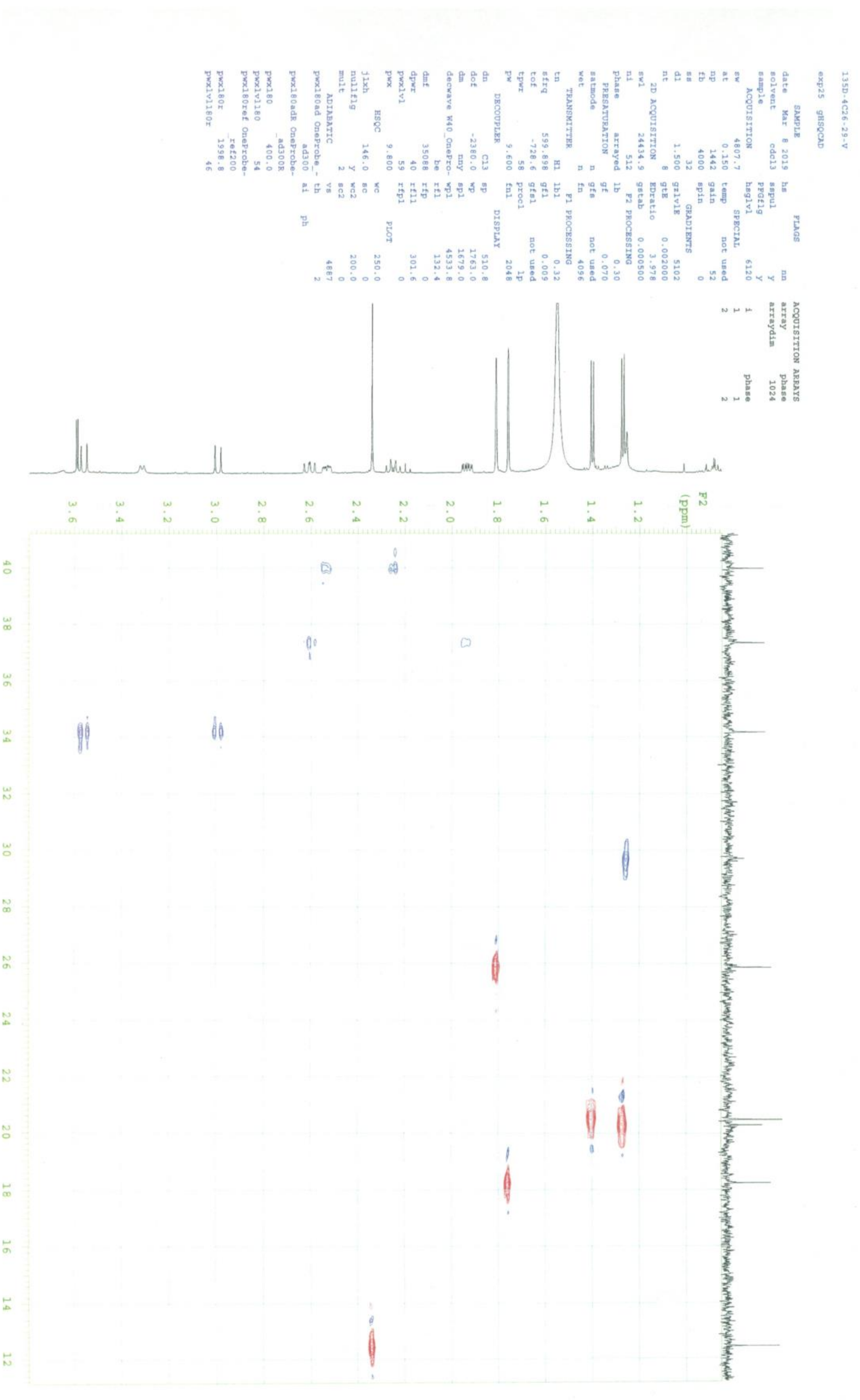
135D-4C26-29-V
exp25 gHSQCAD
F2 (ppm)
F1 (ppm)

Figure S7 HMBC of 2

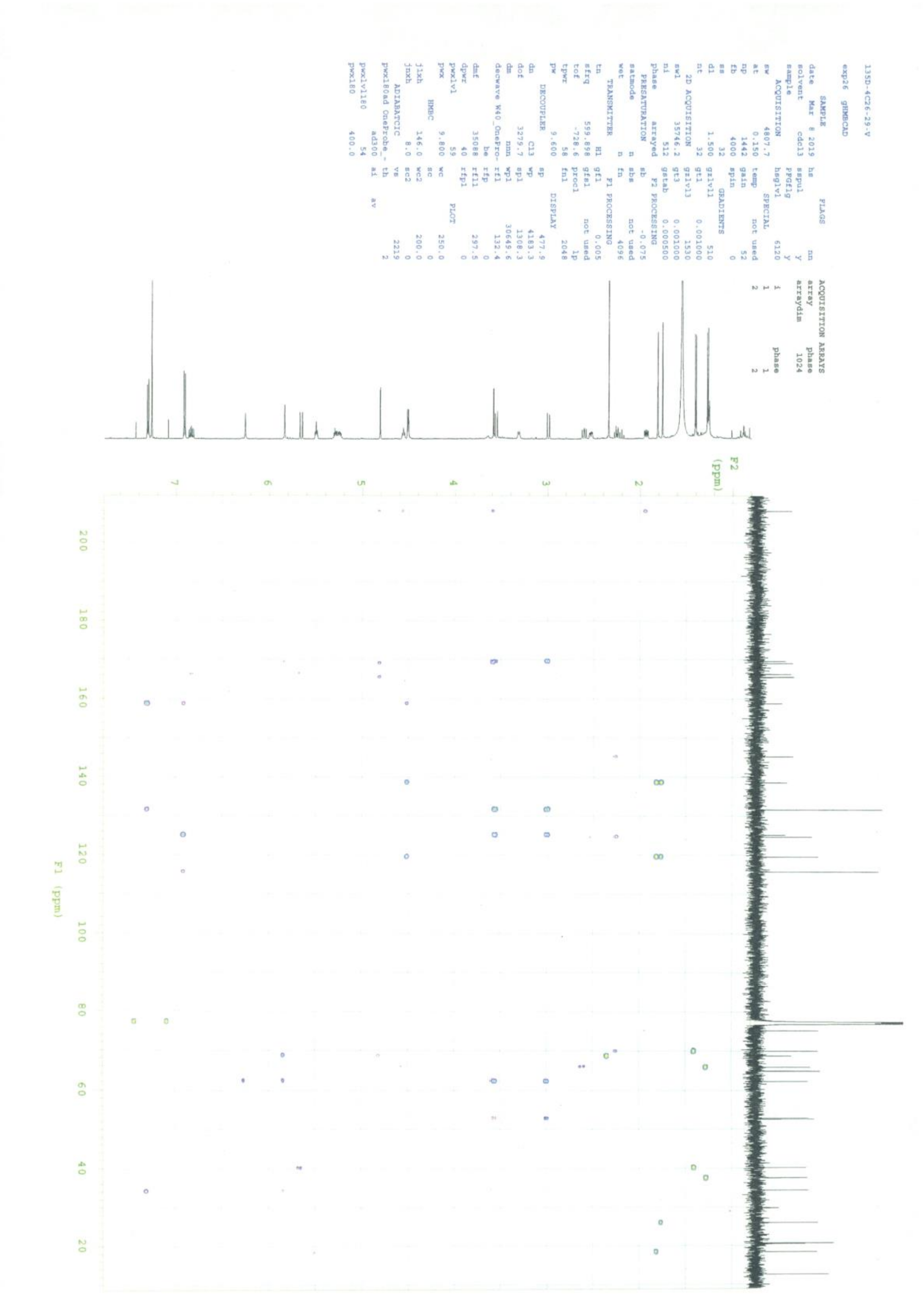

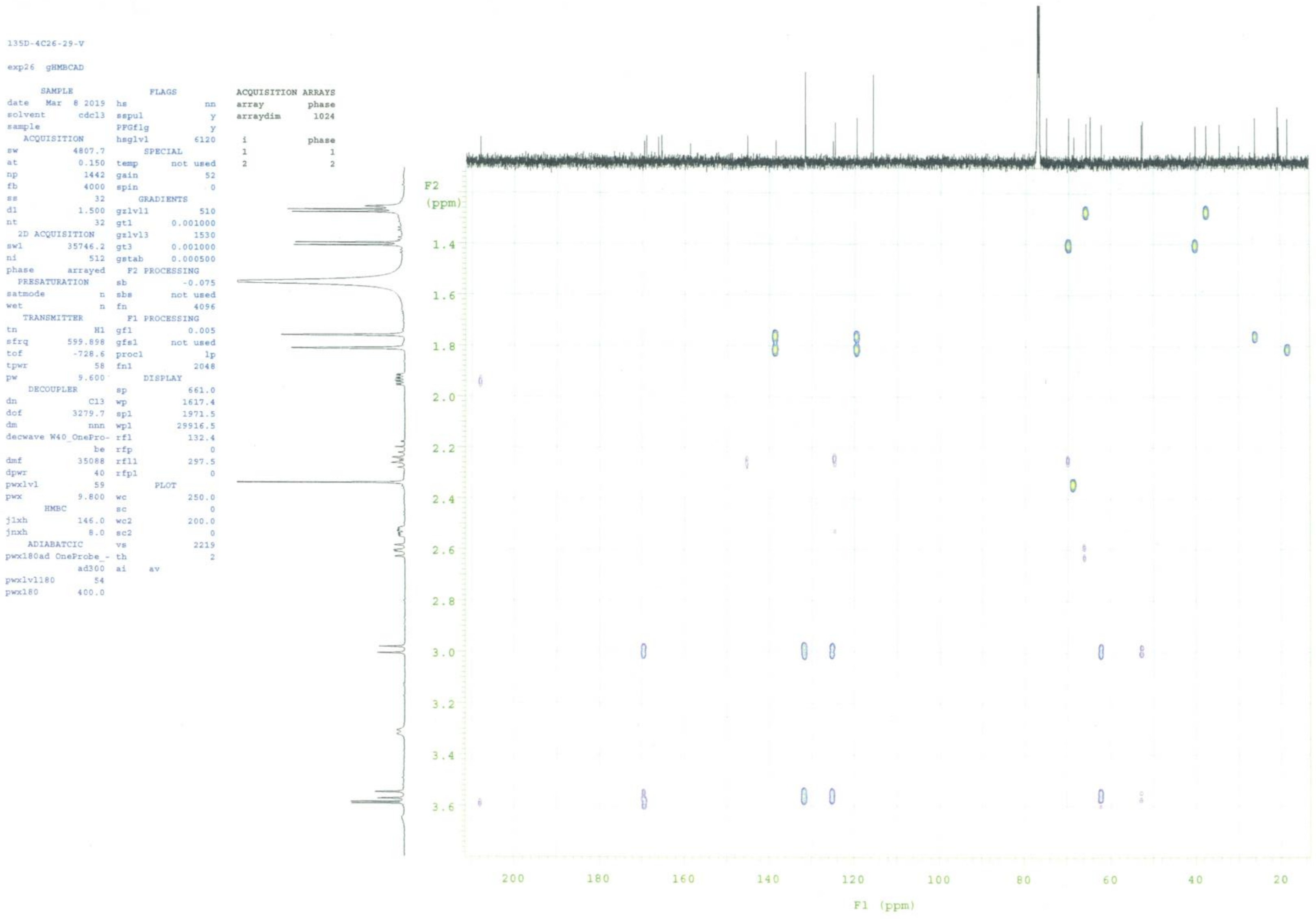
135D-4C26-29-V
exp26 gHMBCAD
SAMPLE
date Mar 8 2019
solvent cdcl3
sample
ACQUISITION
sw 4807.7
at 0.150
np 1442
fb 4000
ss 32
d1 1.500
nt 32
2D ACQUISITION
sw1 35746.2
ni 512
phase arrayed
PRESATURATION
satmode n
wet n
TRANSMITTER
tn H1
sfrq 599.898
tof -728.6
tpwr 58
pw 9.600
DECOUPLER
dn C13
dof 3279.7
dm nnn
decwave W40_OnePro-
be
dmf 35088
dpwr 40
pwxlvl 59
pwx 9.800
HMBC
j1xh 146.0
jnxh 8.0
ADIABATCIC
pwx180ad OneProbe_-
ad300
pwxlvl180 54
pwx180 400.0
FLAGS
hs nn
sspul y
PFGflg y
hsglvl 6120
SPECIAL
temp not used
gain 52
spin 0
GRADIENTS
gzlvl1 510
gt1 0.001000
gzlvl3 1530
gt3 0.001000
gstab 0.000500
F2 PROCESSING
sb -0.075
sbs not used
fn 4096
F1 PROCESSING
gf1 0.005
gfs1 not used
proc1 lp
fn1 2048
DISPLAY
sp 661.0
wp 1617.4
sp1 1971.5
wp1 29916.5
rfl 132.4
rfp 0
rfl1 297.5
rfp1 0
PLOT
wc 250.0
sc 0
wc2 200.0
sc2 0
vs 2219
th 2
ai av
ACQUISITION ARRAYS
array phase
arraydim 1024
i phase
1 1
2 2
F2 (ppm)
1.4
1.6
1.8
2.0
2.2
2.4
2.6
2.8
3.0
3.2
3.4
3.6
200
180
160
140
120
100
80
60
40
20
F1 (ppm)

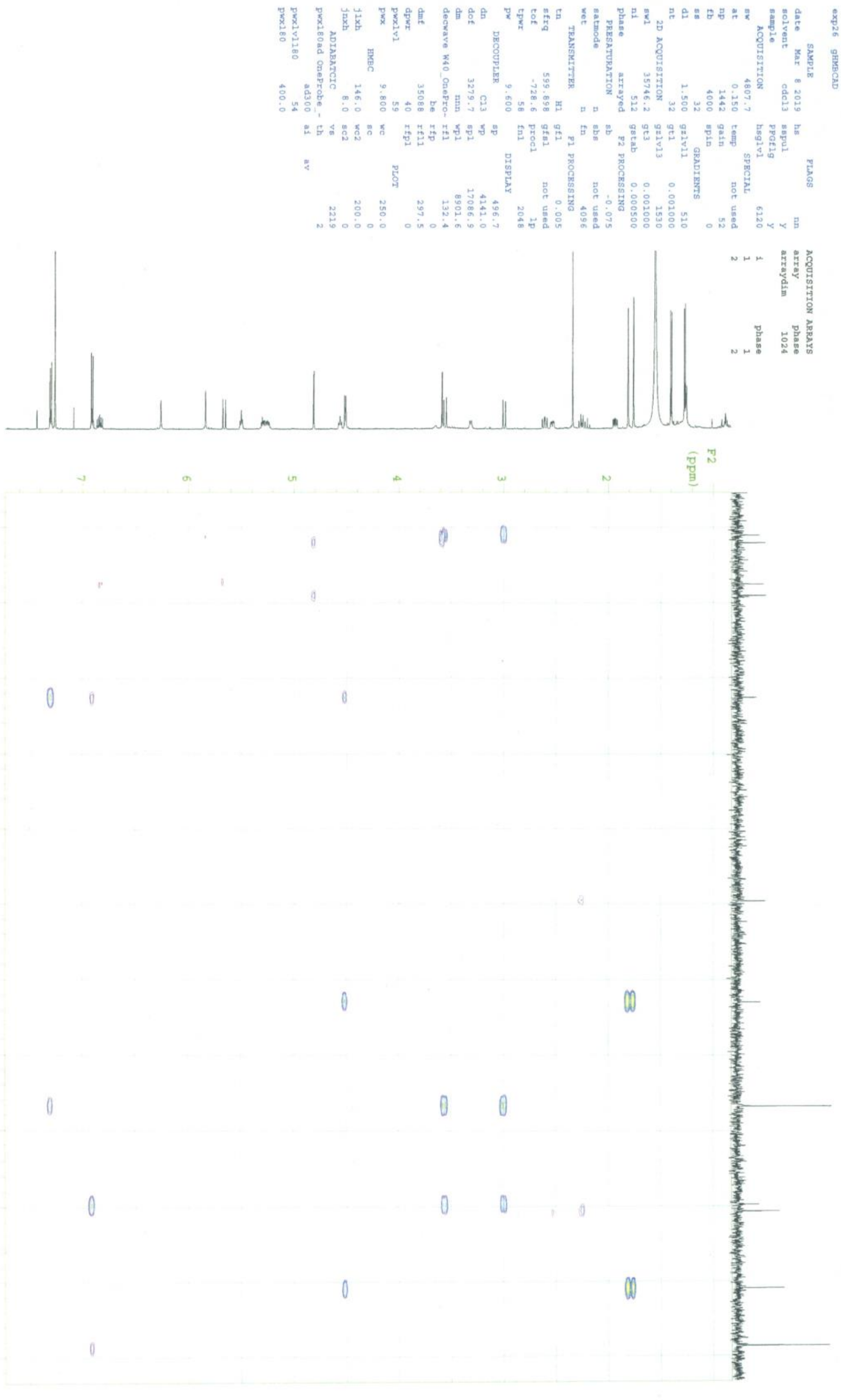
135D-4C26-29-V
exp26 gHMBCAD
SAMPLE
date Mar 8 2019
solvent cdcl3
sample
ACQUISITION
sw 4807.7
at 0.150
np 1442
fb 4000
ss 32
d1 1.500
nt 32
2D ACQUISITION
sw1 35746.2
ni 512
phase arrayed
PRESATURATION
satmode n
wet n
TRANSMITTER
tn H1
sfrq 599.898
tof -728.6
tpwr 58
pw 9.600
DECOUPLER
dn C13
dof 3279.7
dm nnn
decwave W40_OnePro-be
dmf 35088
dpwr 40
pwxlvl 59
pwx 9.800
HMBC
j1xh 146.0
jnxh 8.0
ADIABATCIC
pwx180ad OneProbe_-ad300
pwxlvl180 54
pwx180 400.0
FLAGS
hs nn
sspul y
PFGflg y
hsglvl 6120
SPECIAL
temp not used
gain 52
spin 0
GRADIENTS
gzlvl1 510
gt1 0.001000
gzlvl3 1530
gt3 0.001000
gstab 0.000500
F2 PROCESSING
sb -0.075
sbs not used
fn 4096
F1 PROCESSING
gf1 0.005
gfs1 not used
proc1 lp
fn1 2048
DISPLAY
sp 496.7
wp 4141.0
sp1 17086.9
wp1 8901.6
rfl 132.4
rfp 0
rfl1 297.5
rfp1 0
PLOT
wc 250.0
sc 0
wc2 200.0
sc2 0
vs 2219
th 2
ai av
ACQUISITION ARRAYS
array phase
arraydim 1024
i phase
1 1
2 2
F2 (ppm)
F1 (ppm)

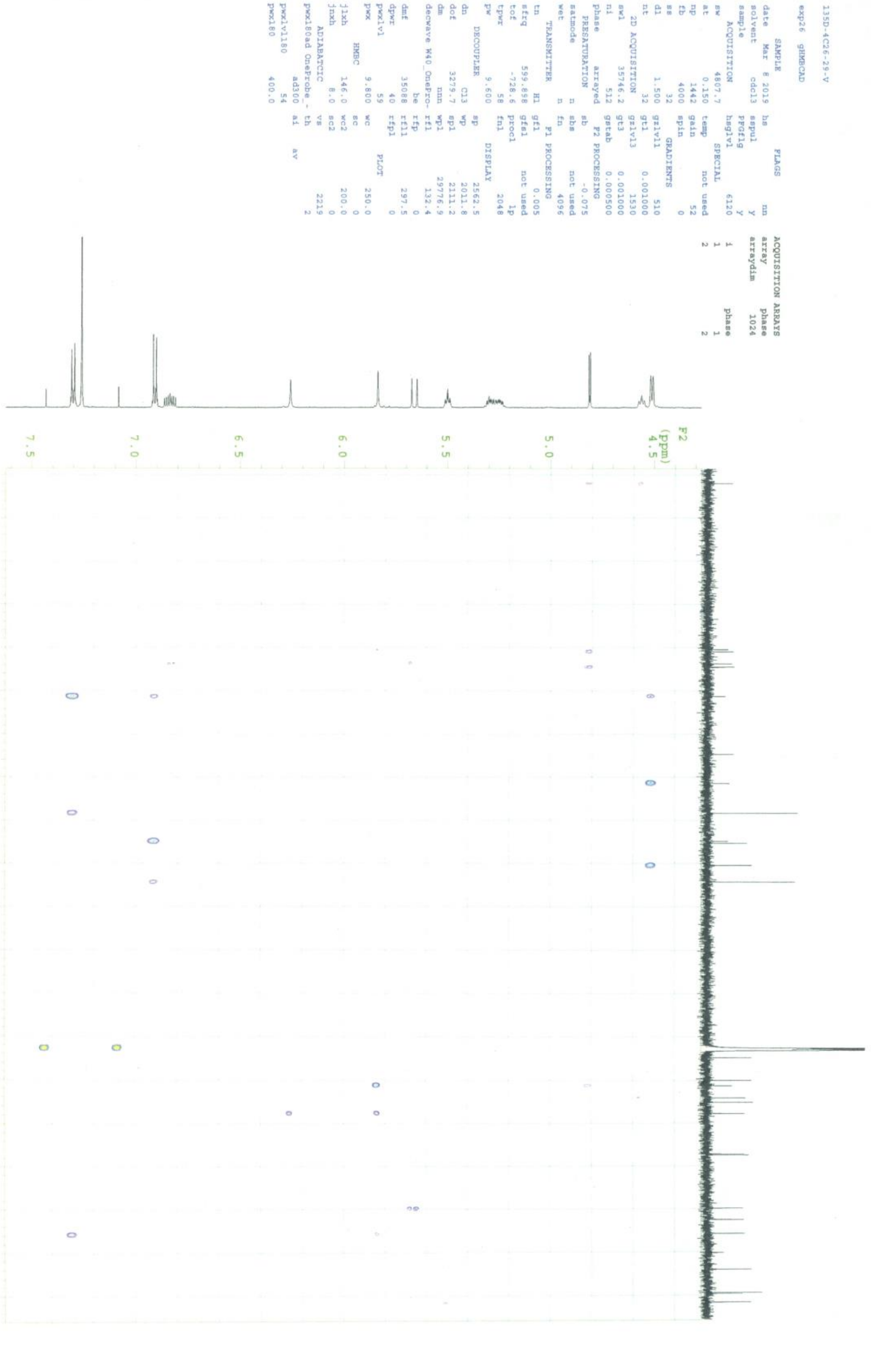

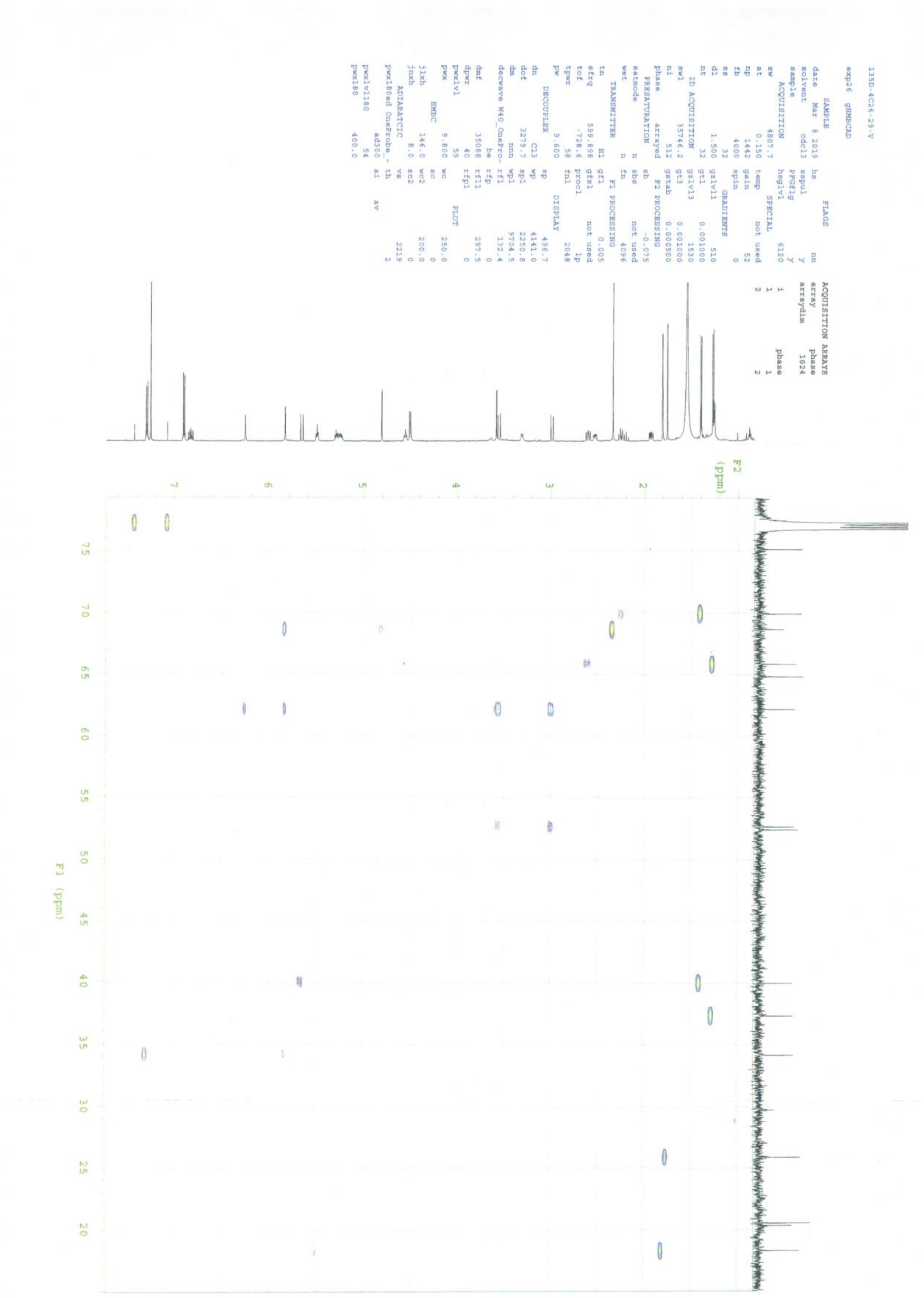
135D-4C26-29-V
exp26 gHMBCAD
F2 (ppm)
F1 (ppm)

Figure S8 FABMS of 2

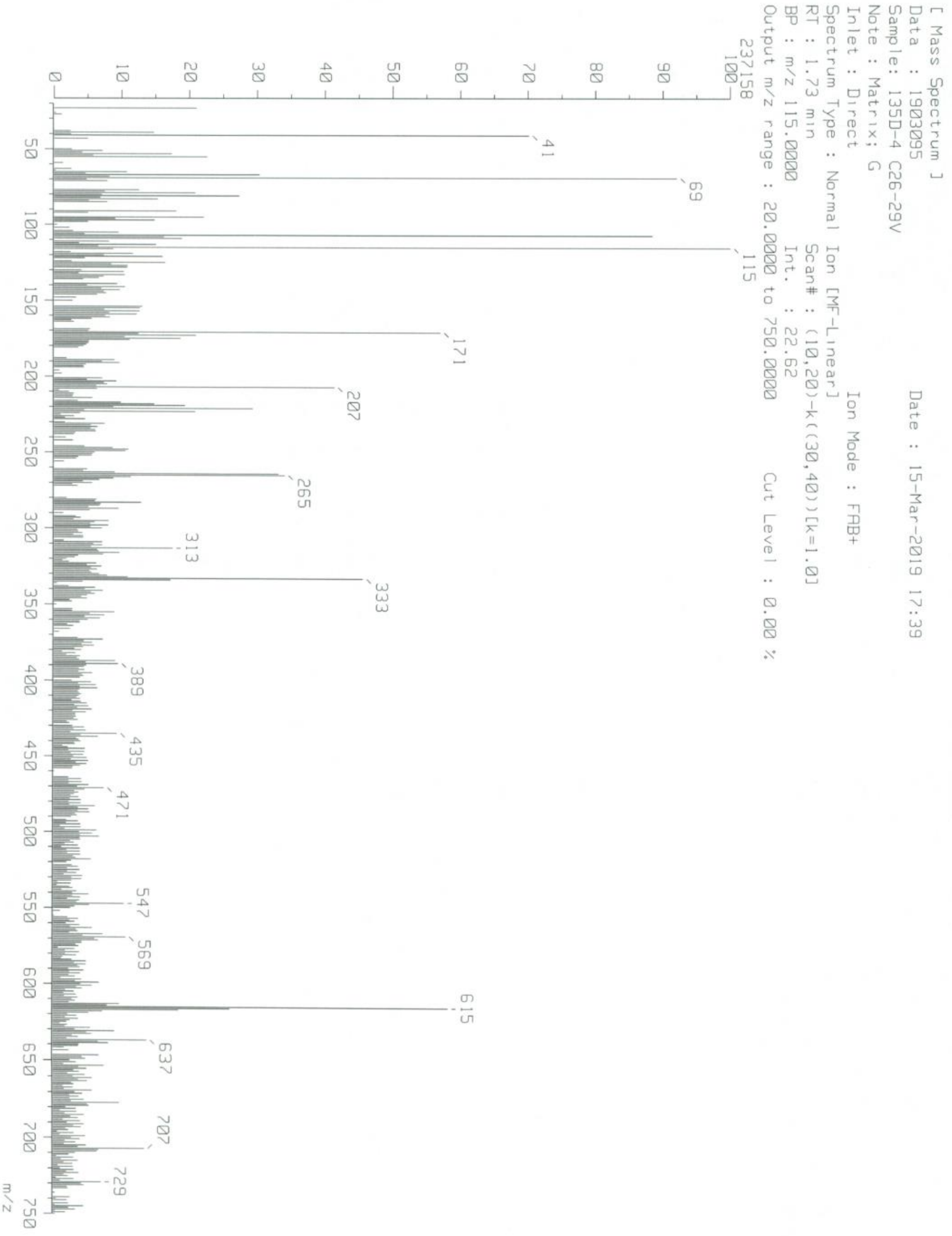

[Mass Spectrum]

Data : 1903095 Date : 15-Mar-2019 17:39
Sample: 135D-4 C26-29V
Note : Matrix; G
Inlet : Direct Ion Mode : FAB+
Spectrum Type : Normal Ion [MF-Linear]
RT : 1.73 min Scan# : (10,20)-k((30,40))[k=1.0]
BP : m/z 115.0000 Int. : 22.62
Output m/z range : 20.0000 to 750.0000 Cut Level : 10.00 %

m/z	Int.	Norm.
23.0000	4.75	21.00
39.0000	3.33	14.72
41.0000	15.86	70.10
53.0000	3.93	17.38
55.0000	5.10	22.56
65.0000	2.43	10.74
67.0000	6.86	30.32
69.0000	20.82	92.06
77.0000	2.84	12.56
79.0000	4.71	20.84
81.0000	6.17	27.30
83.0000	3.47	15.32
91.0000	4.08	18.03
95.0000	4.99	22.07
97.0000	3.36	14.85
107.0000	20.01	88.45
108.0000	3.66	16.18
109.0000	4.26	18.85
113.0000	3.40	15.03
115.0000	22.62	100.00
119.0000	2.63	11.65
121.0000	3.61	15.97
125.0000	3.71	16.42
127.0000	2.45	10.85
128.0000	2.44	10.78
131.0000	2.32	10.28
133.0000	2.36	10.43
141.0000	2.37	10.47
154.0000	2.94	13.02
155.0000	2.86	12.65
157.0000	2.86	12.64
159.0000	2.76	12.20
171.0000	12.90	57.02
172.0000	2.82	12.45
173.0000	4.74	20.96
174.0000	2.35	10.38
175.0000	4.22	18.66
176.0000	2.52	11.15
207.0000	9.35	41.34
218.0000	3.34	14.77
219.0000	4.37	19.31
221.0000	6.63	29.32
223.0000	4.71	20.82
248.0000	2.48	10.96
249.0000	2.38	10.52
264.0000	7.49	33.11
265.0000	7.69	34.02
266.0000	2.57	11.34
283.0000	2.91	12.85
313.0000	3.96	17.51

m/z	Int.	Norm.
332.0000	2.45	10.85
333.0000	10.30	45.55
334.0000	3.88	17.15
547.0000	2.36	10.44
569.0000	2.42	10.69
615.0000	13.19	58.31
616.0000	5.90	26.10
617.0000	4.19	18.52
637.0000	3.13	13.84
707.0000	3.04	13.45

[Elemental Composition]

Page: 1

Data : 1904044

Date : 11-Apr-2019 18:01

Sample: 135D-4 C26-29 V

Note : Matrix; G

Inlet : Direct

Ion Mode : FAB+

RT : 6.47 min

Scan#: (50,55)

Elements : C 35/25, H 45/35, N 4/0, O 12/0, S 1/0

Mass Tolerance : 20ppm, 2mmu if m/z > 100

Unsaturation (U.S.) : -1.0 - 40.0

Observed m/z	Int%	Err[ppm / mmu]	U.S.	Composition
615.2378	100.0	+0.3 / +0.2	14.5	C 31 H 39 N 2 O 9 S

Figure S9 IR spectrum of 2

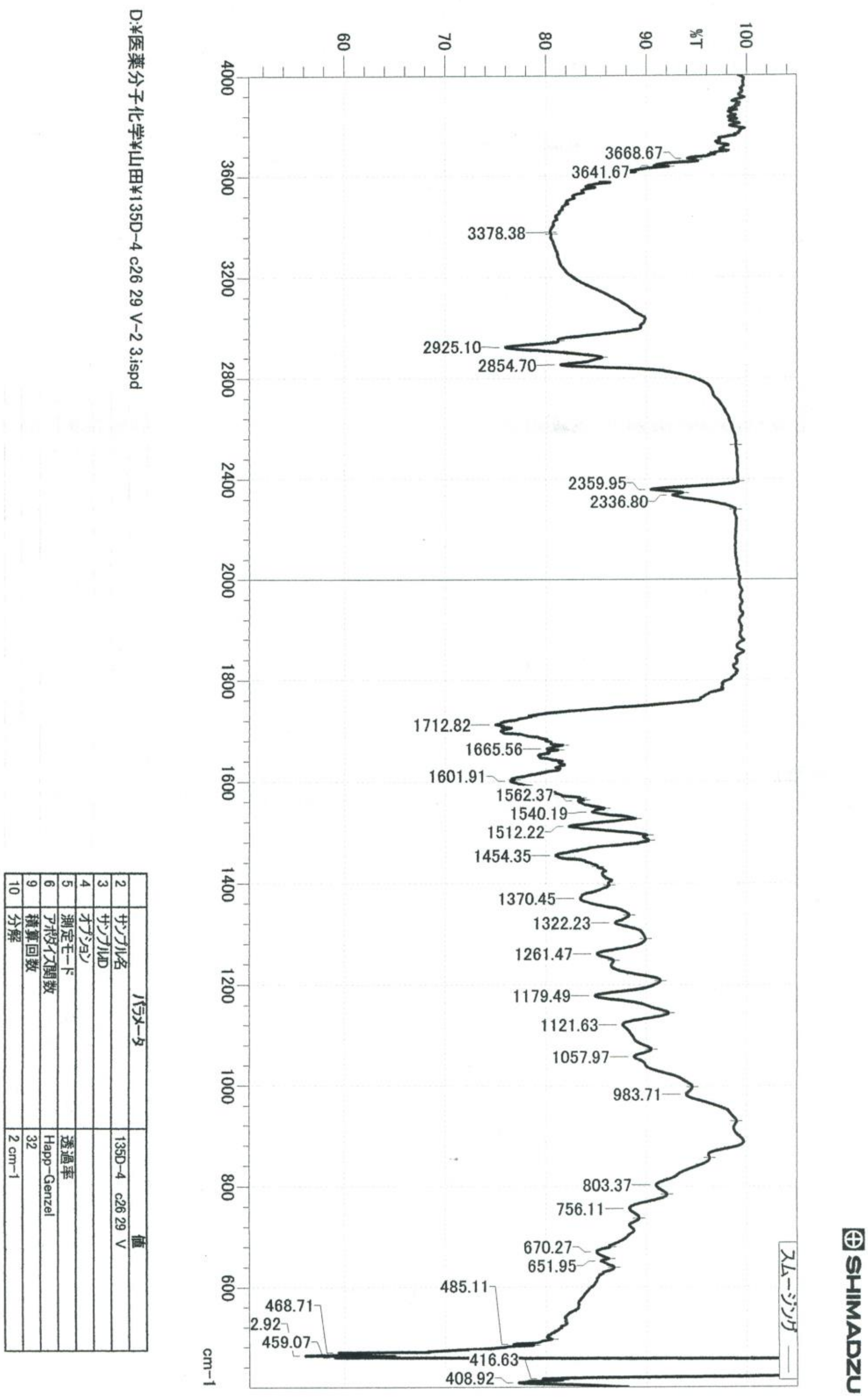

Figure S10 ^{1}H NMR spectrum of 3 in MeOH-d_4

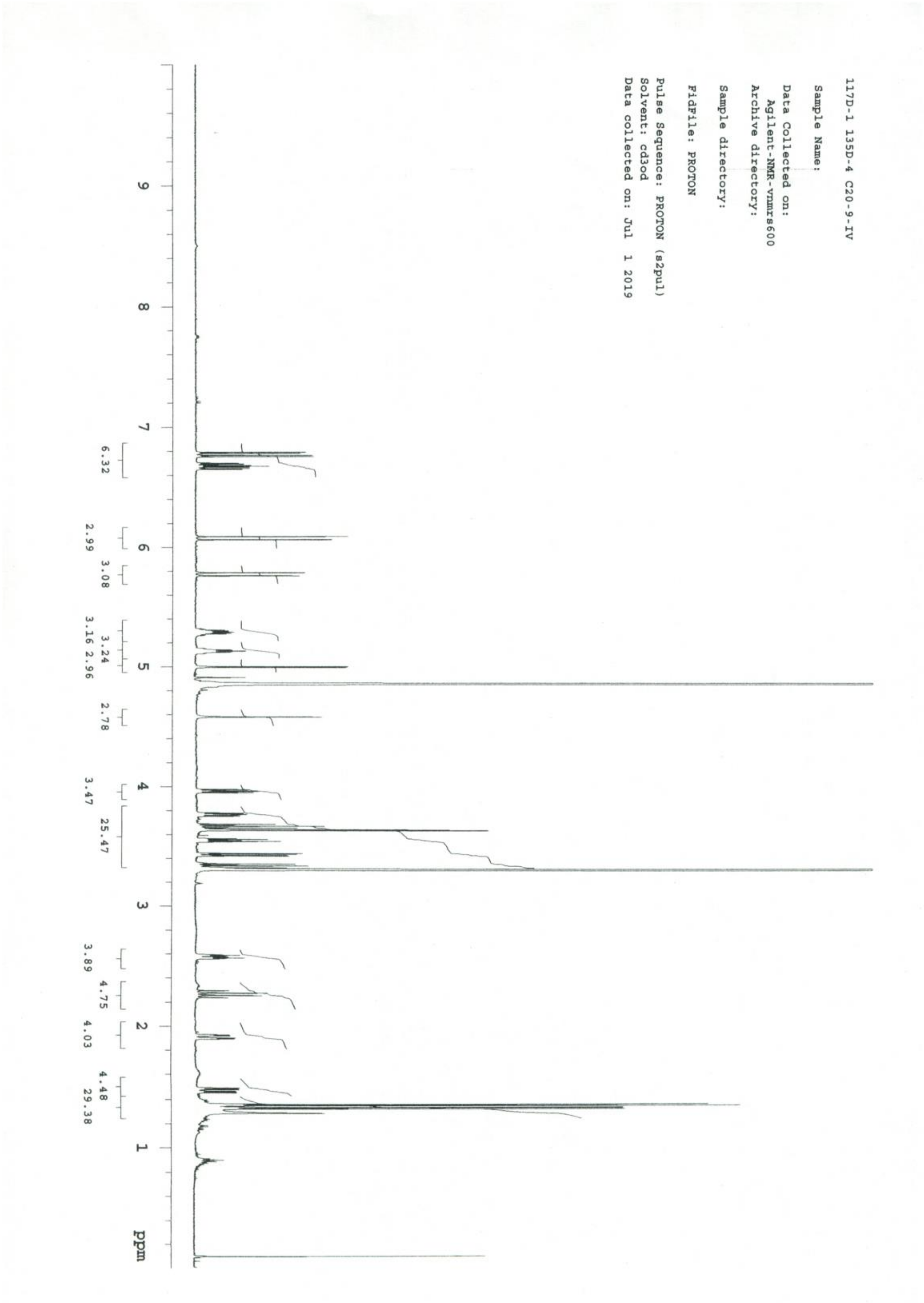

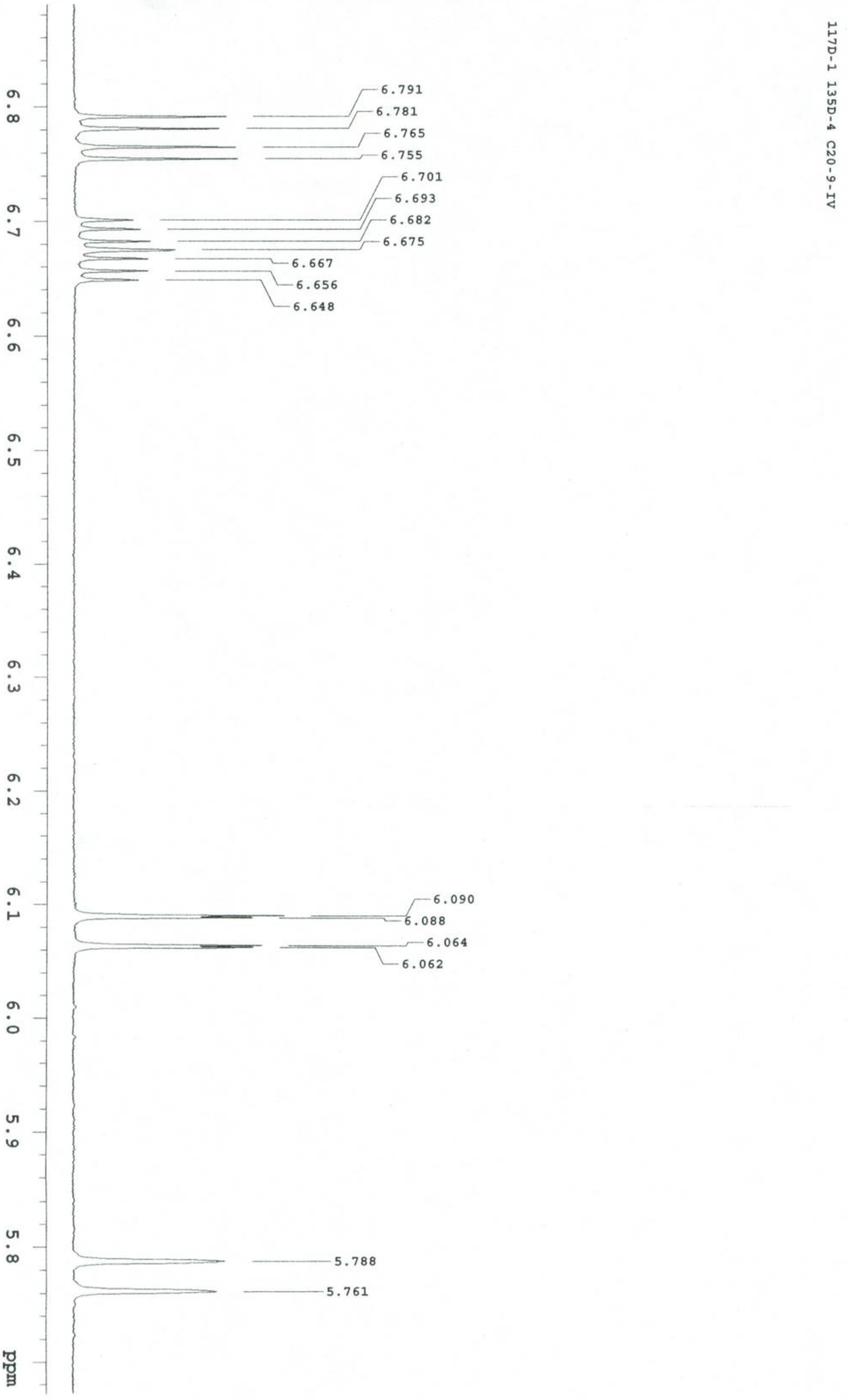
117D-1 135D-4 C20-9-IV
6.791
6.781
6.765
6.755
6.701
6.693
6.682
6.675
6.667
6.656
6.648
6.090
6.088
6.064
6.062
5.788
5.761
6.8
6.7
6.6
6.5
6.4
6.3
6.2
6.1
6.0
5.9
5.8
ppm

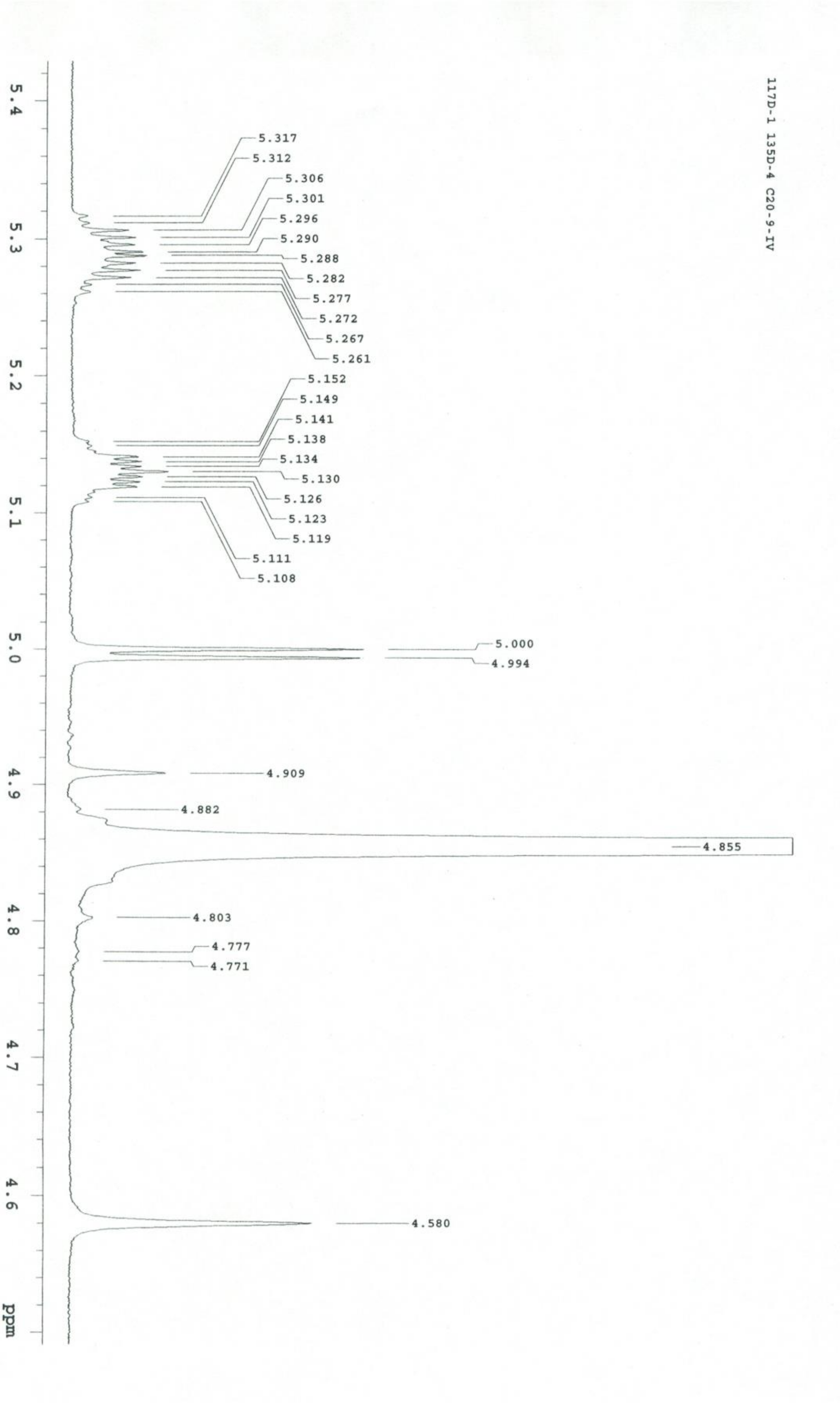
117D-1 135D-4 C20-9-IV
5.317
5.312
5.306
5.301
5.296
5.290
5.288
5.282
5.277
5.272
5.267
5.261
5.152
5.149
5.141
5.138
5.134
5.130
5.126
5.123
5.119
5.111
5.108
5.000
4.994
4.909
4.882
4.855
4.803
4.777
4.771
4.580
5.4
5.3
5.2
5.1
5.0
4.9
4.8
4.7
4.6
ppm

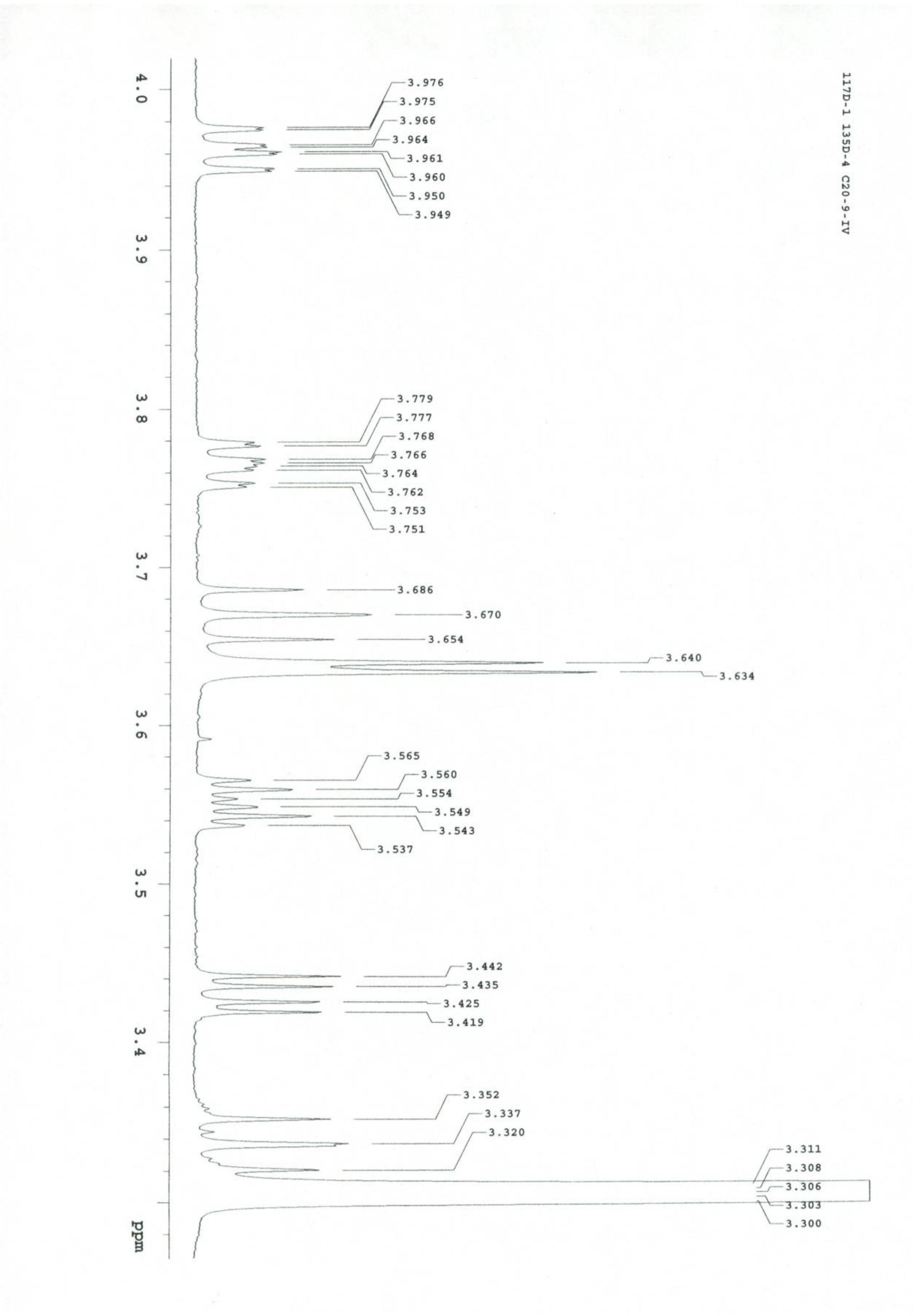
117D-1 135D-4 C20-9-IV
3.976
3.975
3.966
3.964
3.961
3.960
3.950
3.949
3.779
3.777
3.768
3.766
3.764
3.762
3.753
3.751
3.686
3.670
3.654
3.640
3.634
3.565
3.560
3.554
3.549
3.543
3.537
3.442
3.435
3.425
3.419
3.352
3.337
3.320
3.311
3.308
3.306
3.303
3.300
4.0
3.9
3.8
3.7
3.6
3.5
3.4
ppm

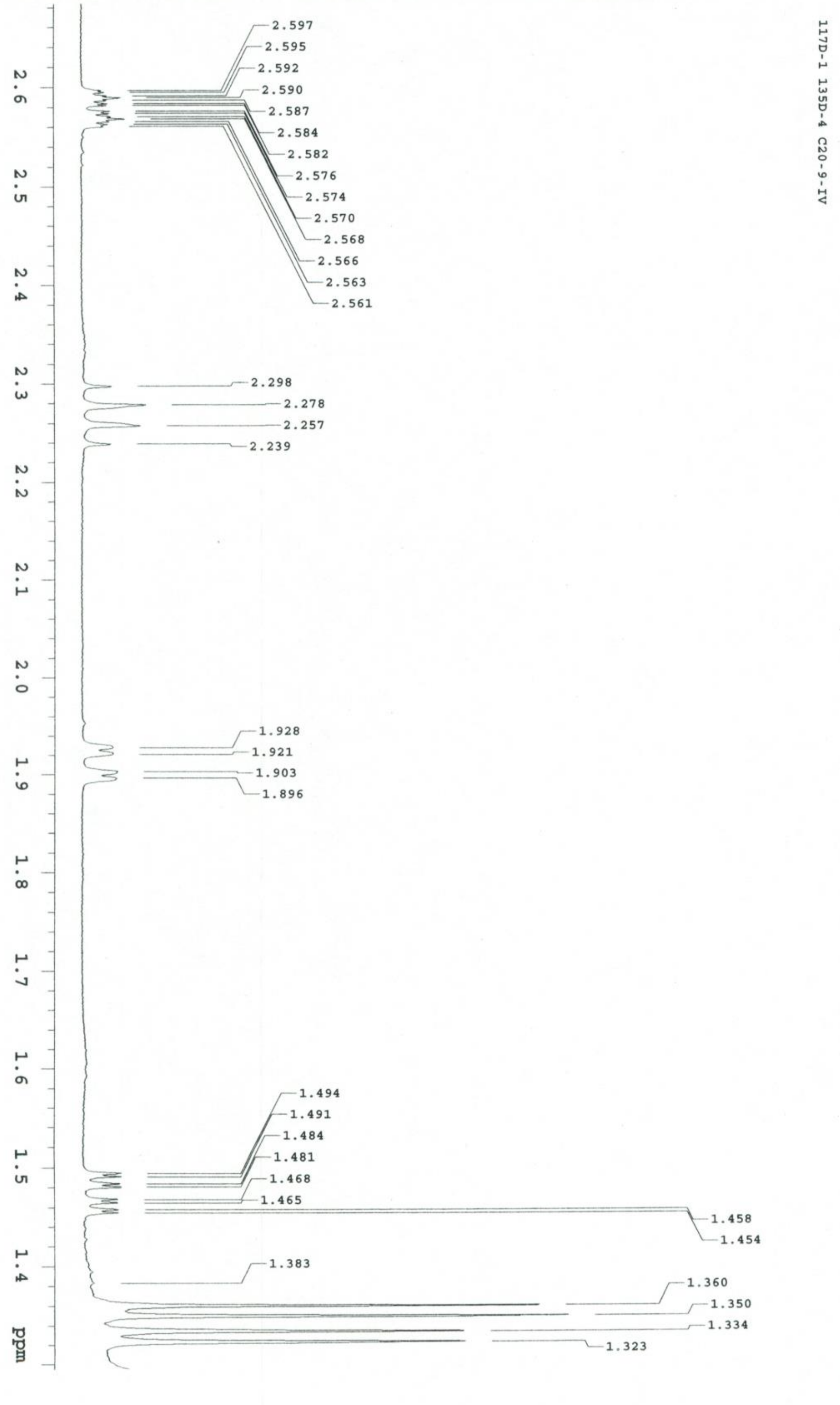
117D-1 135D-4 C20-9-IV
2.597
2.595
2.592
2.590
2.587
2.584
2.582
2.576
2.574
2.570
2.568
2.566
2.563
2.561
2.298
2.278
2.257
2.239
1.928
1.921
1.903
1.896
1.494
1.491
1.484
1.481
1.468
1.465
1.458
1.454
1.383
1.360
1.350
1.334
1.323
2.6
2.5
2.4
2.3
2.2
2.1
2.0
1.9
1.8
1.7
1.6
1.5
1.4
ppm

Figure S11 ^{13}C NMR spectrum of 3 in MeOH-d_4

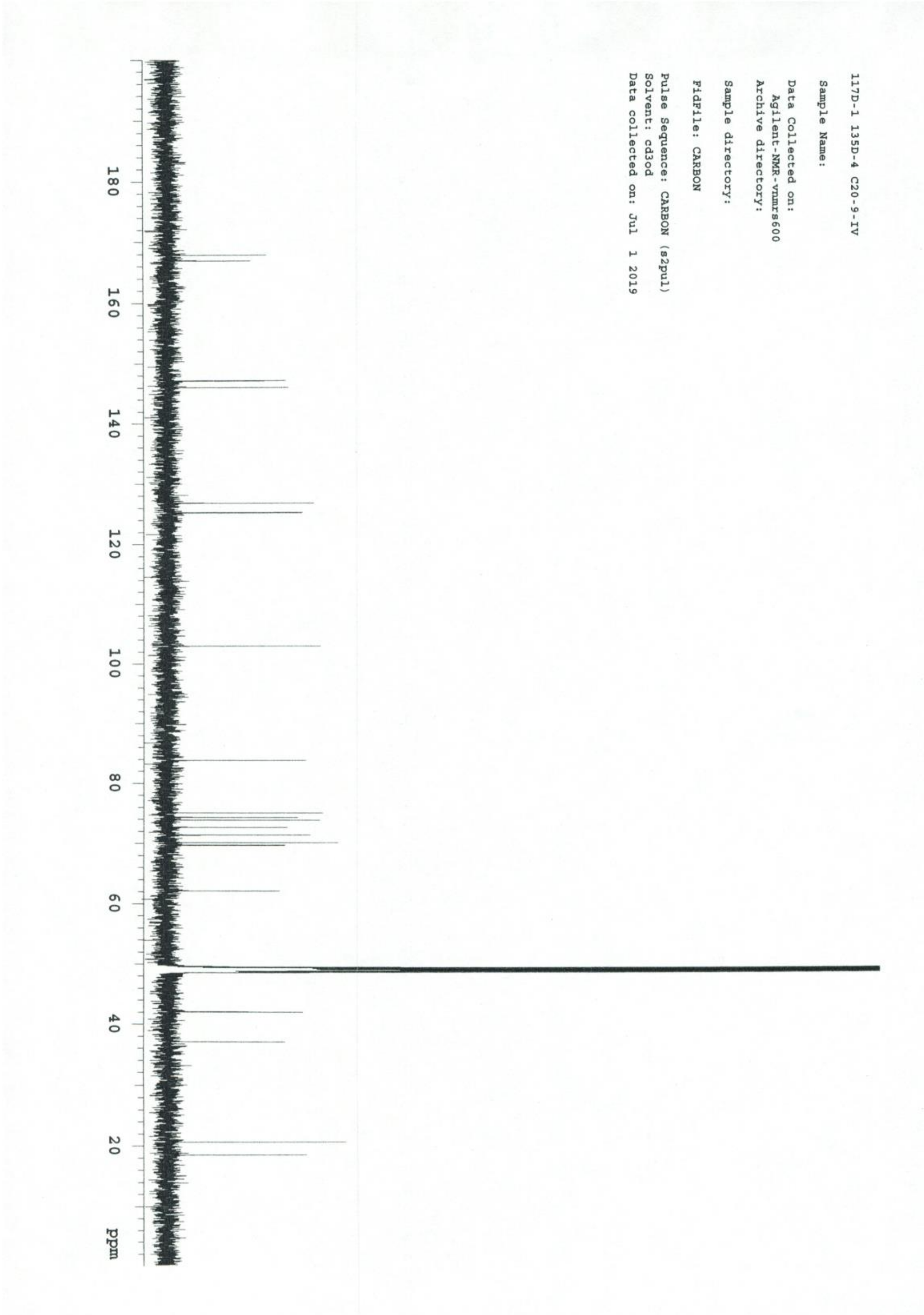

117D-1 135D-4 C20-9-IV

INDEX	FREQUENCY	PPM	HEIGHT
1	25316.9	167.835	21.7
2	25174.7	166.892	18.1
3	22196.9	147.151	25.7
4	22029.3	146.040	26.3
5	19127.8	126.805	31.9
6	18888.5	125.219	29.4
7	15528.1	102.941	33.4
8	12644.0	83.821	30.2
9	11323.9	75.070	33.9
10	11211.7	74.327	28.4
11	11141.2	73.859	33.2
12	10958.6	72.648	26.1
13	10764.4	71.361	31.1
14	10574.8	70.104	37.2
15	10512.4	69.690	25.5
16	9370.3	62.119	24.4
17	7483.7	49.612	13.5
18	7462.9	49.474	665.9
19	7441.0	49.329	1572.9
20	7420.2	49.191	3779.8
21	7398.2	49.045	3878.7
22	7377.4	48.907	3692.4
23	7355.4	48.762	1810.0
24	7334.6	48.624	589.1
25	6342.8	42.049	29.5
26	5592.6	37.075	25.6
27	3108.4	20.607	38.9
28	2782.4	18.446	30.2

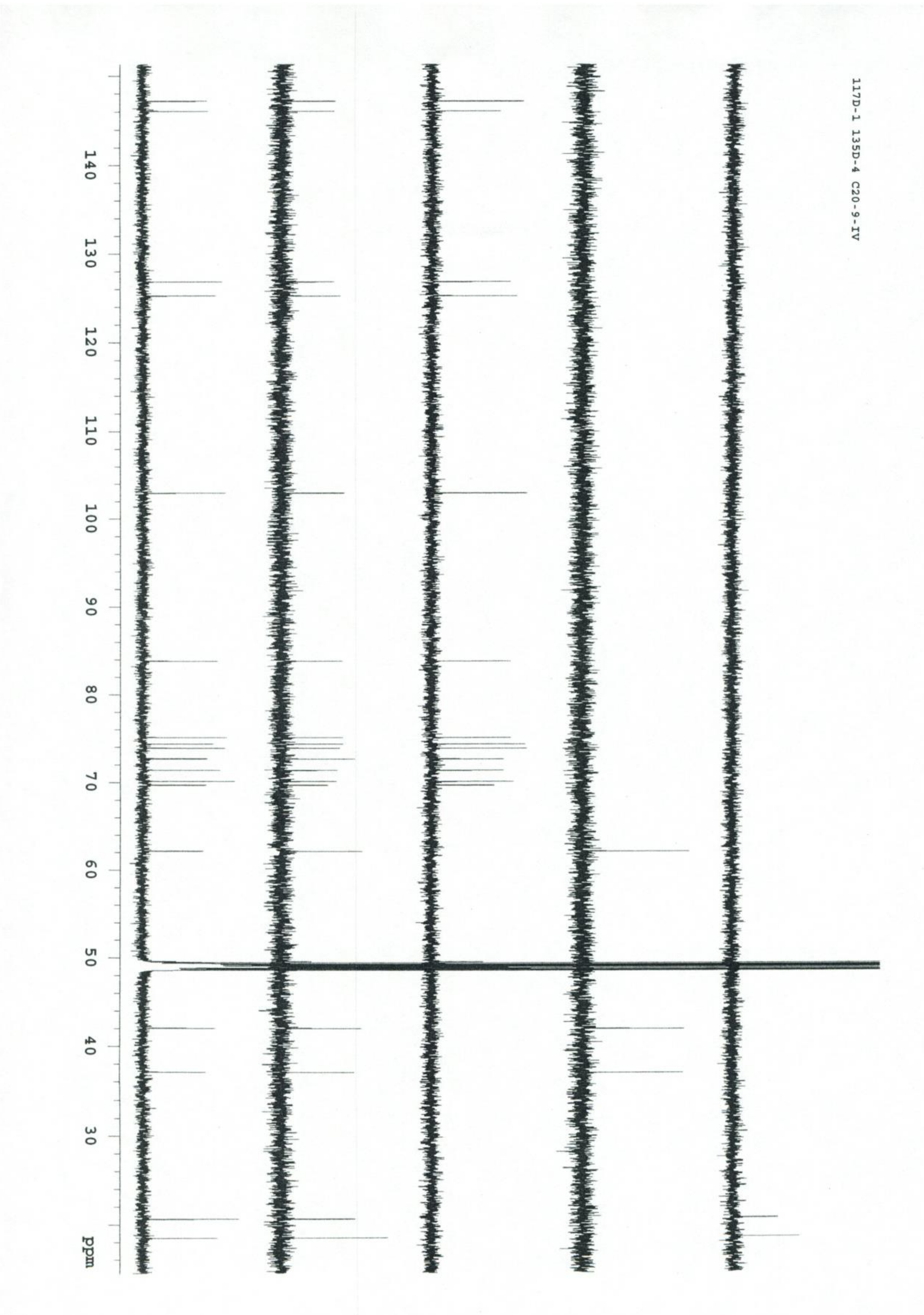
117D-1 135D-4 C20-9-IV
140
130
120
110
100
90
80
70
60
50
40
30
ppm

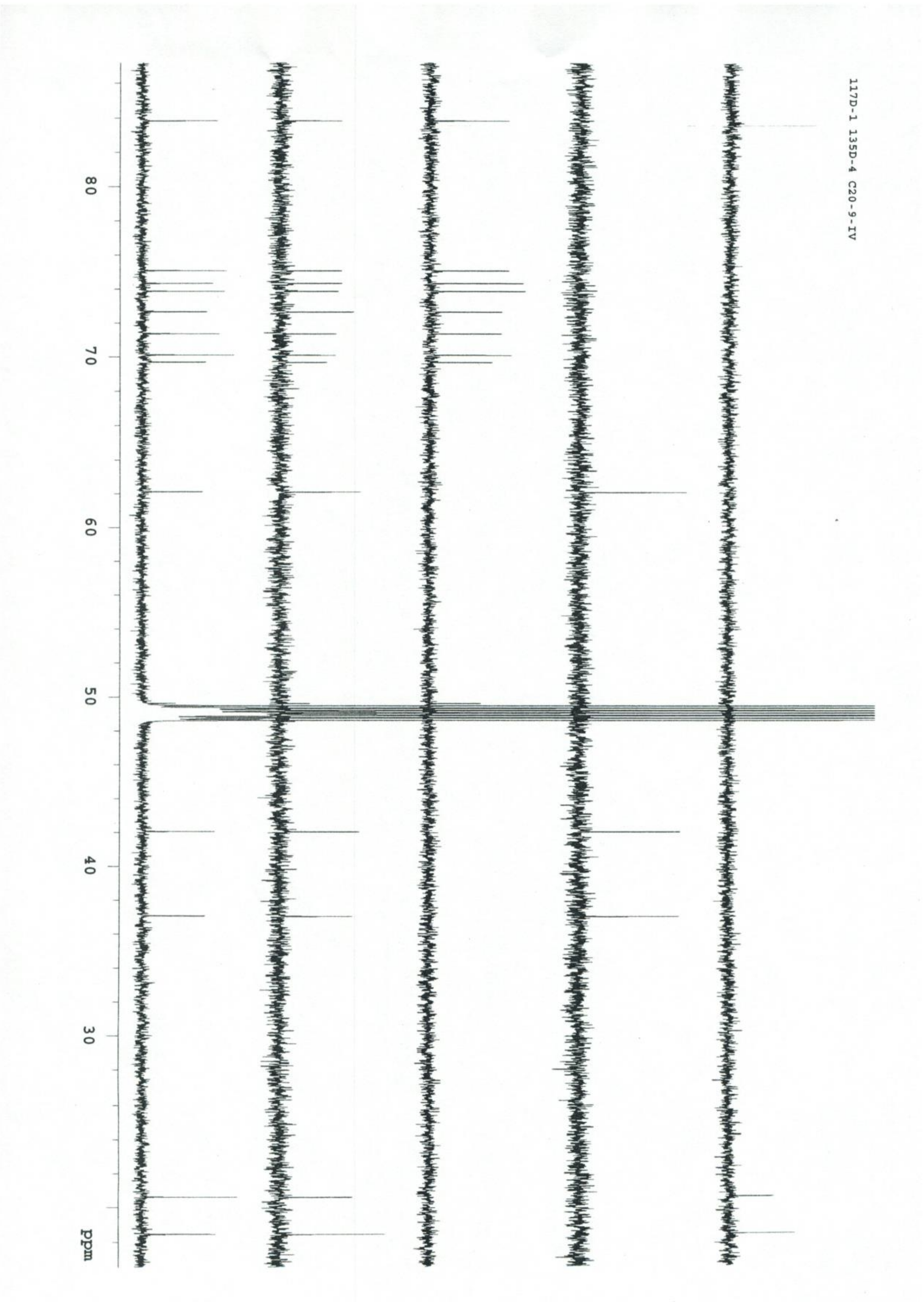
117D-1 135D-4 C20-9-IV
80
70
60
50
40
30
ppm

Figure S12 ^{1}H-^{1}H COSY of 3

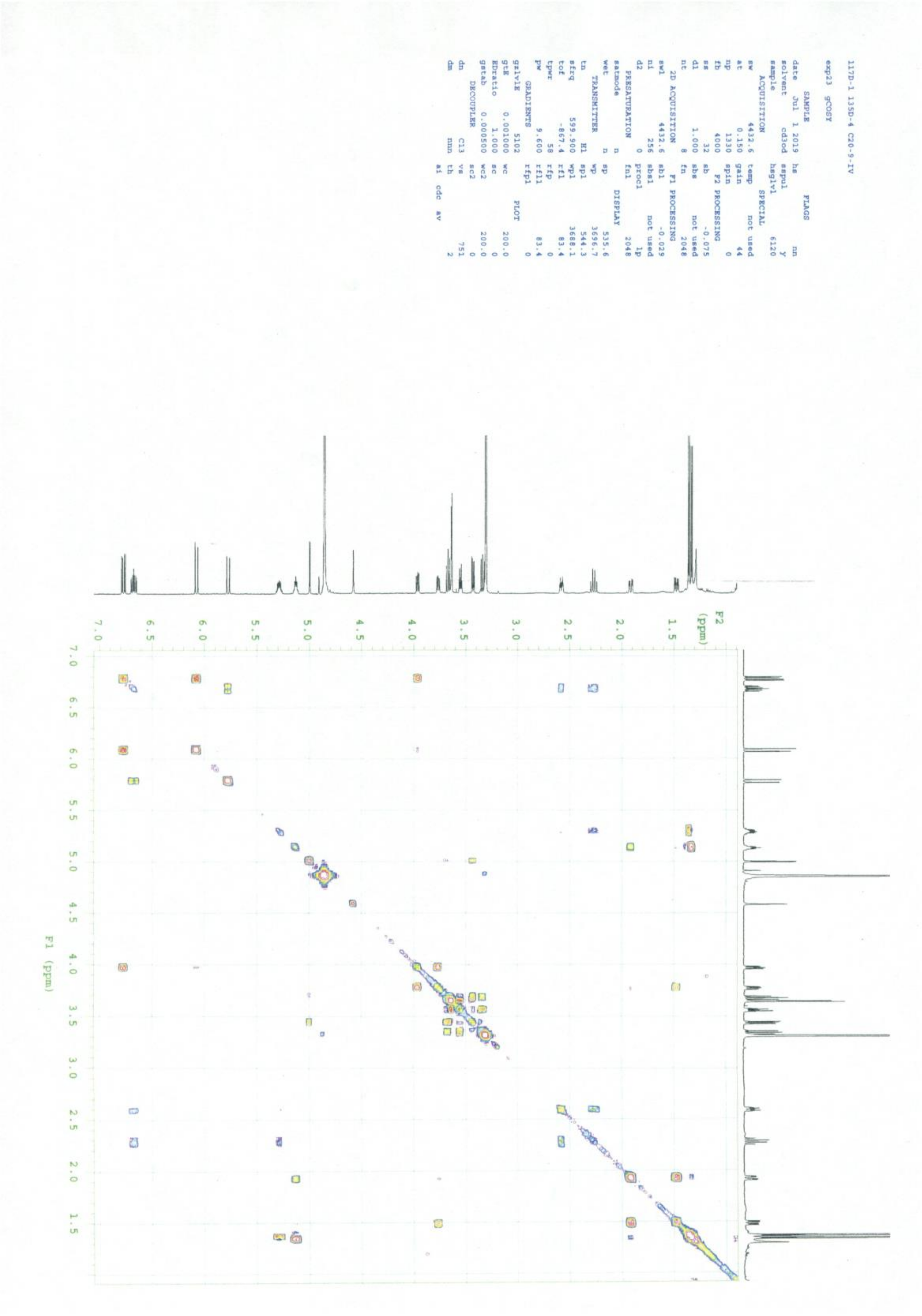

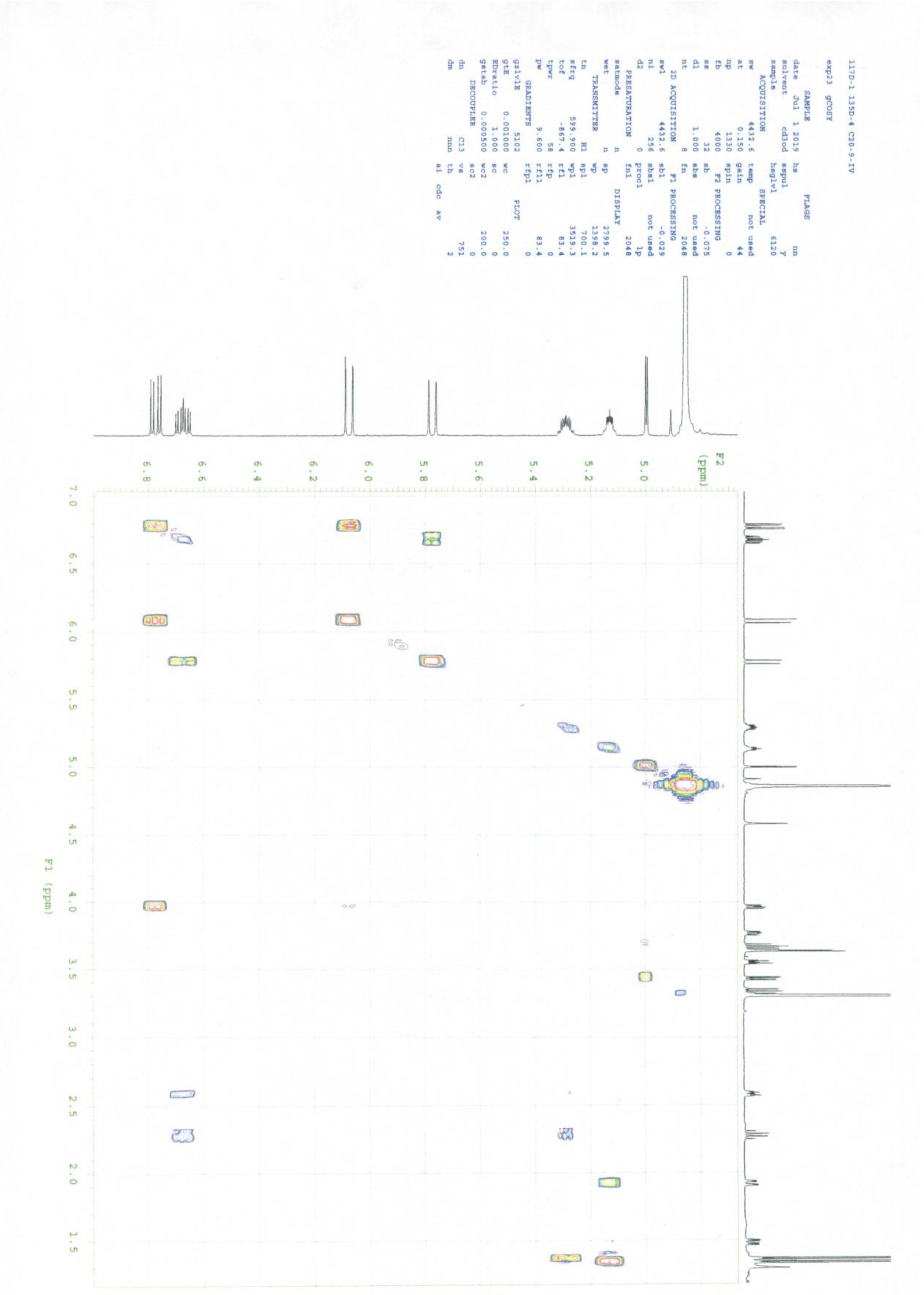
117D-1 135D-4 C20-9-IV
exp23 gCOSY
F2 (ppm)
F1 (ppm)

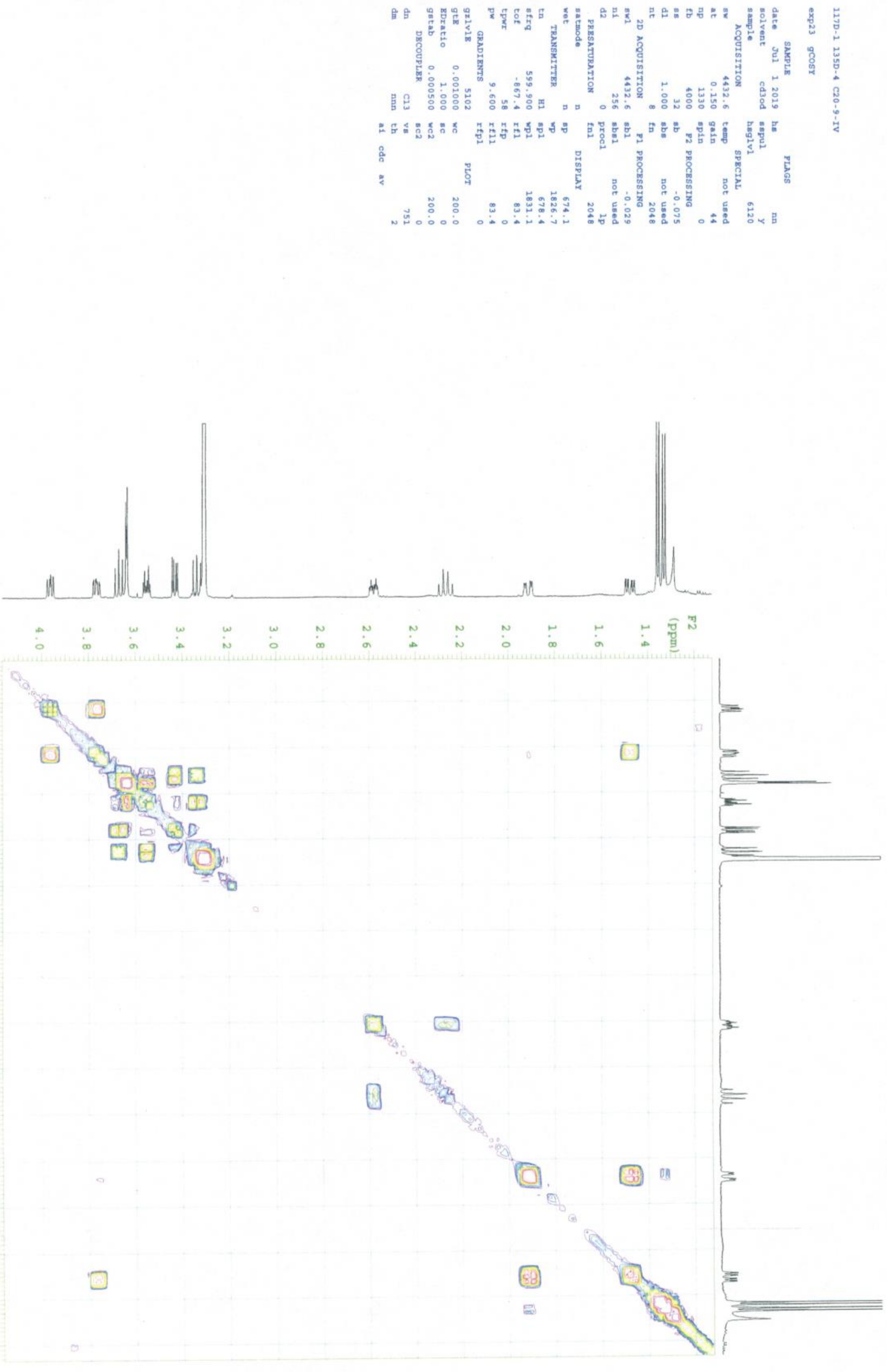
117D-1 135D-4 C20-9-IV
exp23 gCOSY
SAMPLE
date Jul 1 2019
solvent cd3od
sample
ACQUISITION
sw 4432.6
at 0.150
np 1330
fb 4000
ss 32
d1 1.000
nt 8
2D ACQUISITION
sw1 4432.6
ni 256
d2 0
PRESATURATION
satmode n
wet n
TRANSMITTER
tn H1
sfrq 599.900
tof -867.4
tpwr 58
pw 9.600
GRADIENTS
gzlvlE 5102
gtE 0.001000
EDratio 1.000
gstab 0.000500
DECOUPLER
dn C13
dm nnn
FLAGS
hs nn
sspul y
hsglvl 6120
SPECIAL
temp not used
gain 44
spin 0
F2 PROCESSING
sb -0.075
sbs not used
fn 2048
F1 PROCESSING
sb1 -0.029
sbs1 not used
proc1 lp
fn1 2048
DISPLAY
sp 674.1
wp 1826.7
sp1 678.4
wp1 1831.1
rfl 83.4
rfp 0
rfl1 83.4
rfp1 0
PLOT
wc 200.0
sc 0
wc2 200.0
sc2 0
vs 751
th 2
ai cdc av
F2 (ppm)
F1 (ppm)

Figure S13 NOESY of 3

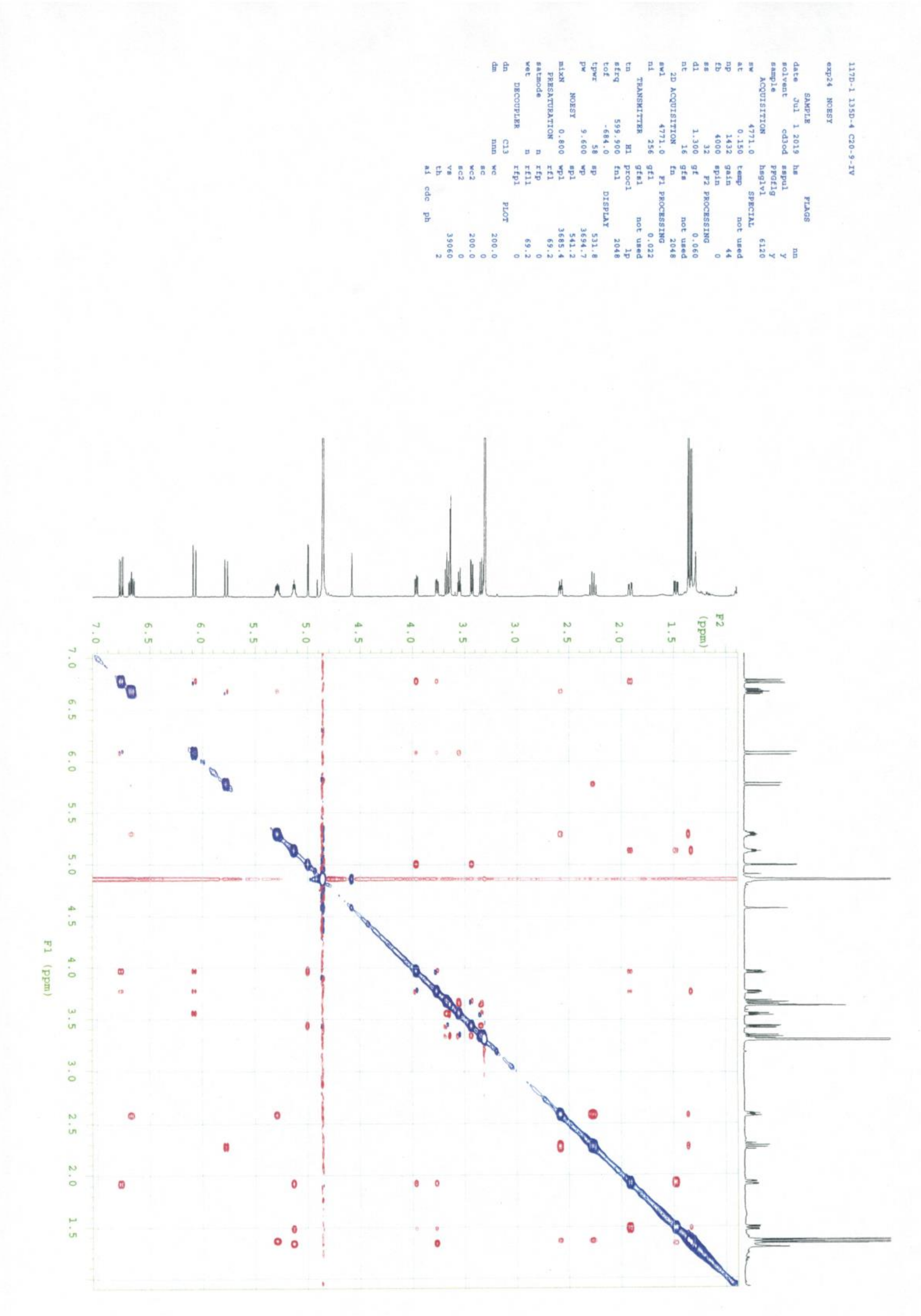

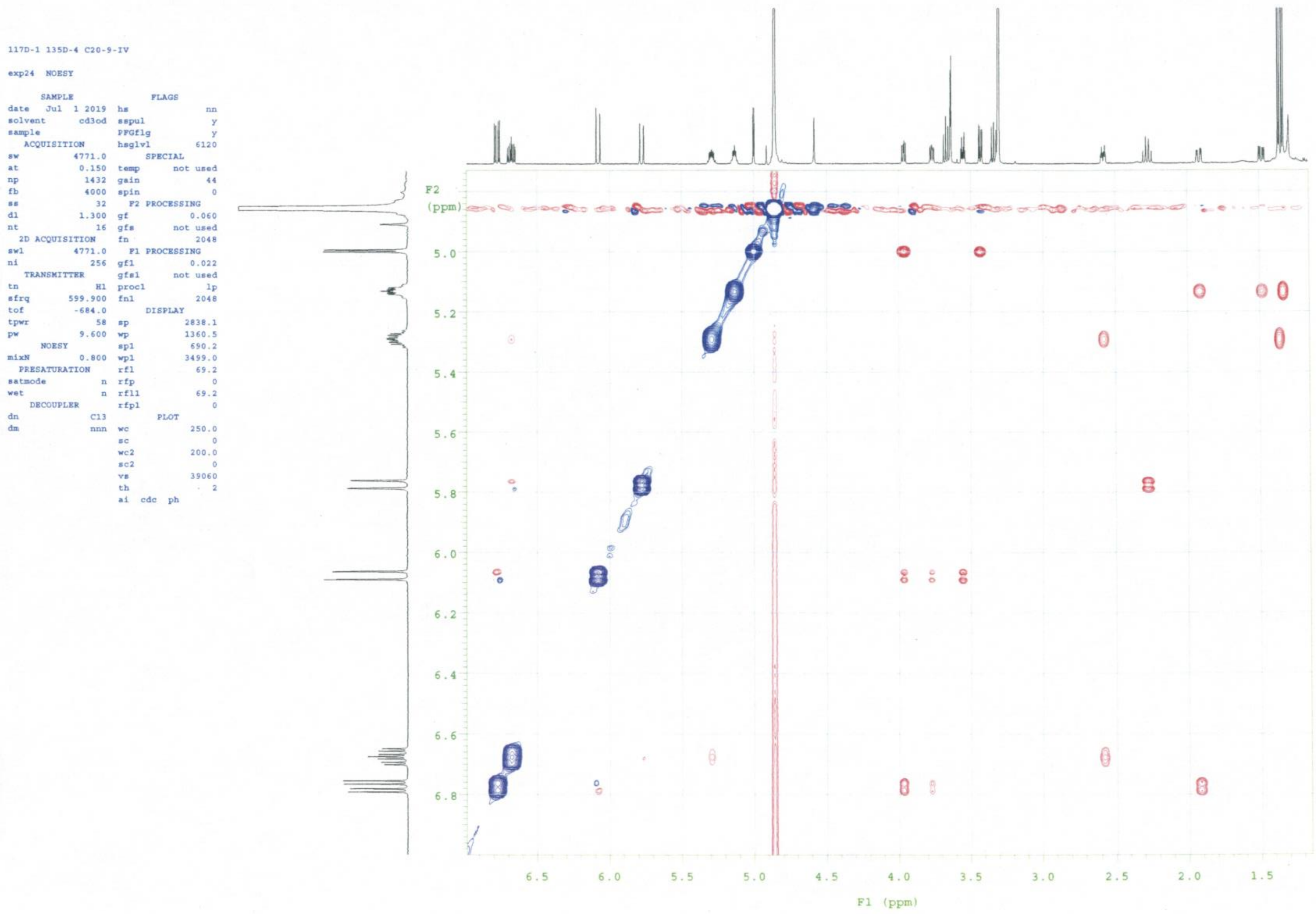
117D-1 135D-4 C20-9-IV
exp24 NOESY
SAMPLE
date Jul 1 2019
solvent cd3od
sample
ACQUISITION
sw 4771.0
at 0.150
np 1432
fb 4000
ss 32
d1 1.300
nt 16
2D ACQUISITION
sw1 4771.0
ni 256
TRANSMITTER
tn H1
sfrq 599.900
tof -684.0
tpwr 58
pw 9.600
NOESY
mixN 0.800
PRESATURATION
satmode n
wet n
DECOUPLER
dn C13
dm nnn
FLAGS
hs nn
sspul y
PFGflg y
hsglvl 6120
SPECIAL
temp not used
gain 44
spin 0
F2 PROCESSING
gf 0.060
gfs not used
fn 2048
F1 PROCESSING
gf1 0.022
gfs1 not used
procl lp
fn1 2048
DISPLAY
sp 2838.1
wp 1360.5
sp1 690.2
wp1 3499.0
rfl 69.2
rfp 0
rfl1 69.2
rfp1 0
PLOT
wc 250.0
sc 0
wc2 200.0
sc2 0
vs 39060
th 2
ai cdc ph
F2 (ppm)
5.0
5.2
5.4
5.6
5.8
6.0
6.2
6.4
6.6
6.8
6.5
6.0
5.5
5.0
4.5
4.0
3.5
3.0
2.5
2.0
1.5
F1 (ppm)

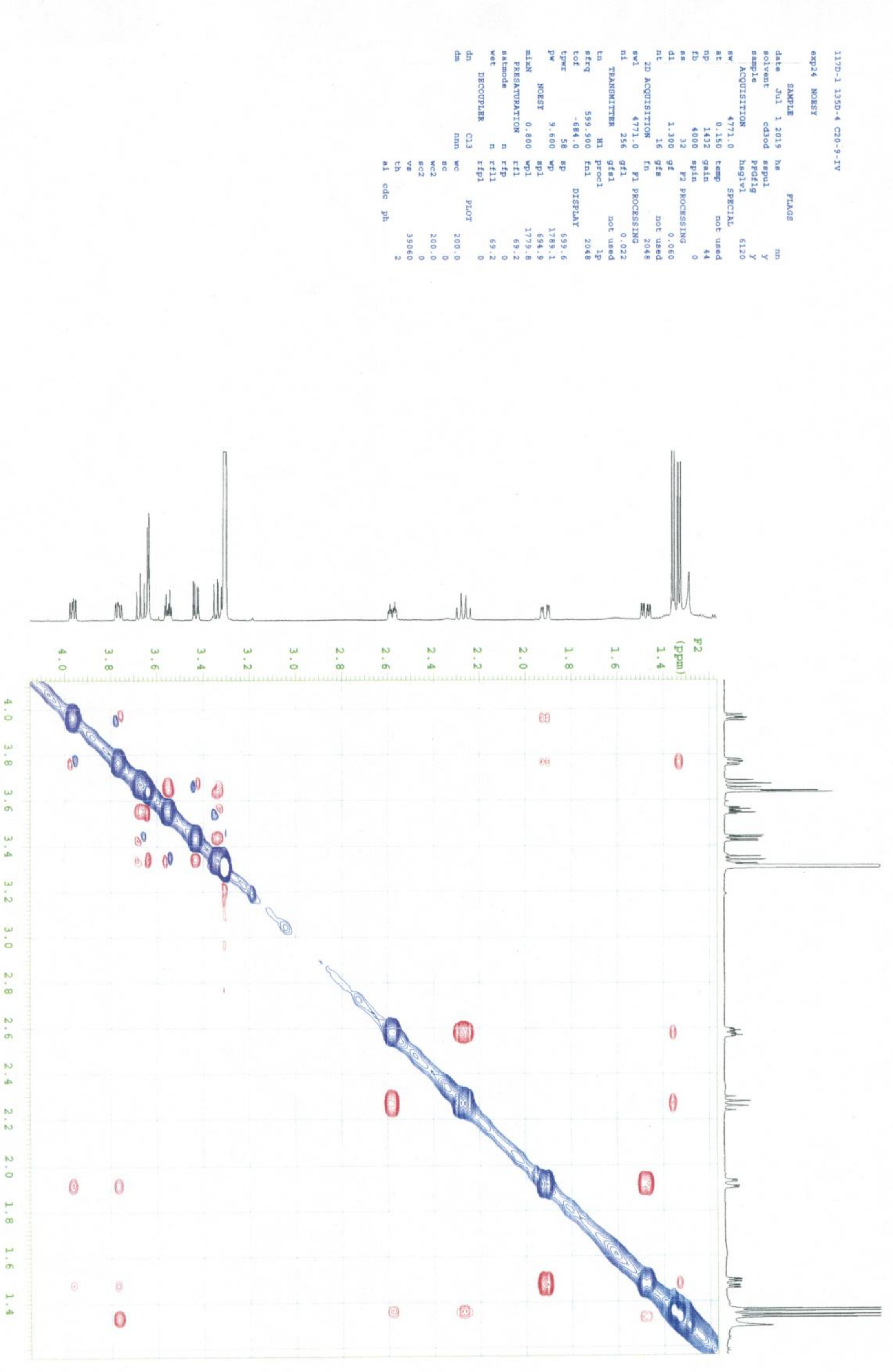
117D-1 135D-4 C20-9-IV
exp24 NOESY
SAMPLE
date Jul 1 2019
solvent cd3od
sample
ACQUISITION
sw 4771.0
at 0.150
np 1432
fb 4000
ss 32
d1 1.300
nt 16
2D ACQUISITION
sw1 4771.0
ni 256
TRANSMITTER
tn H1
sfrq 599.900
tof -684.0
tpwr 58
pw 9.600
NOESY
mixN 0.800
PRESATURATION
satmode n
wet n
DECOUPLER
dn C13
dm nnn
FLAGS
hs nn
sspul y
PFGflg y
hsglvl 6120
SPECIAL
temp not used
gain 44
spin 0
F2 PROCESSING
gf 0.060
gfs not used
fn 2048
F1 PROCESSING
gf1 0.022
gfs1 not used
proc1 lp
fn1 2048
DISPLAY
sp 699.6
wp 1789.1
sp1 694.9
wp1 1779.8
rfl 69.2
rfp 0
rfl1 69.2
rfp1 0
PLOT
wc 200.0
sc 0
wc2 200.0
sc2 0
vs 39060
th 2
ai cdc ph
F2 (ppm)
F1 (ppm)

Figure S14 HMQC of 3

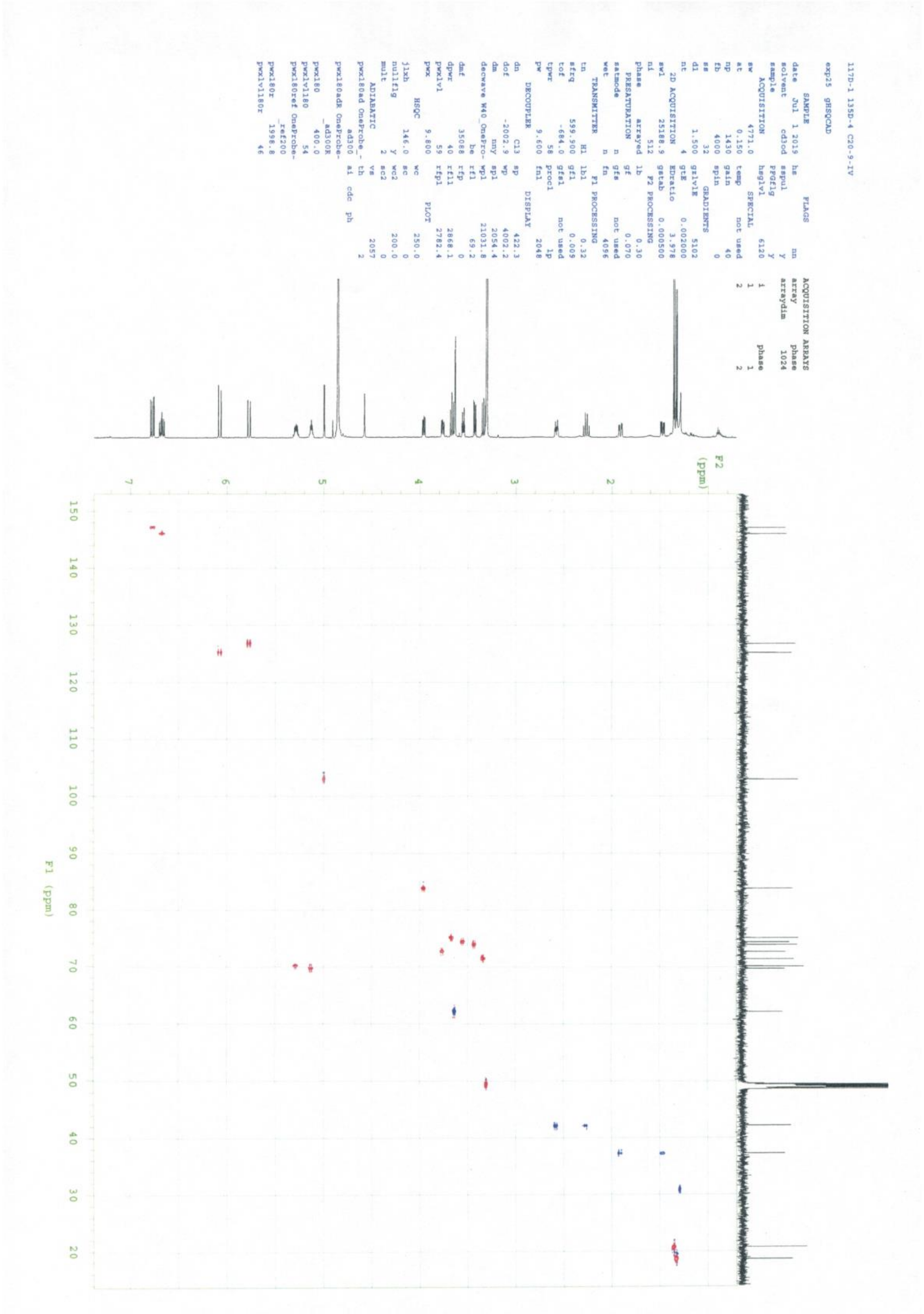

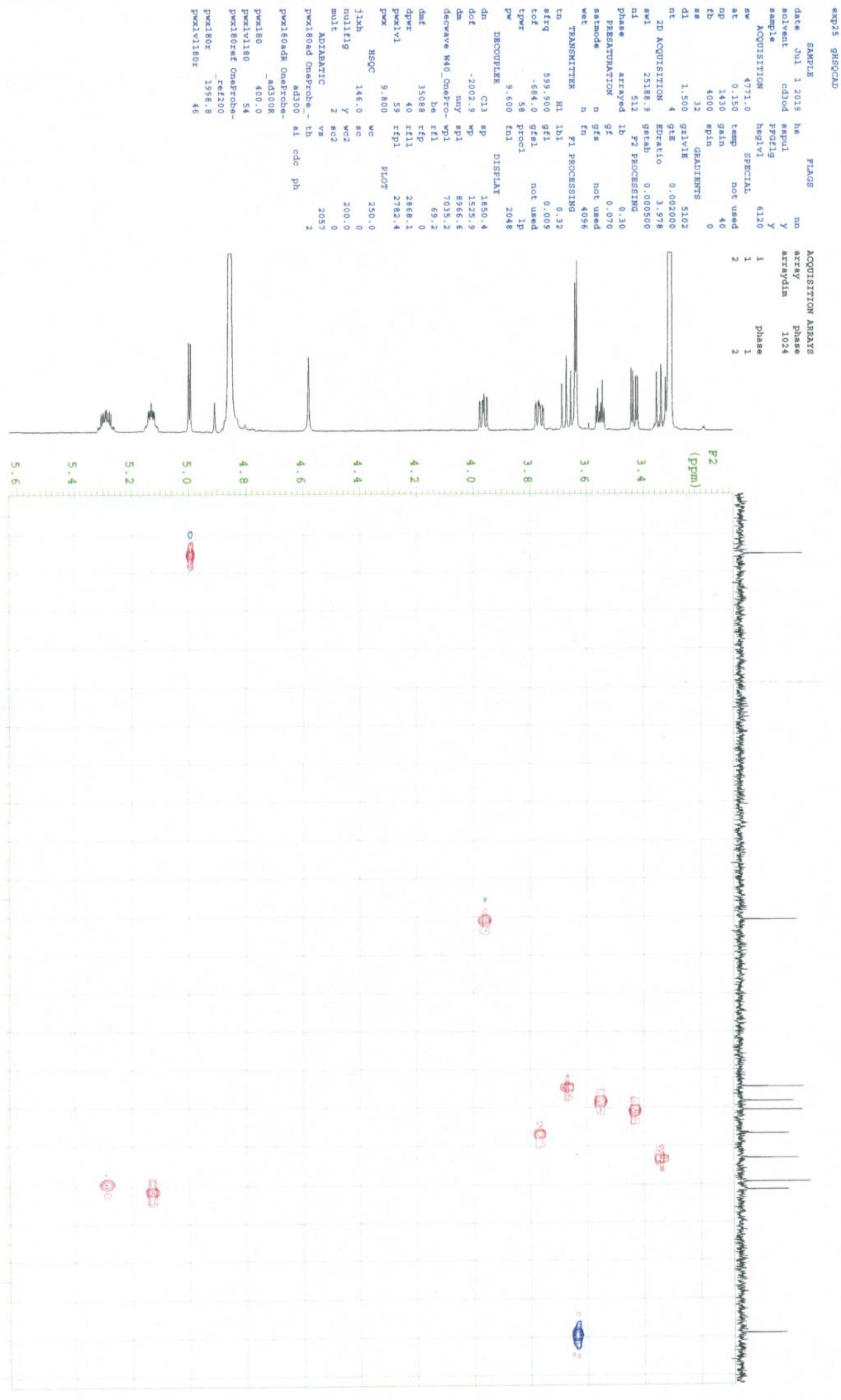
117D-1 135D-4 C20-9-IV
exp25 gHSQCAD
SAMPLE
date Jul 1 2019
solvent cd3od
sample
ACQUISITION
sw 4771.0
at 0.150
np 1430
fb 4000
ss 32
d1 1.500
nt 4
2D ACQUISITION
sw1 25188.9
ni 512
phase arrayed
PRESATURATION
satmode n
wet n
TRANSMITTER
tn H1
sfrq 599.900
tof -684.0
tpwr 58
pw 9.600
DECOUPLER
dn C13
dof -2002.9
dm nny
decwave W40_OnePro-be
dmf 35088
dpwr 40
pwxlvl 59
pwx 9.800
HSQC
j1xh 146.0
nullflg y
mult 2
ADIABATIC
pwx180ad OneProbe_-ad300
pwx180adR OneProbe-_ad300R
pwx180 400.0
pwxlvl180 54
pwx180ref OneProbe-_ref200
pwx180r 1998.8
pwxlvl180r 46
FLAGS
hs nn
sspul y
PFGflg y
hsglvl 6120
SPECIAL
temp not used
gain 40
spin 0
GRADIENTS
gzlvlE 5102
gtE 0.002000
EDratio 3.978
gstab 0.000500
F2 PROCESSING
lb 0.30
gf 0.070
gfs not used
fn 4096
F1 PROCESSING
lb1 0.32
gf1 0.009
gfs1 not used
proc1 lp
fn1 2048
DISPLAY
sp 1850.4
wp 1525.9
sp1 8966.6
wp1 7035.2
rfl 69.2
rfp 0
rfl1 2868.1
rfp1 2782.4
PLOT
wc 250.0
sc 0
wc2 200.0
sc2 0
vs 2057
th 2
ai cdc ph
ACQUISITION ARRAYS
array phase
arraydim 1024
i phase
1 1
2 2
F2 (ppm)
3.4
3.6
3.8
4.0
4.2
4.4
4.6
4.8
5.0
5.2
5.4
5.6
105
100
95
90
85
80
75
70
65
F1 (ppm)

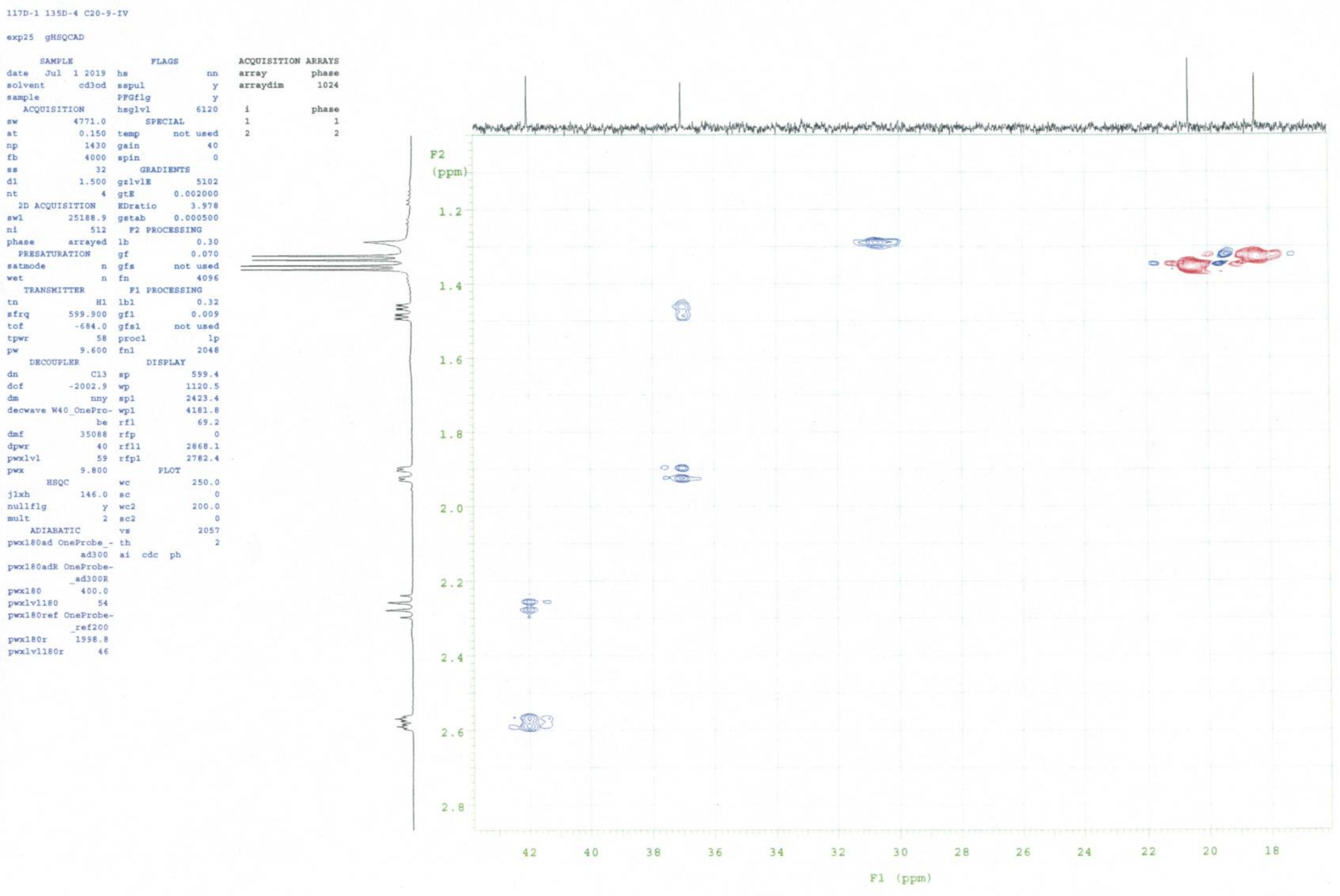
117D-1 135D-4 C20-9-IV
exp25 gHSQCAD
SAMPLE
date Jul 1 2019
solvent cd3od
sample
ACQUISITION
sw 4771.0
at 0.150
np 1430
fb 4000
ss 32
d1 1.500
nt 4
2D ACQUISITION
sw1 25188.9
ni 512
phase arrayed
PRESATURATION
satmode n
wet n
TRANSMITTER
tn H1
sfrq 599.900
tof -684.0
tpwr 58
pw 9.600
DECOUPLER
dn C13
dof -2002.9
dm nny
decwave W40_OnePro-be
dmf 35088
dpwr 40
pwxlvl 59
pwx 9.800
HSQC
j1xh 146.0
nullflg y
mult 2
ADIABATIC
pwx180ad OneProbe_-ad300
pwx180adR OneProbe-_ad300R
pwx180 400.0
pwxlvl180 54
pwx180ref OneProbe-_ref200
pwx180r 1998.8
pwxlvl180r 46
FLAGS
hs nn
sspul y
PFGflg y
hsglvl 6120
SPECIAL
temp not used
gain 40
spin 0
GRADIENTS
gzlvlE 5102
gtE 0.002000
EDratio 3.978
gstab 0.000500
F2 PROCESSING
lb 0.30
gf 0.070
gfs not used
fn 4096
F1 PROCESSING
lb1 0.32
gf1 0.009
gfs1 not used
proc1 lp
fn1 2048
DISPLAY
sp 599.4
wp 1120.5
sp1 2423.4
wp1 4181.8
rf1 69.2
rfp 0
rfl1 2868.1
rfp1 2782.4
PLOT
wc 250.0
sc 0
wc2 200.0
sc2 0
vs 2057
th 2
ai cdc ph
ACQUISITION ARRAYS
array phase
arraydim 1024
i phase
1 1
2 2
F2 (ppm)
1.2
1.4
1.6
1.8
2.0
2.2
2.4
2.6
2.8
42
40
38
36
34
32
30
28
26
24
22
20
18
F1 (ppm)

Figure S15 HMBC of 3

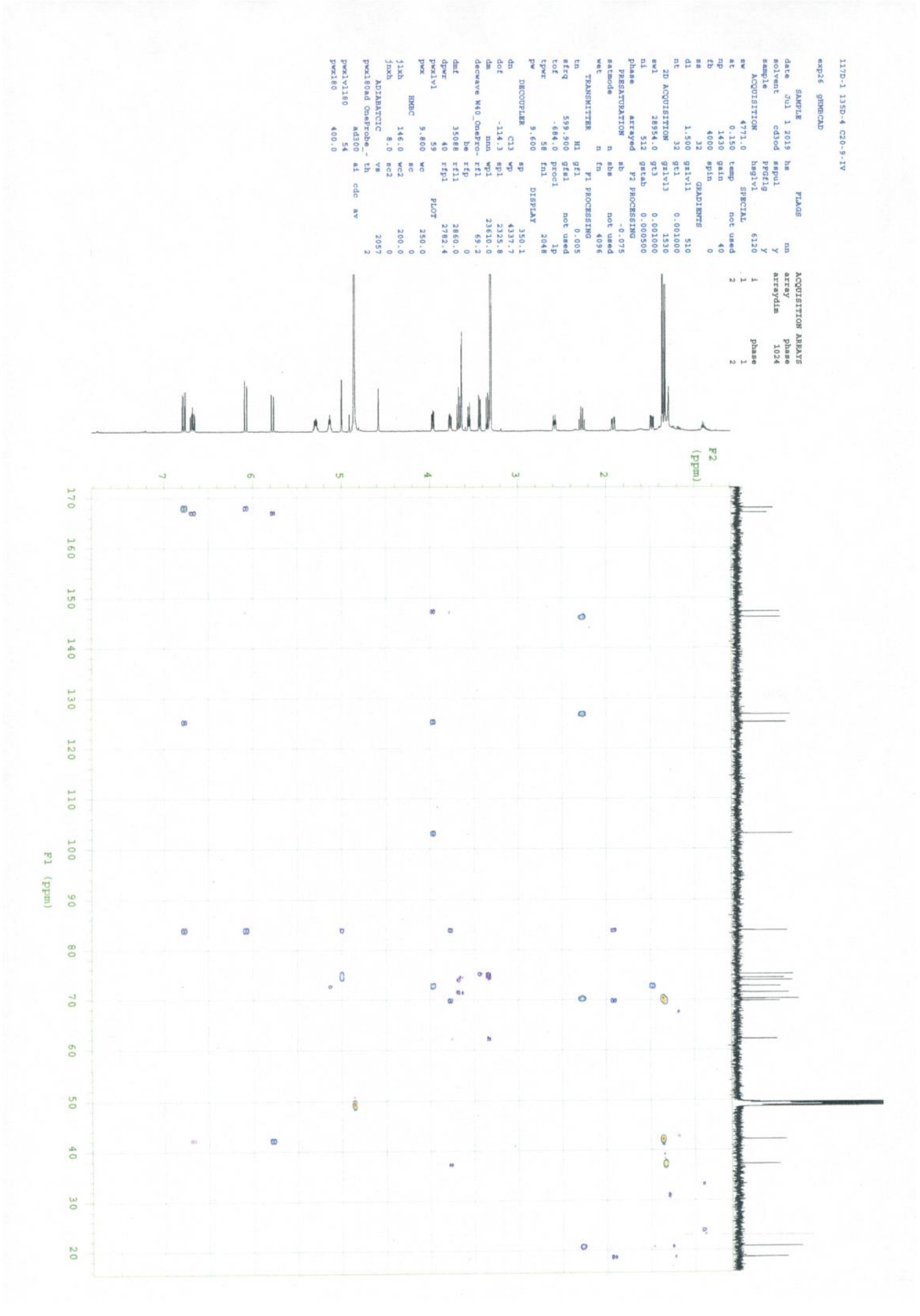

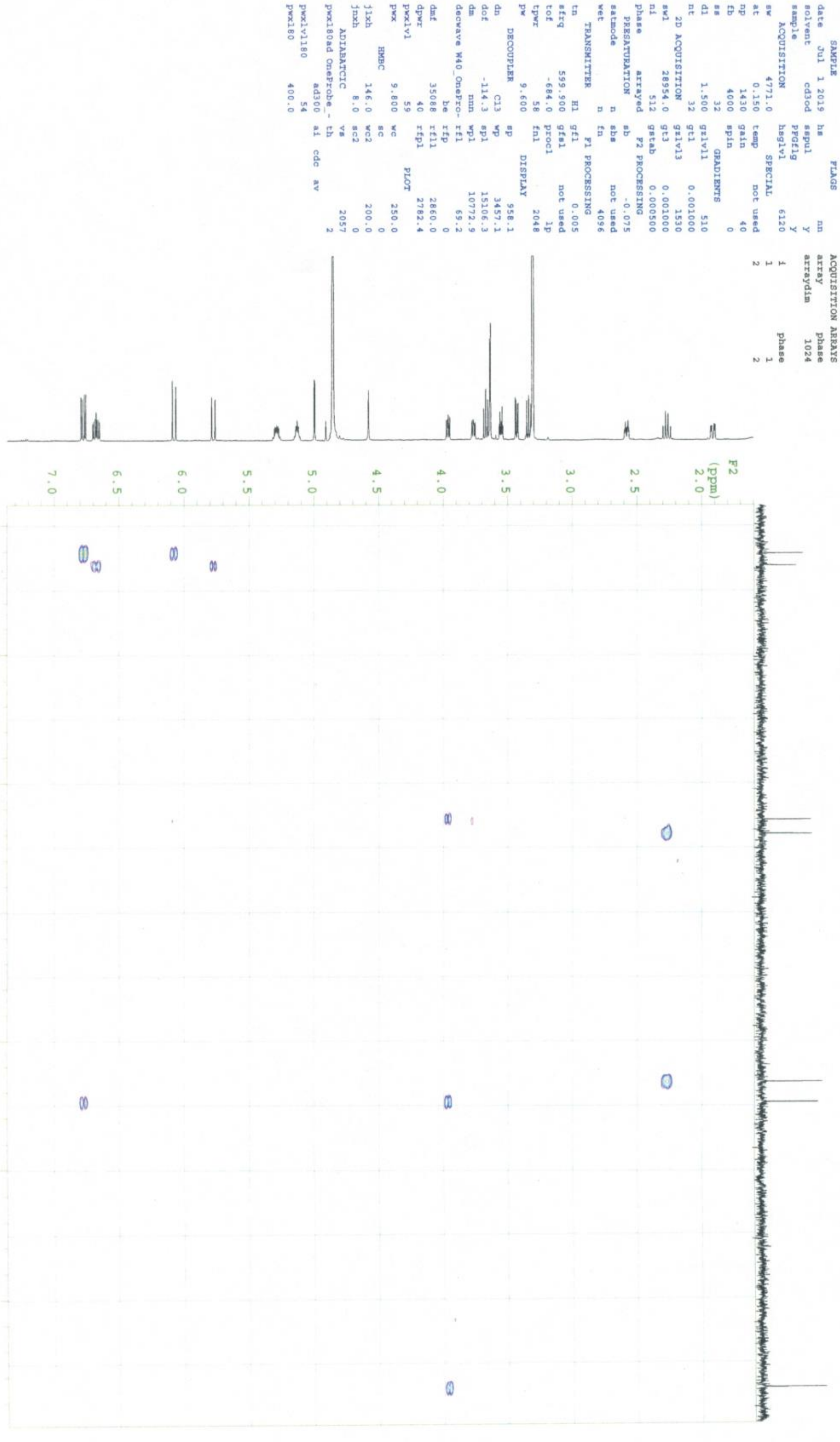

117D-1 135D-4 C20-9-IV
exp26 gHMBCAD
SAMPLE
date Jul 1 2019
solvent cd3od
sample
ACQUISITION
sw 4771.0
at 0.150
np 1430
fb 4000
ss 32
d1 1.500
nt 32
2D ACQUISITION
sw1 28954.0
ni 512
phase arrayed
PRESATURATION
satmode n
wet n
TRANSMITTER
tn H1
sfrq 599.900
tof -684.0
tpwr 58
pw 9.600
DECOUPLER
dn C13
dof -114.3
dm nnn
decwave W40_OnePro-
be
dmf 35088
dpwr 40
pwxlvl 59
pwx 9.800
HMBC
j1xh 146.0
jnxh 8.0
ADIABATCIC
pwx180ad OneProbe_-
ad300
pwxlvl180 54
pwx180 400.0
FLAGS
hs nn
sspul y
PFGflg y
hsglvl 6120
SPECIAL
temp not used
gain 40
spin 0
GRADIENTS
gzlvl1 510
gt1 0.001000
gzlvl3 1530
gt3 0.001000
gstab 0.000500
F2 PROCESSING
sb -0.075
sbs not used
fn 4096
F1 PROCESSING
gf1 0.005
gfs1 not used
proc1 lp
fn1 2048
DISPLAY
sp 958.1
wp 3457.1
sp1 15106.3
wp1 10772.9
rfl 69.2
rfp 0
rfl1 2860.0
rfp1 2782.4
PLOT
wc 250.0
sc 0
wc2 200.0
sc2 0
vs 2057
th 2
ai cdc av
ACQUISITION ARRAYS
array phase
arraydim 1024
i phase
1 1
2 2
F2 (ppm)
2.0
2.5
3.0
3.5
4.0
4.5
5.0
5.5
6.0
6.5
7.0
170
160
150
140
130
120
110
F1 (ppm)

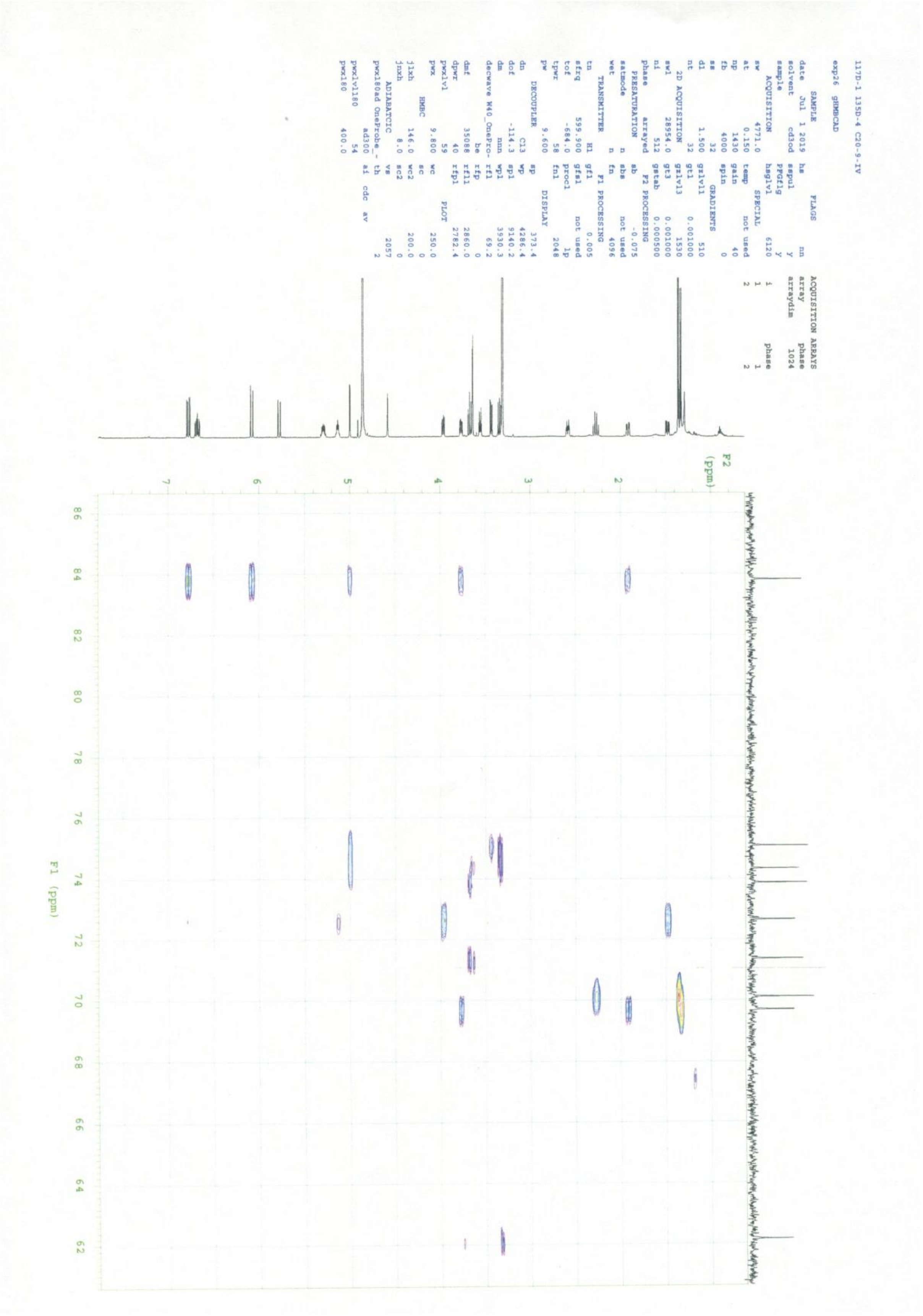
117D-1 135D-4 C20-9-IV
exp26 gHMBCAD
F2 (ppm)
F1 (ppm)

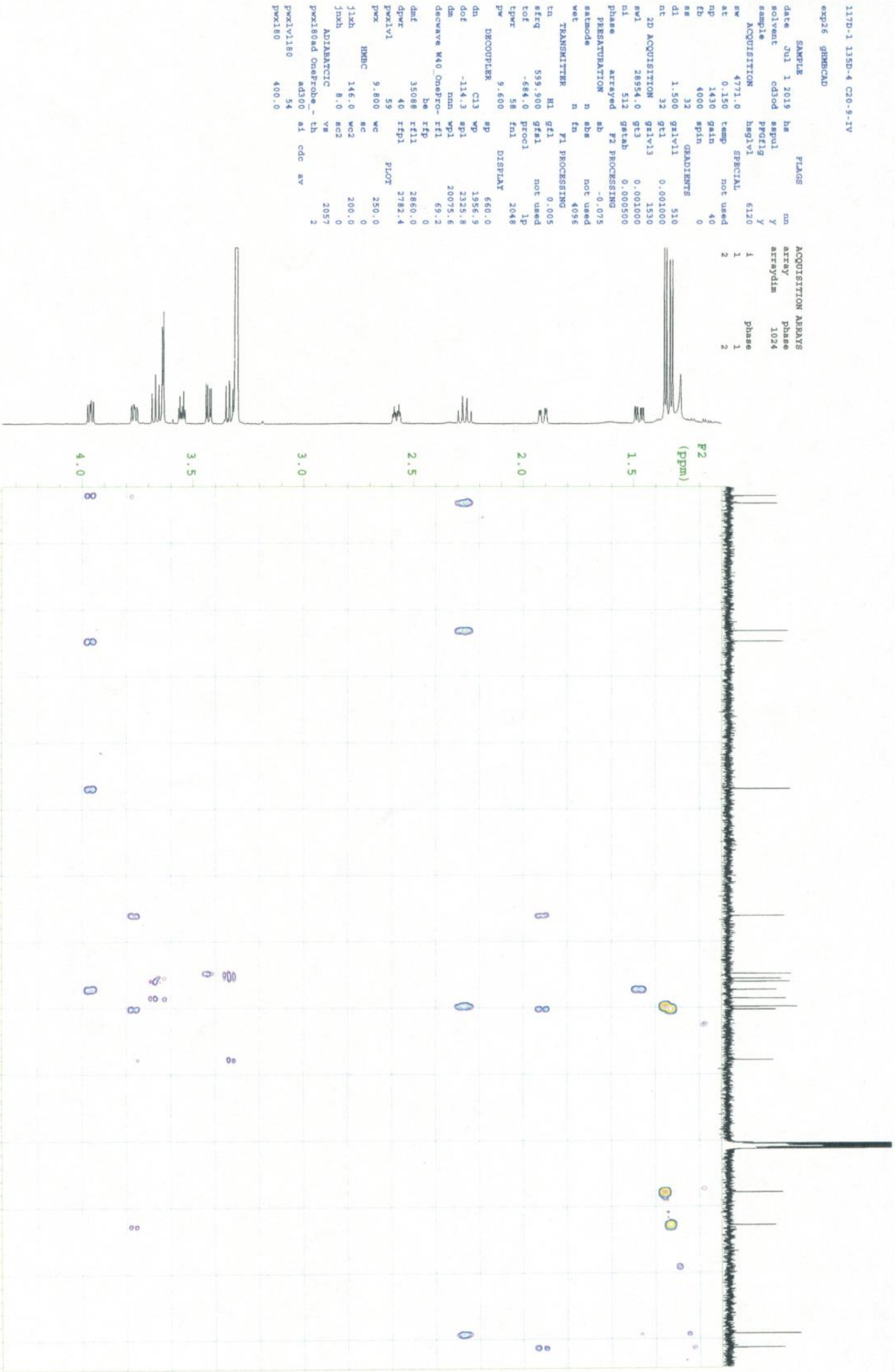
117D-1 135D-4 C20-9-IV
exp26 gHMBCAD
SAMPLE
date Jul 1 2019
solvent cd3od
sample
ACQUISITION
sw 4771.0
at 0.150
np 1430
fb 4000
ss 32
d1 1.500
nt 32
2D ACQUISITION
sw1 28954.0
ni 512
phase arrayed
PRESATURATION
satmode n
wet n
TRANSMITTER
tn H1
sfrq 599.900
tof -684.0
tpwr 58
pw 9.600
DECOUPLER
dn C13
dof -114.3
dm nnn
decwave W40_OnePro-
be
dmf 35088
dpwr 40
pwxlvl 59
pwx 9.800
HMBC
j1xh 146.0
jnxh 8.0
ADIABATCIC
pwx180ad OneProbe_-
ad300
pwxlvl180 54
pwx180 400.0
FLAGS
hs nn
sspul y
PFGflg y
hsglvl 6120
SPECIAL
temp not used
gain 40
spin 0
GRADIENTS
gzlvl1 510
gt1 0.001000
gzlvl3 1530
gt3 0.001000
gstab 0.000500
F2 PROCESSING
sb -0.075
sbs not used
fn 4096
F1 PROCESSING
gf1 0.005
gfs1 not used
proc1 lp
fn1 2048
DISPLAY
sp 660.0
wp 1956.9
sp1 2325.8
wp1 20075.6
rfl 69.2
rfp 0
rfl1 2860.0
rfp1 2782.4
PLOT
wc 250.0
sc 0
wc2 200.0
sc2 0
vs 2057
th 2
ai cdc av
ACQUISITION ARRAYS
array phase
arraydim 1024
i phase
1 1
2 2
F2 (ppm)
1.5
2.0
2.5
3.0
3.5
4.0
140
130
120
110
100
90
80
70
60
50
40
30
20
F1 (ppm)

Figure S16 FABMS of 3

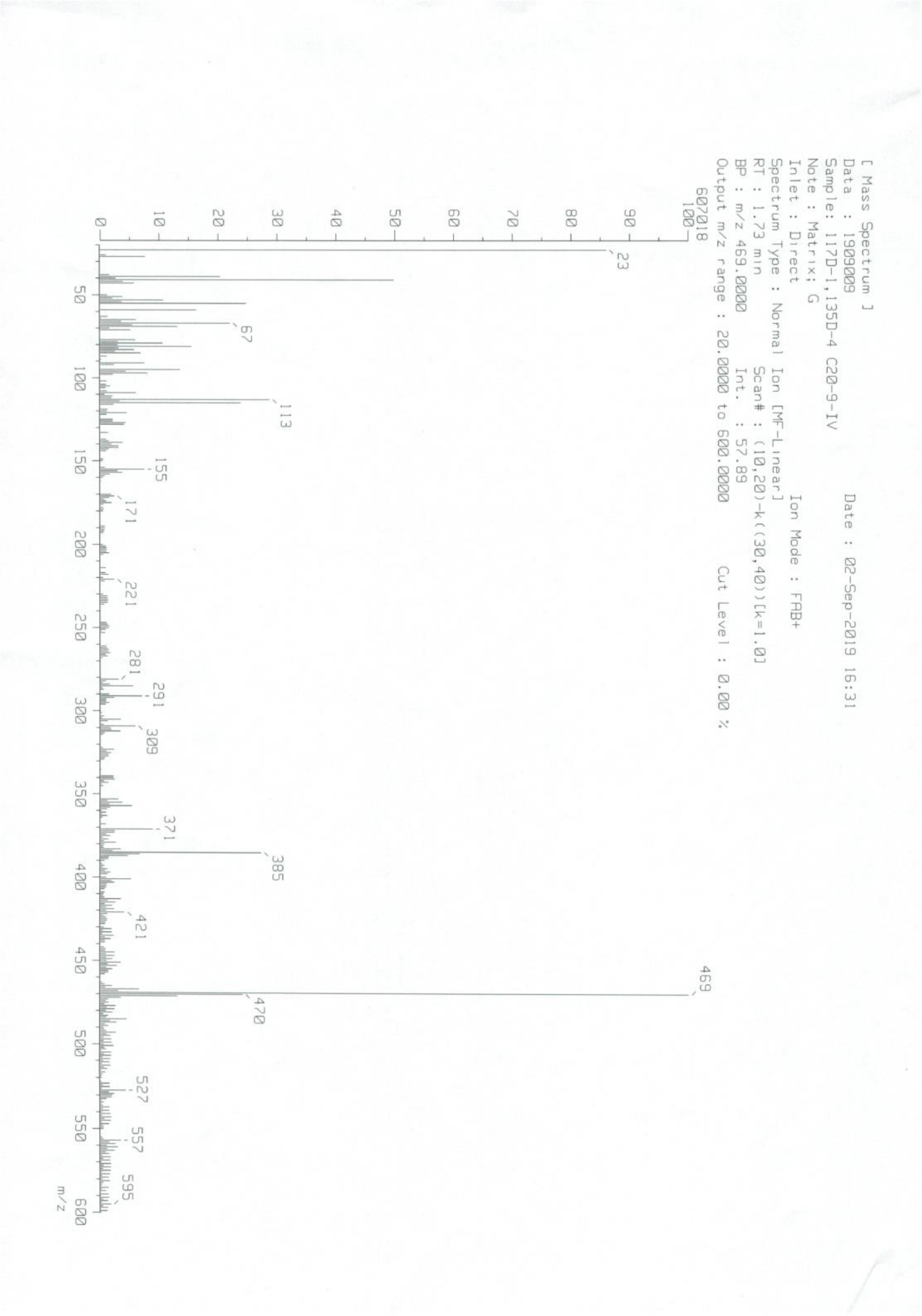

[Mass Spectrum] Page: 1
Data : 1909009 Date : 02-Sep-2019 16:31
Sample: 117D-1,135D-4 C20-9-IV
Note : Matrix; G
Inlet : Direct Ion Mode : FAB+
Spectrum Type : Normal Ion [MF-Linear]
RT : 1.73 min Scan# : (10,20)-k((30,40))[k=1.0]
BP : m/z 469.0000 Int. : 57.89
Output m/z range : 20.0000 to 600.0000 Cut Level : 3.00 %

m/z	Int.	Norm.	m/z	Int.	Norm.
23.0000	49.79	86.02	387.0000	2.68	4.63
27.0000	4.40	7.60	401.0000	2.97	5.14
39.0000	11.78	20.35	413.0000	1.99	3.43
41.0000	28.83	49.80	421.0000	2.33	4.03
42.0000	2.21	3.82	451.0000	1.97	3.41
43.0000	3.32	5.74	467.0000	3.79	6.55
51.0000	2.16	3.73	468.0000	1.74	3.00
53.0000	6.19	10.69	469.0000	57.89	100.00
54.0000	2.09	3.61	470.0000	13.96	24.12
55.0000	14.31	24.71	471.0000	7.52	12.99
59.0000	9.43	16.28	472.0000	1.97	3.41
65.0000	3.51	6.06	485.0000	2.56	4.43
66.0000	2.06	3.56	527.0000	2.45	4.24
67.0000	12.72	21.98	557.0000	1.96	3.39
68.0000	3.14	5.42			
69.0000	7.57	13.08			
71.0000	2.95	5.10			
77.0000	3.44	5.94			
79.0000	6.12	10.57			
81.0000	8.94	15.45			
82.0000	1.76	3.05			
83.0000	3.32	5.73			
85.0000	3.97	6.85			
91.0000	4.32	7.46			
95.0000	7.84	13.55			
96.0000	2.50	4.31			
97.0000	4.64	8.02			
109.0000	3.48	6.02			
113.0000	16.60	28.67			
114.0000	1.88	3.24			
115.0000	13.78	23.80			
121.0000	2.59	4.47			
127.0000	2.47	4.27			
128.0000	2.33	4.02			
139.0000	2.19	3.79			
141.0000	1.74	3.01			
142.0000	1.76	3.04			
155.0000	4.31	7.44			
157.0000	2.14	3.69			
285.0000	3.17	5.47			
291.0000	4.05	6.99			
305.0000	1.96	3.39			
309.0000	3.39	5.85			
312.0000	1.90	3.29			
355.0000	2.12	3.66			
357.0000	3.07	5.30			
371.0000	5.10	8.82			
383.0000	1.96	3.39			
385.0000	15.74	27.18			
386.0000	3.83	6.61			

[Elemental Composition] Page: 1

Data : 1909155 Date : 18-Sep-2019 16:52
Sample: 117D-1,135D-4 C20-9-IV
Note : Matrix; G
Inlet : Direct Ion Mode : FAB+
RT : 8.37 min Scan#: (75,80)
Elements : C 25/15, H 35/25, O 15/0, Na 1/0
Mass Tolerance : 20ppm, 1mmu if m/z > 50
Unsaturation (U.S.) : -1.0 - 40.0

Observed m/z	Int%	Err[ppm / mmu]	U.S.	Composition
469.1692	100.0	+1.2 / +0.6	5.5	C 20 H 30 O 11 Na

469.1677

Figure S17 IR spectrum of 3

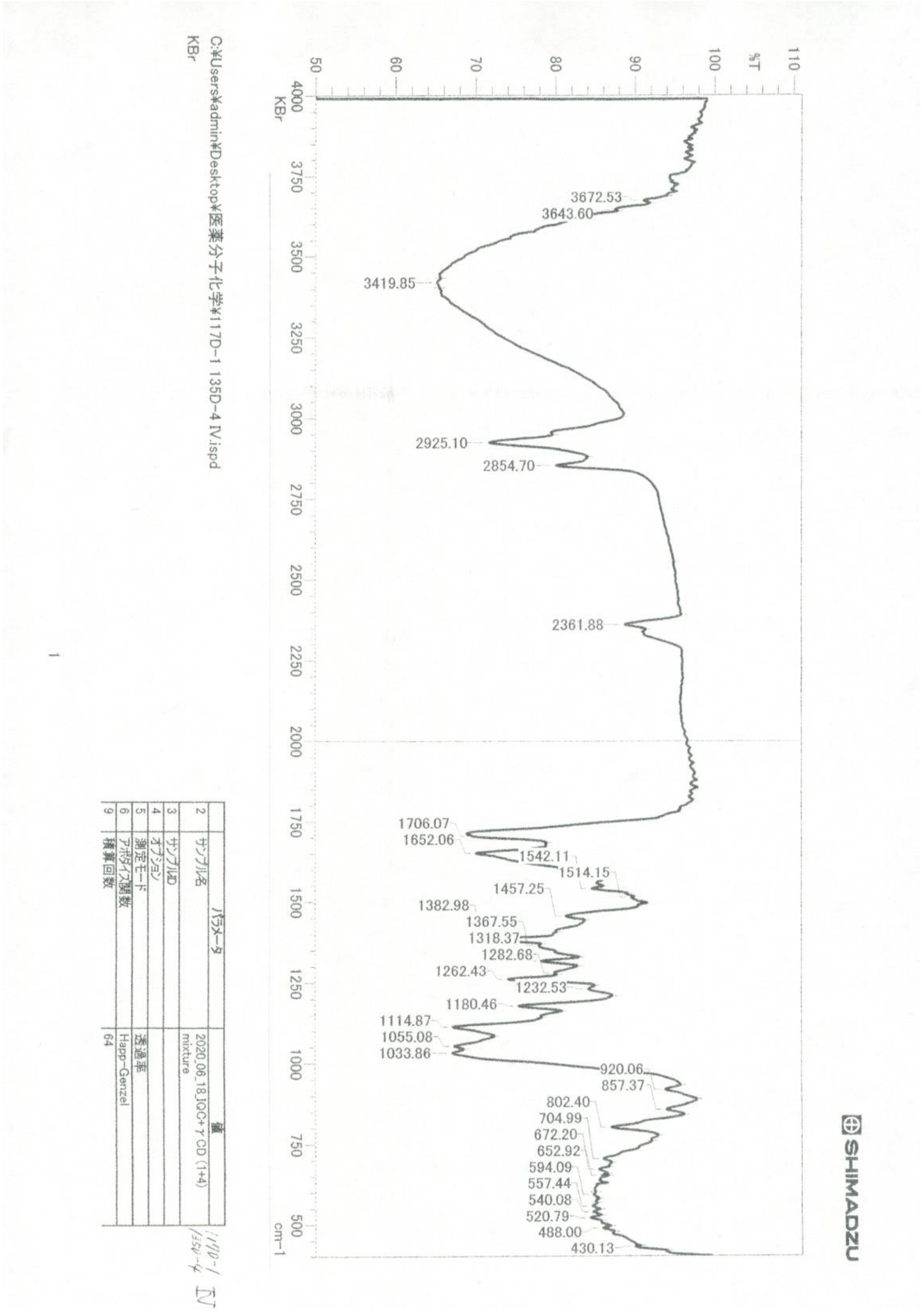

Figure S18 HPLC chromatograms of halosmycins A, B, and C

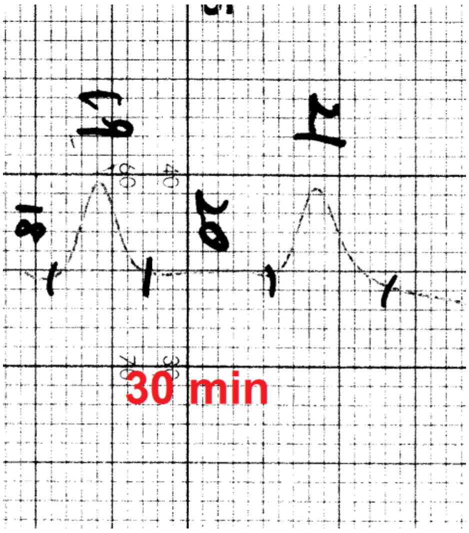

Peak 19: halosmycins B (r.t. 28.8 min)

Peak 21: halosmycins A (r.t. 31.7 min)

Column: Cosmosil Packed Column 5C18-MSII (25 cm X 20 mm i.d.)

Mobile phase: 80% MeOH in H_2O

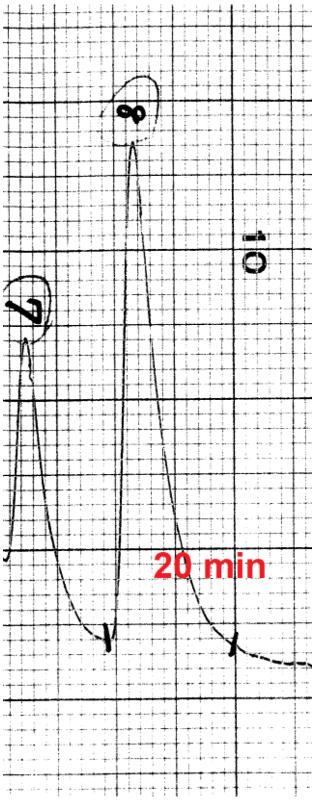

Peak 8: halosmycins C (r.t. 18.3 min)

Column: Cosmosil Packed Column 5C18-MSII (25 cm X 20 mm i.d.)

Mobile phase: 80% MeOH in H_2O

Figure S19 ^{1}H NMR spectrum of 5-hydroxy-(2*E*)-hexenoic acid in MeOH-*d*$_4$

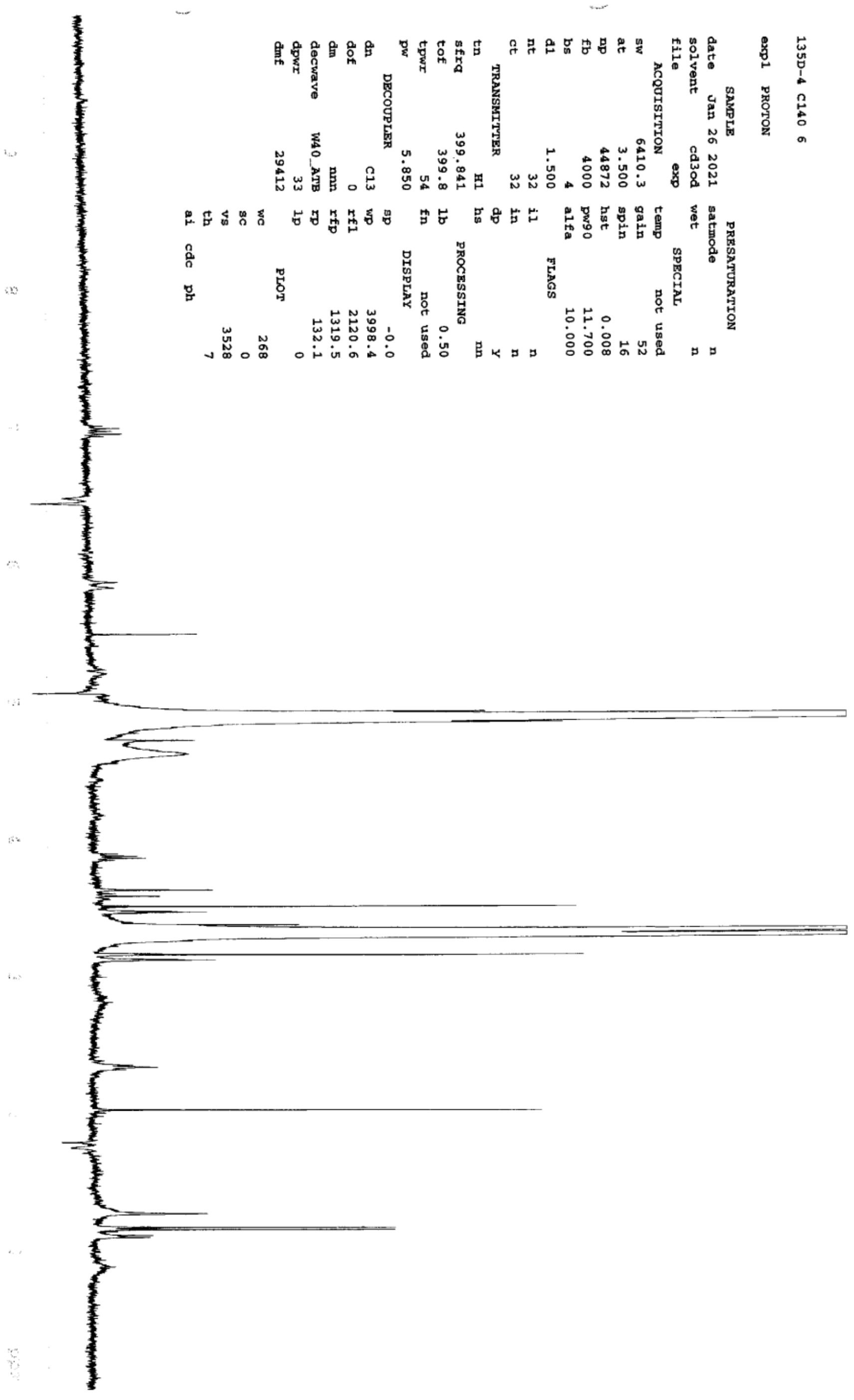

Figure S20 ^{1}H NMR spectrum of 4,5,7-trihydroxy-(2*E*)-octenoic acid in MeOH-*d*$_4$

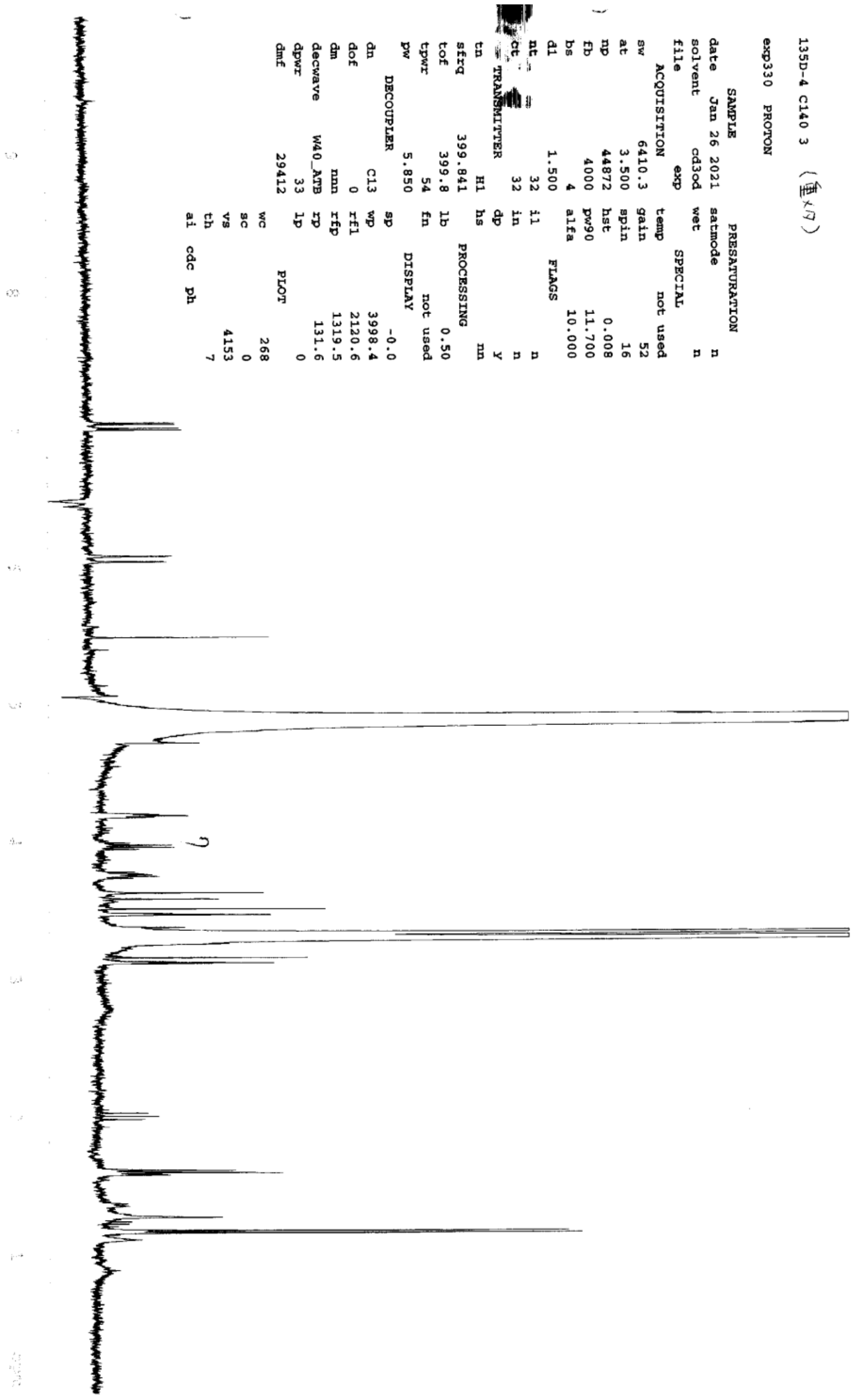

Supplement: Supplementary file 1 [file marinedrugs-20-00226-s001.zip › marinedrugs-1623648-supplementary.pdf]
